# The onset of deep recycling of supracrustal materials at the Paleo-Mesoarchean boundary

**DOI:** 10.1093/nsr/nwab136

**Published:** 2021-07-30

**Authors:** Xiaolei Wang, Ming Tang, Jeff Moyen, Di Wang, Alfred Kröner, Chris Hawkesworth, Xiaoping Xia, Hangqiang Xie, Carl Anhaeusser, Axel Hofmann, Junyong Li, Linsen Li

**Affiliations:** State Key Laboratory for Mineral Deposits Research, School of Earth Sciences and Engineering, Nanjing University, Nanjing 210023, China; School of Earth and Space Sciences, Peking University, Beijing 100871, China; Laboratoire Magmas et Volcans UMR6524, Université de Lyon, UJM-UCA-CNRS-IRD, Saint Etienne 42023,France; State Key Laboratory for Mineral Deposits Research, School of Earth Sciences and Engineering, Nanjing University, Nanjing 210023, China; Institut für Geowissenschaften, Universität Mainz, Mainz 55099, Germany; Department of Earth Sciences, University of Bristol, Bristol BS8 1RJ, UK; State Key Laboratory of Isotope Geochemistry, Guangzhou Institute of Geochemistry, Chinese Academy of Sciences, Guangzhou 510640, China; SHRIMP Center, Institute of Geology, Chinese Academy of Geological Sciences, Beijing 100037, China; Economic Geology Research Unit, University of the Witwatersrand, Johannesburg 2050, South Africa; Department of Geology, University of Johannesburg, Johannesburg 2006, South Africa; State Key Laboratory for Mineral Deposits Research, School of Earth Sciences and Engineering, Nanjing University, Nanjing 210023, China; State Key Laboratory for Mineral Deposits Research, School of Earth Sciences and Engineering, Nanjing University, Nanjing 210023, China

**Keywords:** zircon oxygen isotopes, TTG, plate tectonics, deep recycling, Paleo-Mesoarchean boundary

## Abstract

The recycling of supracrustal materials, and in particular hydrated rocks, has a profound impact on mantle composition and thus on the formation of continental crust, because water modifies the physical properties of lithological systems and the mechanisms of partial melting and fractional fractionation. On the modern Earth, plate tectonics offers an efficient mechanism for mass transport from the Earth's surface to its interior, but how far this mechanism dates back in the Earth's history is still uncertain. Here, we use zircon oxygen (O) isotopes to track recycling of supracrustal materials into the magma sources of early Archean igneous suites from the Kaapvaal Craton, southern Africa. The mean *δ*^18^O values of zircon from TTG (tonalite–trondhjemite–granodiorite) rocks abruptly increase at the Paleo-Mesoarchean boundary (ca. 3230 million years ago; Ma), from mantle zircon values of 5‰–6‰ to approaching 7.1‰, and this increase occurs in ≤3230 Ma rocks with elevated Dy/Yb ratios. The ^18^O enrichment is a unique signature of low-temperature water–rock interaction on the Earth's surface. Because the later phase was emplaced into the same crustal level as the older one and TTG magmas would derive from melting processes in the garnet stability field (>40 km depth), we suggest that this evident shift in TTG zircon O isotopic compositions records the onset of recycling of the mafic oceanic crust that underwent seawater hydrothermal alteration at low temperature. The onset of the enhanced recycling of supracrustal materials may also have developed elsewhere in other Archean cratons and reflects a significant change in the tectonic realm during craton formation and stabilization, which may be important processes for the operation of plate tectonics on early Earth.

## Teaser

Deep recycling of supracrustal materials and possible operation of plate tectonics started at ca. 3230 million years ago, revealed by zircon oxygen isotopes in southern Africa.

## INTRODUCTION

A unique feature of Earth's continental crust, in comparison to other rocky planets in the solar system, is its felsic bulk composition. A key process in continental formation is the operation of plate tectonics and the progressive reworking of increasingly silicon-rich lithologies, from ultramafic, through mafic, to felsic. Radiogenic and stable isotope systems have been widely used to fingerprint the evolution of crustal components during their geochemical differentiation [1–3], but stable isotopes, particularly oxygen (O) isotopes in zircon, are uniquely powerful in tracing contributions from supracrustal materials including sedimentary rocks and altered igneous rocks.

The O isotope composition of most mantle peridotites spans a narrow range (+5.5 ± 0.2‰ in *δ*^18^O) [4,5], but it varies widely (>30‰) in crustal rocks due to pervasive water–rock interaction on the Earth's surface [6]. In general, low temperature hydrothermal alteration on the Earth's surface leads to ^18^O enrichment in altered igneous rocks [5], and this is observed back into the early Archean [7,8]. Recycling of such altered materials into magma sources results in heavy O isotope signatures for magmatic minerals. However, the timing and mechanism for the recycling are highly debated [9], casting doubt on the styles of plate tectonics on early Earth.

Here, we examine O isotopes in zircon grains from tonalite–trondhjemite–granodiorite (TTG), the major felsic lithology of the Archean continental crust, to evaluate the recycling of supracrustal materials in the Archean. The Kaapvaal Craton and specifically the Barberton granitoid-greenstone terrain (BGGT) provide robust rock-based benchmarks for early Earth studies because of exceptional preservation of early Archean TTG and greenstone rocks. These TTGs were mainly emplaced into the same Onverwacht Group in three stages: at ca. 3510 Ma, 3450 Ma and 3280–3220 Ma [10], and they mostly preserve primary igneous features and intrusive relations with their country rocks [10,11]. Following regional deformation and metamorphism at ca. 3230 Ma [12], post-subduction plutonic rocks are more potassic, ranging from granodiorite to granite and syenite, as seen in the composite batholiths

of the GMS (granite-monzogranite-syenite) suite [13,14].

**Figure 1.**
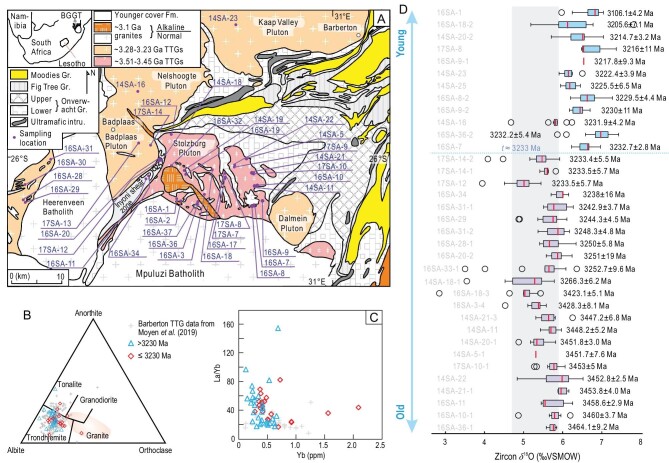
(A) Simplified geological map of the Barberton granitoid-greenstone terrain (BGGT) to the east and northeast of Badplaas (modified from Anhaeusser and Robb [11]). (B) Ab-Or-An diagram showing the classification of the BGGT granitoid samples; the shaded area shows the composition of modern continental crust [57]. One Nederland sample (red diamond) plots in the granite area of this diagram. (C) La/Yb-Yb diagram showing the high La/Yb ratios and low Yb contents of the BGGT TTGs. (D) Box and whisker plots of zircon *δ*^18^O values of BGGT TTGs from this study. The location of each sample can be found in Fig. 1A and Supplementary Table S1. Each box represents multiple spot analyses of zircon from one sample, with the sample name shown on the left side and mean age (Ma) on the right side. The bottoms and tops of boxes are 25th and 75th percentiles, respectively, and the lower and upper whiskers denote 2.5th and 97.5th percentiles, respectively. The outliers are shown by the open circles.

The present study investigates the geochemistry of 51 samples from 12 granitoid intrusions from the BGGT (Fig. 1A) and reports *in situ* secondary ion mass spectroscopy (SIMS) U-Pb and oxygen isotope data for zircon grains from 34 of the rocks studied (Supplementary Table S1). The ages of the undated samples are constrained by the ages of adjacent samples in the field. Most of the samples are from southeast of Badplaas, including the majority of the TTGs that occur within or around the BGGT (Fig. 1A). Careful evaluations of internal texture, calculations of alpha dose and laser Raman analyses were undertaken on the analyzed zircon grains to avoid secondary overprints (radiation damage, micro inclusions, cracks, etc.) on the sites selected for oxygen isotope analysis. The results provide important insights into the origin of TTGs in the Archean.

## RESULTS

The samples are predominantly trondhjemite, with rarer tonalite and granodiorite (Fig. 1B). They show typical features of TTGs, such as steep REE (rare earth element) patterns, high concentrations of Sr (239–1089 ppm, average 525 ppm) and Al_2_O_3_, and low concentrations of Y (1.7–21 ppm, average 6.1 ppm) and Yb (0.12–1.56 ppm, average 0.46 ppm), resulting in high Sr/Y (33–249, average 99) and La/Yb (16–154, average 44) ratios (Fig. 1C; Table S2). Zircon SIMS U-Pb dating yields crystallization ages of 3465 Ma to 3205 Ma, consistent with published data [10], and the syenites that intruded the TTG plutons crystallized at ca. 3100 (Fig. 1D). Our SIMS zircon U-Pb dating results show different degrees of recent Pb loss for some samples, but the analyses with concordia ages yield consistent weighted mean ^207^Pb/^206^Pb ages for samples from the same plutons/intrusions (Fig. S1 and Table S3).

**Figure 2.**
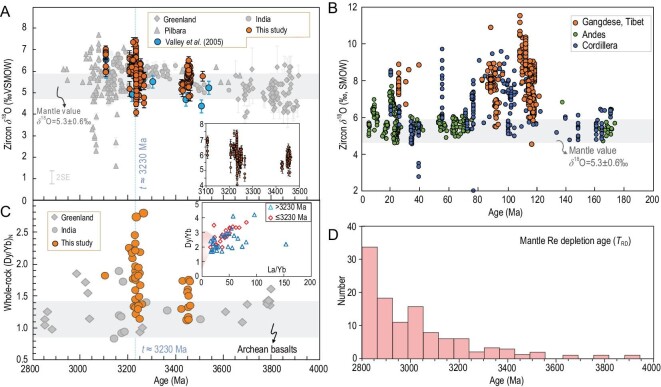
(A) *δ*^18^O versus age diagram showing the distribution of available zircon *δ*^18^O of TTGs from this work and literatures. The blue dashed line in Fig. 2A marks *t *= ca. 3230 Ma and cuts the analyses into two parts. Samples younger than this age show departures from mantle oxygen isotope compositions. The data of Greenland, Pilbara and India are from refs [6,18–20,58]. The inset shows the detailed distribution of the analyses with time in this work. (B) Compilation of zircon *δ*^18^O values from Phanerozoic continental arc regions. The data on the Phanerozoic continental arc are from refs [59–65]. The significant increase of zircon *δ*^18^O since ca. 120 Ma indicates the input of supracrustal materials (mainly sediments) to the source of these granitoids. The gray band in Fig. 2A and B represents the values of mantle zircon (5.3 ± 0.6‰) [6]. (C) Whole-rock Dy/Yb versus age diagram. (Dy/Yb)_N_ is normalized to chondrite (data from Sun and McDoungh [66]). The gray band in Fig. 2C represents the (Dy/Yb)_N_ of Archean basalts (1.14 ± 0.28; 1std; calculated from the dataset of Tang *et al.* [37]). The inset in Fig. 2C highlights that the high La/Yb and Dy/Yb ratios of the investigated Barberton TTGs are different from the published Archean basalts. The samples in Fig. 2A and C are plotted with their weighted mean ages. (D) Distribution of Re depletion model ages (*T*_RD_, minimum ages of melt withdrawal from mantle) of mantle sulfides, alloy grains and peridotites (data and references compiled in Table S5 and supplementary appendix) showing a mild increase in frequency at the Paleo-Mesoarchean boundary.

The U-Pb isotopic dating was done in the same domains analyzed previously for O isotope ratios, but with a bigger beam size, and only the O isotope data of spot analyses with concordant U-Pb ages were used for this study. Zircon *δ*^18^O values of the TTG rocks in the Barberton area show an overall increase in terms of ranges and averages at ca. 3233 Ma (for simplicity and dating uncertainty, we define it at ca. 3230 Ma) close to the Paleoarchean-Mesoarchean boundary (Fig. 1D). Although there might be minor intrasample variations, the >3230 Ma TTGs have zircon *δ*^18^O values mostly in the range of mantle zircon with averages from +5.07‰ to +6.02‰, whereas TTGs of ≤3230 Ma have mildly elevated zircon *δ*^18^O values with the largest value close to ∼8‰ (averages from 5.95‰ to 7.08‰ for the different samples) (Figs 1D and 2A; Table S4). To evaluate the effect of radiation damage on the oxygen isotope results in zircon, we calculated the alpha dose and carried out laser Raman analyses on zircon following the procedures of Wang *et al.* [15] and Gao *et al.* [16]. There are no evident correlations of alpha dose and laser Raman results versus *δ*^18^O as there are in Wang *et al.* [15] and there is no difference in oxygen isotopes for zircon with *D*_α_^T^ lower than 6 × 10^15^ α-decay events/mg for each sample. Therefore, we adopt a cut-off of *D*_α_^T ^= 6 × 10^15^ events/mg when plotting the analyses in Fig. 2A which, although it does not change the tendency of oxygen isotope change from the older to the younger TTGs in BGGT, filtered some anomalous spot analyses as shown in Fig. 1D. No spatial variation in zircon *δ*^18^O values is observed for different phases of the TTG intrusions. There are sporadic analyses showing lower *δ*^18^O values than mantle zircon values of 5.3 ± 0.6‰ [6] throughout the Archean era (Fig. 2A). This may be caused by either the incorporation of high-temperature seawater-hydrothermally altered oceanic crust [4,17] or the weak effect of radiation damage [15,16], which could be common in the TTG formation of the whole Archean era.

## DISCUSSION

The increase in the average zircon *δ*^18^O values since the Paleoarchean-Mesoarchean boundary cannot be explained by crustal assimilation because: (i) no spatial variation is observed in the ≤3230 Ma TTGs; (ii) some of the younger intrusions were emplaced at the same crustal level as the older intrusive phases (such as the Stolzburg and Weergevonden composite plutons) [10]; and (iii) there is no large variation of *δ*^18^O, with the maximum being only 7.08‰. Strong assimilation with high *δ*^18^O supracrustal materials, if it existed, would generate elevated *δ*^18^O in the >3230 Ma TTGs. However, this is not observed in this study. Therefore, the systematic increase of zircon *δ*^18^O values at ca. 3230 Ma truly reflect the increase of heavy O isotopes in the later TTG magmas in the BGGT.

The O isotope increase is consistent with the few analyses of Barberton TTGs previously reported by Valley *et al.* [6], but they did not give precise age constraints on the O isotope transition and the small dataset for this area was concealed by plenty of Archean detrital zircon data worldwide. Similarly, this oxygen isotope increase is also reported in the Archean igneous rocks of other major Archean cratons including Greenland [18], Pilbara [19] and southern India [20] (Fig. 2A), but their sporadic data at their supposed boundaries are not enough to place a solid constraint with precise geochronology. Recently, a similar mild elevation of zircon *δ*^18^O was reported in Archean granitoids from the Pilbara Craton and the transition was defined at ca. 2.9 Ga [9]. However, their classifications of the rock types are ambiguous, and they ignored the previously reported *δ*^18^O elevations at ca. 3.2 Ga and ca. 3.1 Ga in the same craton [19]. In contrast, our new data from the typical Kaapvaal Craton, combined with refined zircon U-Pb dating and whole-rock geochemistry, provide a robust constraint on the widespread increase of *δ*^18^O, possibly on a large part of the Earth. Moreover, this increase in maximum zircon *δ*^18^O is also consistent with the sudden decrease of average triple-oxygen-isotope values of shales in the Barberton area [21], although this decrease is more marked in post-Archean time.

Because ^18^O enrichment is a diagnostic signature of low temperature water–rock interaction on the Earth's surface [4,17], the nearly synchronous increase in the maximum zircon δ^18^O from global granitic rocks points to the onset of widespread reworking of supracrustal materials at crustal depths of >40 km at the Paleoarchean-Mesoarchean boundary. The supracrustal materials could be cherts and/or shales deposited in the Paleoarchean or earlier, which have relatively high *δ*^18^O values of 10‰–22‰ [22,23] and 6‰–11‰ [22], respectively. Alternatively, the mafic oceanic crust that had experienced low-temperature seawater hydrothermal alteration during basalt eruption along mid-ocean ridges may also acquire elevated *δ*^18^O values [6,9,23], and the incorporation of these high *δ*^18^O materials into the sources of the younger TTGs (≤3230 Ma) is possibly responsible for their elevated *δ*^18^O values. If we could take the average of 3.0 Ga shales with *δ*^18^O of 10‰ [22] as the endmember of supracrustal sediments, a simple mass balance calculation would yield ∼23% supracrustal sediments in the sources of the ≤3230 Ma TTGs when adopting the average zircon *δ*^18^O of 6.4‰ for the BGGT TTGs. The proportion would be over 37% when calculating using the highest *δ*^18^O of 7.08‰. Obviously, such amounts of supracrustal sediments in source would result in evident whole-rock geochemical changes than the older TTG phases, which, however, are not observed in this study. Therefore, we prefer the low-temperature altered mafic crust have played a very important role in the magma source of the ≤3230 Ma TTGs.

Similar increases of *δ*^18^O values are commonly seen in zircon grains of Phanerozoic continental arc magmas (Fig. 2B), where they are taken to suggest the existence of supracrustal rocks in the zones of melt generation after tectonic thickening through oceanic subduction. Although the incorporation of supracrustal materials could take place through crustal assimilation [24], melting of the supracrustal materials in magma source is the most effective way to yield elevated *δ*^18^O values. Thus, strong crustal assimilation can be excluded as aforementioned. It is noted that the increases of *δ*^18^O in Phanerozoic arc magmatic rocks can be higher (mostly close or over 9‰; Fig. 2B) than those of the Archean TTGs (Fig. 2A). This could result from the sharp increase of *δ*^18^O in sediments since the late Archean [22] at constant crustal silicon compositions since the early Archean [25]. The same amount of supracrustal rock with different *δ*^18^O values in the source at the Paleo-Mesoarchean transition may generate different O isotope compositions from those at the Phanerozoic.

For the BGGT TTG samples, most of them have strongly fractionated REE patterns, which are reflected in their high La/Yb and Dy/Yb ratios relative to Archean basalts. The ≤3230 Ma TTGs, especially, show elevated Dy/Yb ratios (Fig. 2C). These are taken to reflect residual garnet in their source regions during crustal melting, and together with the elevated zircon *δ*^18^O values, these observations suggest that the supracrustal materials start to exist in the garnet stability field (>40 km) at the Paleoarchean-Mesoarchean boundary. In Barberton, this recycling correlates with a shift towards higher Dy/Yb ratios (for a given La/Yb) (Fig. 2C), a signature of residual garnet during crustal melting (Fig. 2C inset), and it may also suggest significant crustal thickening at a time around 3.2 Ga. In contrast to the BGGT, the TTGs in other continents, although yielding similar increases in the maximum zircon *δ*^18^O at ca. 3.2 Ga, also show a large number of grains with mantle-like *δ*^18^O values at ≤3.2 Ga (Fig. 2A). This suggests the contemporaneous melting of crustal sources dominated by igneous materials although the melting of supracrustal materials had taken place in some parts of the early Earth.

Elevated *δ*^18^O values have also been observed in detrital zircon of the Eoarchean to Hadean ages, which were interpreted to reflect reworking of supracrustal materials as early as the Hadean [6,26–29]. However, detrital zircon lacks geological context [30–32]. Particularly, the depths to which supracrustal materials were positioned in the early Earth are yet to be established. If they were derived from volcanic rocks that erupted on the surface or emplaced at the shallow crustal level, the role of assimilation of supracrustal materials should be taken into account. Moreover, the significance of sporadically found high *δ*^18^O in zircon may be further complicated by complex metamorphism and alteration assisted by radiation damage [33]. Alternatively, the elevated *δ*^18^O in the Eoarchean to Hadean detrital zircon could be a combination of multiple recycling processes here and there, whereas the overall patterns conceal the specific tectonism in specific areas at a specific time. Combined with field geology, geochronology and intrasample comparison, our findings from the BGGT TTGs provide the first robust evidence for significant crustal recycling to depths of >40 km from ca. 3.2 Ga in ancient continents. This is consistent with the observation of mass-independently fractionated S isotopes, a unique signature of Archean surface-derived S, in diamonds younger than 3.0 Ga [34]. Moreover, it is also consistent with increased craton stabilization from ca. 3.2 Ga as indicated by the distribution of the Re depletion model ages (*T*_RD_) of mantle sulfides, alloy grains and peridotites (see the compiled data in Fig. 2D).

We now explore the possible mechanisms driving the recycling of supracrustal materials to depths of >40 km at the Paleoarchean-Mesoarchean transition. Plate tectonics can efficiently transport surface materials to the Earth's interior, but the timing and mechanism for the onset of plate tectonics are subject to extensive debate today [35,36]. Transition metal chemistry of terrigenous sediments and Rb/Sr systematics in igneous rocks suggests the existence of a largely mafic crust [37,38], whereas in consideration of effects such as the secular cooling of the Earth's mantle and the biologically driven oxidation of the Earth's atmosphere, the crustal silica content is suggested to have been constant since at least the early Archean [25]. In addition, Ti isotopes in terrigenous sediments have been taken to indicate the widespread operation of subduction processes as early as 3.5 Ga [39], and metamorphic records even point to the earliest start in the Eoarchean as featured by warm subduction [40].

The elevated zircon *δ*^18^O is a clear sign of the recycling of the supracrustal materials into the sources of the Mesoarchean TTGs [6], but the zircon *δ*^18^O values of the Mesoarchean TTGs depart from the mantle zircon value by only 1‰–2‰ (Fig. 2A) [24], which is in contrast with up to 6‰–7‰ in zircon from I-type granitoids above modern oceanic subduction zones (Fig. 2B). In addition, S-type granites, the products of metasediment melting, are largely absent in the Paleoarchean and scarce in the Mesoarchean [41–43]. We thus favor the theory that modern plate tectonics were not operating when these TTGs were formed at least at the Paleo-Mesoarchean transition. Instead, ancient plate tectonics were operating through warm subduction for lithospheric thickening along converging plate boundaries [40].

As argued above, the low-temperature altered mafic crust is preferable as the dominant source for ≤3230 Ma TTGs. Supracrustal materials on the Earth's surface can be transported to crustal depths of >40 km via subduction. Therefore, subduction is a feasible mechanism for burial of the supracrustal materials to crustal depths of >40 km. Because of the high mantle temperature in the Archean, the subducting oceanic crust would be susceptible to thickening during plate convergence [44]. As soon as the mafic oceanic crust was transformed to garnet granulite through regional metamorphism at moderate thermal gradients [40], gravitational delamination would take place along previously thickened boundaries [44]. This may induce active rifting for partial melting of the mafic oceanic crust, in which the high *δ*^18^O materials would be responsible for the high *δ*^18^O zircon in the resultant TTGs. Therefore, the deep recycling of the altered mafic oceanic crust can produce the TTG melts with mildly higher *δ*^18^O than the mantle zircon. This points to a two-stage mechanism for the origin of TTGs: the basaltic protolith generated at divergent plate boundaries and the felsic magma produced at convergent plate boundaries.

The shift in TTG zircon *δ*^18^O correlates with periods of major craton formation (Fig. 2A and D) [45,46]. Most cratonic peridotites show systematically higher metamorphic pressures relative to magmatic pressures, indicating significant thickening of the oceanic lithosphere and upward transport of peridotite-derived melts for mafic magmatism [47]. The garnet fractionation signatures seen in most Archean igneous differentiation suites may also imply the widespread operation of lithospheric thickening in the late Archean [48]. In addition, lithospheric rifting results in the extensional realm in the early Archean, which may drive the transformation of stagnant to mobile lid tectonics with both warm subduction and hot rifting along convergent and divergent plate boundaries, respectively [40].

Geodynamic modeling corroborates the operation of both compressional and extensional processes in the formation of Archean cratons [48–50]. Compressional tectonism is coupled with subduction for lithospheric thickening [48,51]. The extensional tectonism is associated with lithospheric rupture for the transition from the stagnant to mobile lid tectonics [49]. Whereas such processes were initiated in the Eoarchean, they would have continued from the Paleoarchean, through Mesoarchean, to Neoarchean [40]. This is also indicated by the ca. 3230 Ma metamorphism in the BGGT [12]. The supracrustal remelting at deep crust, as indicated by the increased *δ*^18^O values, combined with the new dating results, provides refined geochemical and geochronological evidence for the tectonic transition of the Kaapvaal Craton and some other ancient continents (like Greenland [18], Pilbara [19] and southern India [20]).

**Figure 3.**
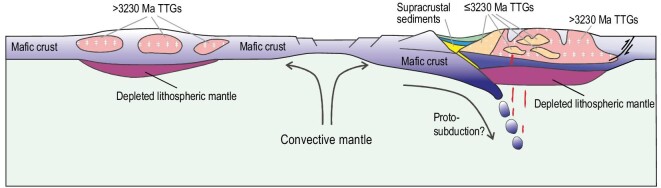
A simplified cartoon showing tectonic models of TTG formation in Paleoarchean and Mesoarchean eras as derived from data of the BGGT. The left part shows the >3230 Ma TTGs formed by melting of intracrustal mafic rocks with no involvement of supracrustal sediments or low-temperature altered basalts [57]. Compressional tectonics took place at ca. 3230 Ma and allowed recycling of supracrustal materials in deep crust due to crustal thickening or proto-subduction. The subsequent melting of supracrustal materials and mafic rocks within a partly thickened lithosphere generated the ≤3230 Ma TTGs with systematically elevated oxygen isotopes.

Increased erosion of high relief areas associated with regional metamorphism, episodic hot rifting and subsequent hydrothermal alteration, combined with the formation of large thrust faults, or proto-subduction (Fig. 3), is one pathway for transporting supracrustal materials to the deep crust. However, it is uncertain whether this increase was related to regional initiation of continental arc magmatism following the accretion of oceanic crust as envisaged by de Wit *et al.* [52]. The proto-subduction may be short-lived and episodic due to frequent slab breakoff in the hotter Archean mantle [53,54] (Fig. 3). Nevertheless, the supracrustal materials were carried into the deep crust and subsequently incorporated into the sources of TTGs (Fig. 3). In this regard, the global emergence of volatile influx from the Earth's surface to the Earth's interior would have promoted the development of subduction due to the lubricating effect of water at the slab interface [55,56]. These processes at ca. 3230 Ma may have been common in the Archean when the mantle temperature was sufficiently high to make the warm subduction for lithospheric thickening [40].

## MATERIALS AND METHODS

Samples were crushed to powder for major and trace element analyses. Whole-rock major element analyses of the samples were obtained using an ARL9800XP^+^ X-ray fluorescence spectrometer. Whole-rock trace elements were measured using a Finnigan Element II ICP-MS. The analytical precision for major and trace elements is generally better than 2% and 5%, respectively. Zircon grains were separated from samples (2–4 kg each) using conventional heavy liquid and magnetic techniques, cast in epoxy with standard zircon. Zircon mount is polished flat enough and to show their mid-sections. *In situ* U-Th-Pb and oxygen isotope analyses were guided by cathodoluminescence images and transmitted and reflected light photographs. Zircon oxygen isotopes were analyzed with the CAMECA IMS 1280-HR ion microprobe using multicollection mode. A focused beam of ∼10 μm diameter was rastered to make a ∼20 μm × 20 μm analyzed area. There is no through session drift on primary and secondary oxygen standards and thus no drift correction was applied. After oxygen isotope analysis, the zircon mounts were polished again to remove the oxygen isotope pits for further U-Pb analysis. Zircon grains of most samples were dated by a CAMECA IMS-1280HR ion microprobe and the others were analyzed using the SHRIMP II ion microprobe with a beam size of ∼30 μm. Measured U-Th-Pb compositions were corrected for common Pb using the ^204^Pb method. Uncertainties on individual analyses are reported at 1σ level, and mean ages for pooled ^206^^ ^Pb/^238^U results are quoted at 95% confidence level. Analytical details can be found in supplementary data.

## Supplementary Material

nwab136_Supplemental_File
