# Supplementary material for: The onset of deep recycling of supracrustal materials at the Paleo-Mesoarchean boundary
Source: Natl Sci Rev. 2021 Jul 30;9(3):nwab136. doi: 10.1093/nsr/nwab136 (PMC8900693; doi:10.1093/nsr/nwab136)
Supplement: nwab136_Supplemental_File [file nwab136_supplemental_file.pdf]

Supplementary files of the manuscript

submitted to NSR

The onset of deep recycling of supracrustal materials at the Paleo-Mesoarchean boundary

X. L. Wang[*1], J.-F. Moyen[2], D. Wang[1], A. Kröner[3], C. J. Hawkesworth[4], X. P. Xia[5], H. Q. Xie[6], C. R. Anhaeusser[7], A. Hofmann[8], J. Y. Li[1], L. S. Li[1]

[1]State Key Laboratory for Mineral Deposits Research, School of Earth Sciences and Engineering, Nanjing University, Nanjing 210023, China.
[2]Université de Lyon, Laboratoire Magmas et Volcans, UJM-UCA-CNRS-IRD, 23 rue Dr. Paul Michelon, 42023 Saint Etienne, France
[3]Institut für Geowissenschaften, Universität Mainz, 55099 Mainz, Germany
[4]Department of Earth Sciences, University of Bristol, Wills Memorial Building, Queens Road, Bristol BS8 1RJ, UK
[5]State Key Laboratory of Isotope Geochemistry, Guangzhou Institute of Geochemistry, Chinese Academy of Sciences, Guangzhou 510640, China
[6]SHRIMP Center, Institute of Geology, Chinese Academy of Geological Sciences, Beijing 100037, China
[7]Economic Geology Research Unit, University of the Witwatersrand, Johannesburg, South Africa
[8]Department of Geology, University of Johannesburg, Johannesburg, South Africa

Contents of the supplementary files

Detailed analytical methods.

References in the supplementary text and tables.

Fig. S1. Representative Cathodoluminescent images of analyzed zircon grains.

Fig. S2. SIMS zircon U-Pb Concordia plots for the BGGT TTGs.

Table S1 Sample information of the BGGT TTGs in South Africa.

Table S2 Major (wt%) and trace element (ppm) analyses of the BGGT TTGs in South Africa.

Table S3 SIMS zircon U-Pb isotope results for the BGGT TTGs in South Africa (Part I- by CAMECA).

Table S3 SIMS zircon U-Pb isotope results for the BGGT TTGs in South Africa (Part II- by SHRIMP).

Table S4 SIMS zircon in-situ oxygen isotope results for the BGGT TTGs in South Africa.

Table S5 Compiled Re model ages (Ga) for mantle sulfides, alloy grains and peridotites.

[*] Corresponding author. Email: wxl@nju.edu.cn

Detailed analytical methods

Whole-rock geochemical analysis

Samples were crushed to powder for major and trace element analyses. Whole-rock major analyses of the samples were obtained using an ARL9800XP+ X-ray fluorescence spectrometer (XRF) at the State Key Laboratory for Mineral Deposits Research, Nanjing University. The glass discs were prepared by fusion of a mixture with an alkali flux consisting of a mixture of lithium tetraborate, lithium metaborate and lithium bromide at 1050 ºC. Analyses were carried out with an accelerating voltage of 50 kV and a beam current of 50 mA. Standards BHVO-2, RGM-2 and BCR-2 were prepared using the same procedure to monitor the analytical accuracy. The analytical precision is generally better than 2% for all elements.

Whole-rock trace elements were measured at the same key laboratory of Nanjing University, using a Finnigan Element II ICP-MS following the procedures of Gao et al. (2003). The international standards BHVO-2, AGV-1, GSP-2 and BCR-2 were used for analytical quality control. The analytical precision for most trace elements is better than 5%, and the analytical results are listed in Table S2 of the supplementary file.

In situ zircon oxygen isotopes

Zircon grains were separated from samples (2-4 kg each) using conventional heavy liquid and magnetic techniques, cast in epoxy, and polished to their mid-sections. In situ U-Th-Pb isotope analyses were guided by cathodoluminescence (CL) images and transmitted and reflected light photographs. Zircon oxygen isotopes were analyzed with the CAMECA IMS 1280-HR ion microprobe at the Secondary Ion Mass Spectrometry Laboratory, Guangzhou Institute of Geochemistry, Chinese Academy of Sciences (GIG-CAS), with analytical procedures like those described by Li et al. (2013) and Li et al. (2010). The primary ion beam of $^{133}Cs^{+}$ was accelerated at 10 kV with an intensity of ~2 nA. A focused beam of ~10 µm diameter was rastered over 10 µm to make a ~20 × 20 µm analyzed area. The normal incidence electron flood gun was used to compensate for sample charging. Negative secondary ions were extracted with a 10 kV potential. Oxygen isotopes were measured using multicollection mode. Oxygen isotope ratios

were expressed in the standard $\delta^{18}O$ notation, signifying deviation of the measured $^{18}O/^{16}O$ value from the Vienna standard mean ocean water (VSMOW: $^{18}O/^{16}O$=0.0020052) (Baertschi et al., 1976) in parts per thousand. Previous studies have shown that the Dynamical Transfer (DT) lens centering values would show a positive correlation with oxygen isotopic composition when the sample is not topographic flat, which will result of bad precision (Kita et al., 2009; Tang et al., 2015). Such correlations were not found in our zircon grains that have been polished flat enough and have high precisions, which mean the DT lens centering values have not affected our oxygen isotope analyses. The Penglai zircon standard was used as an external standard, and it had a weighted mean $\delta^{18}O$ value of 5.31 ± 0.10 ‰ (2σ) (Li et al., 2010). The Qinghu zircon standard was selected as the other external standard to monitor the analytical precision during the course of this study, and it gave a mean $\delta^{18}O$ value of 5.50 ± 0.068 ‰ (recommended value: 5.4 ± 0.20 ‰, 2SD) (Li et al., 2013). There is no through session drift on primary and secondary oxygen standards and thus no drift correction was applied. The zircon O isotopic compositions from this study are listed in the supplementary Table S4.

In situ zircon U-Pb dating

After oxygen isotope analysis, the zircon mounts were polished again to remove the oxygen isotope pits for further U-Pb analysis. Zircon grains of most samples were dated by a CAMECA IMS-1280HR ion microprobe with procedures similar to those of Yang et al. (Yang et al., 2015). The zircon standard Plesovice was used for an external standard with a recommended $^{206}Pb/^{238}U$ age of 337.13 ± 0.37 Ma (Sláma et al., 2008). The zircon standard Qinghu with a recommended $^{206}Pb/^{238}U$ age of 159.5 ± 0.2 Ma (Li et al., 2013) was used for U-Pb isotopic fractionation calibration. Zircon U–Pb isotopic compositions for a few samples were analyzed using the SHRIMP II ion microprobe at the Beijing SHRIMP Center of China Academy of Geological Sciences under standard operating conditions (5-scan cycle, 2 nA primary O^{2-} beam, beam size of about 30 μm, mass resolution of 5000). U–Th–Pb isotopic ratios were determined relative to the

TEMORA-2 standard zircon with $^{206}Pb/^{238}U$=0.0668 corresponding to 417 Ma (Black et al., 2003), and the absolute abundances were calibrated to the standard zircon SL13. Analyses of the TEMORA-2 were interspersed with those of unknowns, following operating and data processing procedures like those described by Williams (Williams, 1998). The long-term external error for single U-Pb and Pb-Pb analysis of the lab is better than 1.5% and 1% for Plesovice standard and the internal errors of $^{207}Pb/^{206}Pb$ ratios of this work are mainly better than 0.5%. Measured compositions were corrected for common Pb using the ^{204}Pb method. Uncertainties on individual analyses are reported at 1σ level, and mean ages for pooled $^{206}Pb/^{238}U$ results are quoted at 95% confidence level. The U-Pb dating results are listed in the supplementary Table S3.

References in the supplementary methods and tables:

67. Gao JF, Lu JJ and Lai MY *et al.* Analysis of trace elements in rock samples using HR-ICPMS. *J. Nanjing Univ.* 2003; **39:** 844–850 (in Chinese with English Abs.).
68. Li XH, Tang GQ and Gong B *et al.* Qinghu zircon: a working reference for microbeam analysis of U-Pb age and Hf and O isotopes. *Chinese Sci. Bull.* 2013; **58**: 4647–4654.
69. Li XH, Long WG and Li QL *et al.* Penglai zircon megacrysts: a potential new working feference material for microbeam determination of Hf-O isotopes and U-Pb age. *Geostand. Geoanaly. Res.* 2010; **34**: 117–134.
70. Baertschi P. Absolute ^{18}O content of standard mean ocean water. *Earth Planet. Sci. Lett.* 1976; **31**: 341–344.
71. Kita NT, Ushikubo T and Fu B *et al.* High precision SIMS oxygen isotope analysis and the effect of sample topography. *Chemical Geology* 2009; **264:** 43–57.
72. Tang GQ, Li XH and Li QL *et al.* Deciphering the physical mechanism of the topography effect for oxygen isotope measurements using a Cameca IMS-1280 SIMS. *J. Anal. At. Spectrom.* 2015: **30**: 950.
73. Yang W, Hu S and Zhang JC *et al.* NanoSIMS analytical technique and its applications in earth science. *Sci. China: Earth Sci.* 2015; **58:** 1758–1767.
74. Sláma J, Košler J and Condon DJ *et al.* Plesovice zircon: a new natural reference material for U-Pb and Hf isotopic microanalysis. *Chem. Geol.* 2008; **249:** 1–35.
75. Black LP, Kamo SL and Williams IS *et al.* The application of SHRIMP to Phanerozoic geochronology: a critical appraisal of four zircon standards. *Chem. Geol.* 2003; **200:** 171–188.
76. Williams IS. U-Th-Pb geochronology by ion microprobe. In: McKibben MA, Shanks WC and Ridley WI (eds), Applications of microanalytical techniques to understanding mineralizing processes. *Rev. Econ. Geol.* 1998; **7:** 1–35.
77. Aulbach S, Griffin WL and Pearson NJ *et al.* Mantle formation and evolution, Slave Craton: constraints from HSE abundances and Re-Os isotope systematics of sulfide inclusions in mantle xenocrysts. *Chem. Geol.* 2004; **208:** 61–88.

78. Bernstein S, Hanghøj K and Kelemen PB *et al.* Ultra-depleted, shallow cratonic mantle beneath West Greenland: dunitic xenoliths from Ubekendt Ejland. *Contrib. Mineral. Petrol.* 2006; **152:** 335–347.
79. Carlson RW and Irving AJ. Re-Os systematics of lithospheric peridotites: implications for lithosphere formation and preservation. In: *Proceedings of the 7th International Kimberlite Conference* (eds Gurney JJ and Richardson SR) 1 (Cape Town 1999).
80. Carlson RW and Irving AJ. Depletion and enrichment history of subcontinental lithospheric mantle: an Os, Sr, Nd and Pb isotopic study of ultramafic xenoliths from the northwestern Wyoming Craton. *Earth Planet. Sci. Lett.* 1994; **126:** 457–472.
81. Carlson RW and Moore RO. Age of the Eastern Kaapvaal mantle: Re-Os isotope data for peridotite xenoliths from the Monastery kimberlite. *S. Afr. J Geol.* 2004; **107:** 81–90.
82. Chesley JT, Rudnick RL and Lee CT. Re-Os systematics of mantle xenoliths from the East African Rift: Age, structure, and history of the Tanzanian craton. *Geochim. Cosmochim. Acta* 1999; **63:** 1203–1217.
83. Gao S, Rudnick RL and Carlson RW. Re-Os evidence for replacement of ancient mantle lithosphere beneath the North China craton. *Earth Planet. Sci. Lett.* 2002; **198:** 307–322.
84. Griffin WL, Spetsius ZV and Pearson NJ *et al.* In situ Re-Os analysis of sulfide inclusions in kimberlitic olivine: New constraints on depletion events in the Siberian lithospheric mantle. *Geochem. Geophys. Geosys.* 2002; **3:** 1–25.
85. Hanghøj K, Kelemen P and Bernstein S *et al.* Osmium isotopes in the Wiedemann Fjord mantle xenoliths: a unique record of cratonic mantle formation by melt depletion in the Archaean. *Geochem. Geophys. Geosys.* 2001; **2:** 2000GC000085.
86. Irvine GJ, Pearson DG and Kjarsgaard BA *et al.* A Re-Os isotope and PGE study of kimberlite-derived peridotite xenoliths from Somerset Island and a comparison to the Slave and Kaapvaal cratons. *Lithos* 2003; **71:** 461–488.
87. Kröner A, Anhaeusser CR and Hoffmann JE *et al.* Chronology of the oldest supracrustal sequences in the Palaeoarchaean Barberton Greenstone Belt, South Africa and Swaziland. *Precambrian Res.* 2016; **279:** 123–143.
88. Menzies AH, Carlson RW and Shirey SB *et al.* Re-Os systematics of Newlands peridotite xenoliths: implications for diamond and lithosphere formation. In: *Proceedings of the 7th International Kimberlite Conference* (eds Gurney JJ and Richardson SR) 566 (Cape Town 1999).
89. Moyen JF and Martin H. Forty years of TTG research. *Lithos* 2012; **148:** 312–336.
90. Pearson DG, Snyder GA and Shirey SB *et al.* Archaean Re-Os age for Siberian eclogites and constraints on Archaean tectonics. *Nature* 1995c; **374:** 711–713.
91. Pearson DG, Shirey SB and Carlson RW *et al.* Re-Os, Sm-Nd, and Rb-Sr isotope evidence for thick Archaean lithospheric mantle beneath the Siberian craton modified by multistage metasomatism. *Geochim. Cosmochim. Acta* 1995b; **59:** 959–977.
92. Pearson DG, Irvine GJ and Carlson RW *et al.* The development of lithospheric keels beneath the earliest continents: time constraints using PGE and Re-Os isotope systematics. *Geol. Soc. London Special Pub.* 2002; **199:** 65–90.
93. Pearson DG, Carlson RW and Shirey SB *et al.* Stabilisation of Archaean lithospheric mantle: A Re-Os isotope study of peridotite xenoliths from the Kaapvaal craton. *Earth Planet. Sci. Lett.* 1995a; **134:** 341–357.

94. Richardson SH, Shirey SB and Harris JW *et al.* Archean subduction recorded by Re-Os isotopes in eclogitic sulfide inclusions in Kimberley diamonds. *Earth Planet. Sci. Lett.* 2001; **191:** 257–266.
95. Simon NS, Carlson RW and Pearson DG *et al.* The origin and evolution of the Kaapvaal cratonic lithospheric mantle. *J. Petrol.* 2007; **48:** 589–625.
96. Walker RJ, Carlson RW and Shirey SB *et al.* Os, Sr, Nd, and Pb isotope systematics of southern African peridotite xenoliths: implications for the chemical evolution of subcontinental mantle. *Geochim. Cosmochim. Acta* 1989; **53:** 1583–1595.
97. Wang KL, O'Reilly SY and Griffin WL *et al.* Proterozoic mantle lithosphere beneath the extended margin of the South China block: In situ Re-Os evidence. *Geology* 2003; **31:** 709–712.
98. Westerlund KJ, Shirey SB and Richardson SH *et al.* A subduction wedge origin for Paleoarchean peridotitic diamonds and harzburgites from the Panda kimberlite, Slave craton: evidence from Re-Os isotope systematics. *Contrib. Mineral. Petrol.* 2006; **152:** 275–294.
99. Wittig N, Webb M and Pearson DG *et al.* Formation of the North Atlantic Craton: timing and mechanisms constrained from Re-Os isotope and PGE data of peridotite xenoliths from SW Greenland. *Chem. Geol.* 2010; **276:** 166–187.

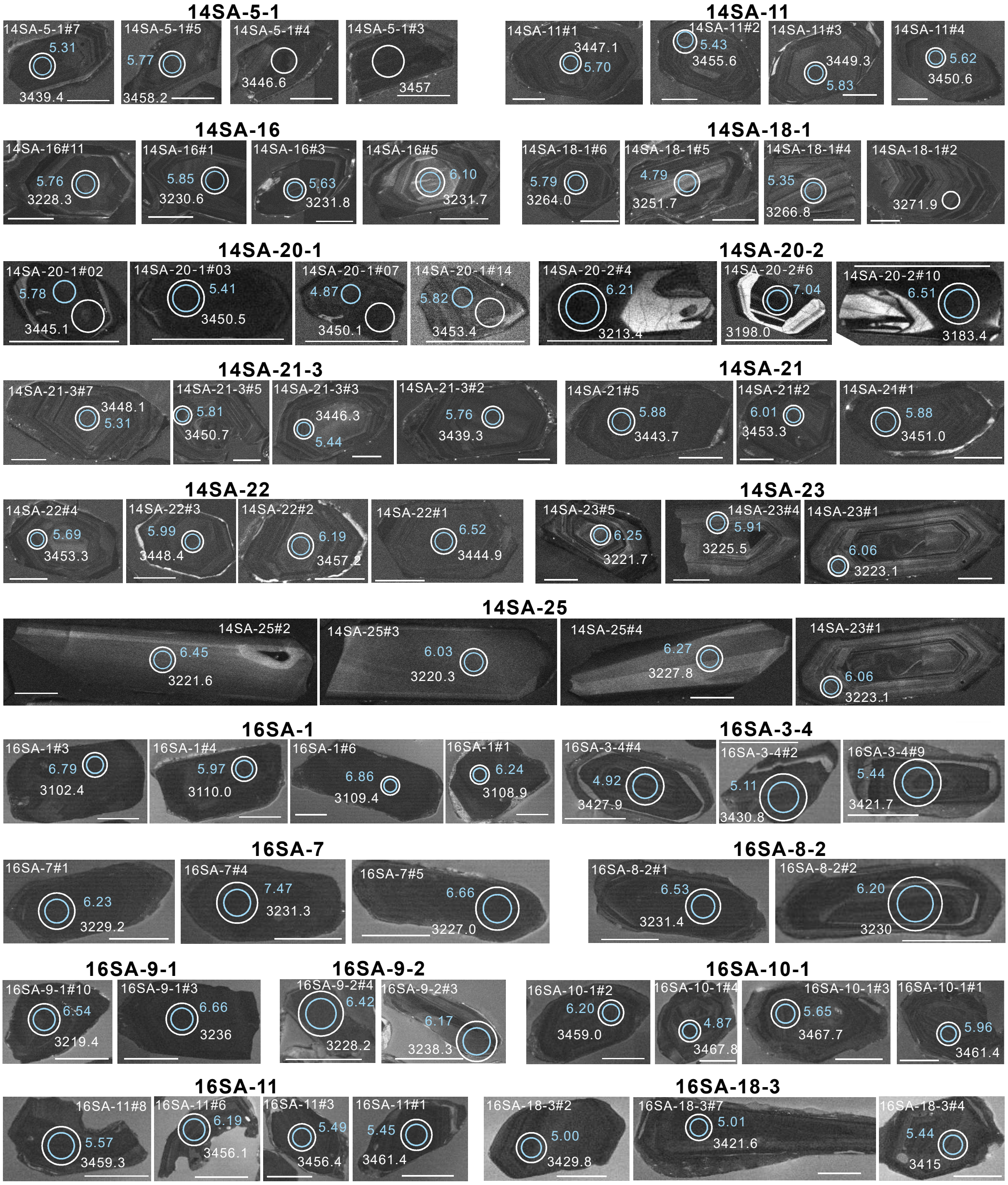

Fig. S1. Representative cathodoluminescent (CL) images of analyzed zircon grains. The white-color scale bar is 100 μm. The blue circles show positions of oxygen isotopes with $\delta^{18}O$ values shown nearby in blue color. The while solid circles indicate the positions of U-Pb analyses with $^{207}Pb/^{206}Pb$ dates shown nearby.

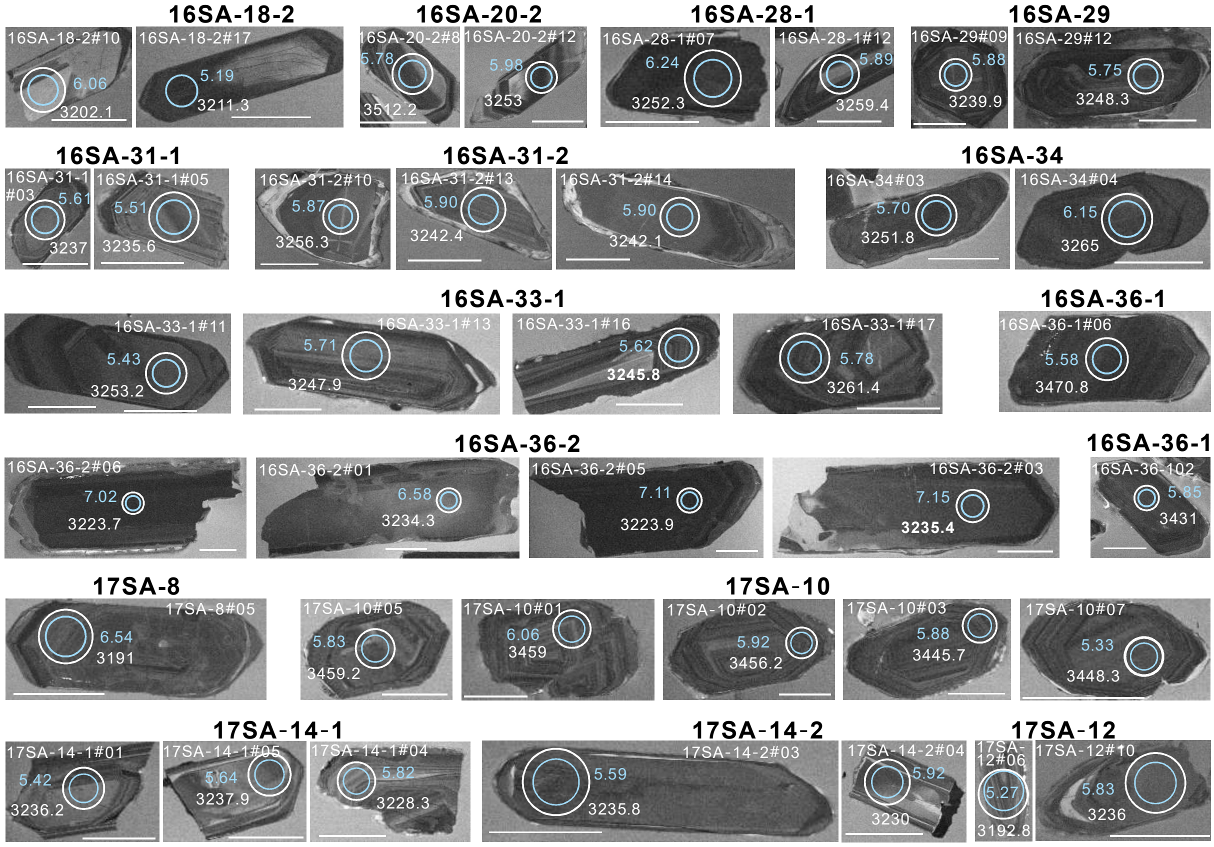

Fig. S1. (continued)

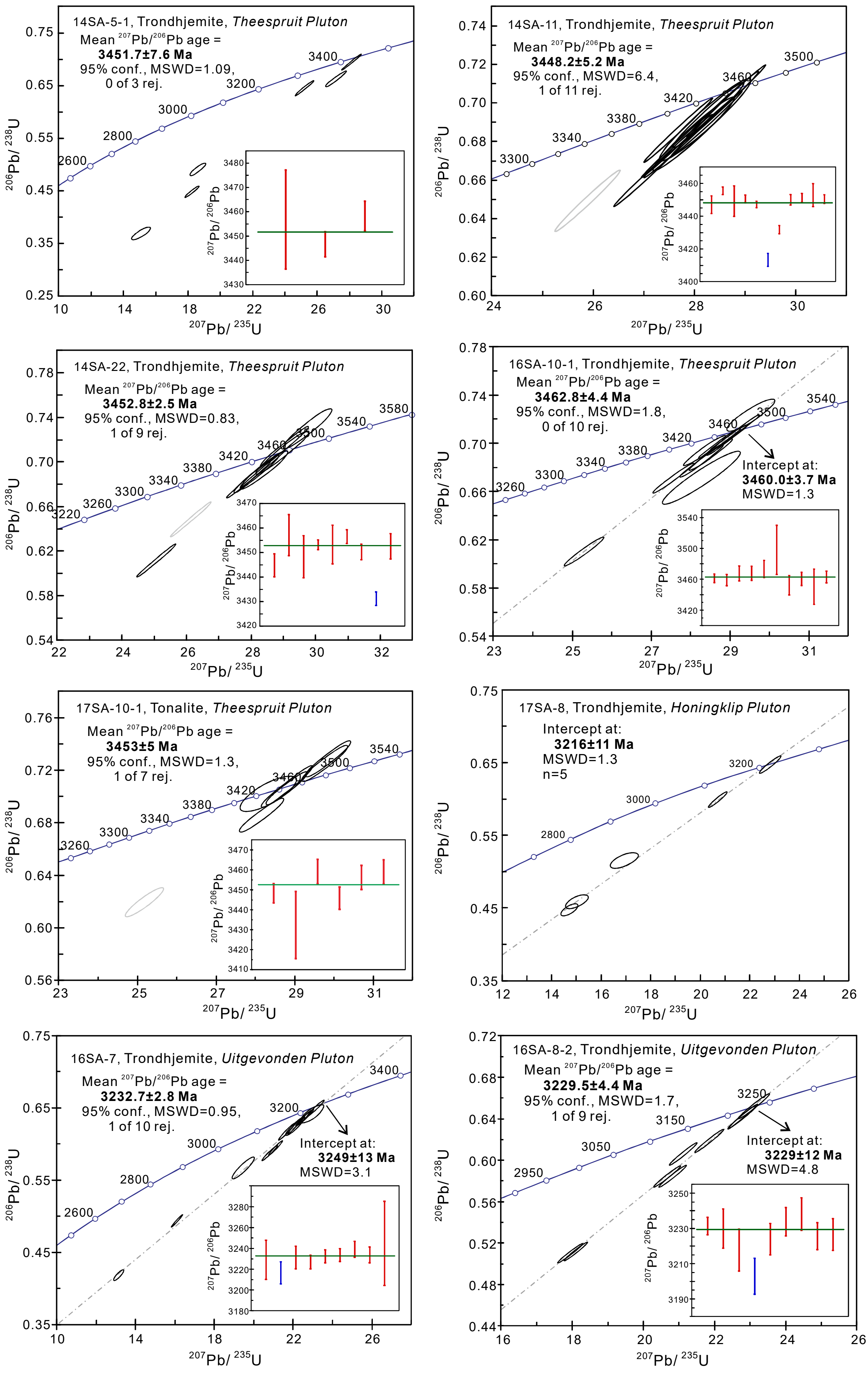

Supplementary Fig. S2. SIMS Zircon U-Pb Concordia plots for the BGGT TTGs

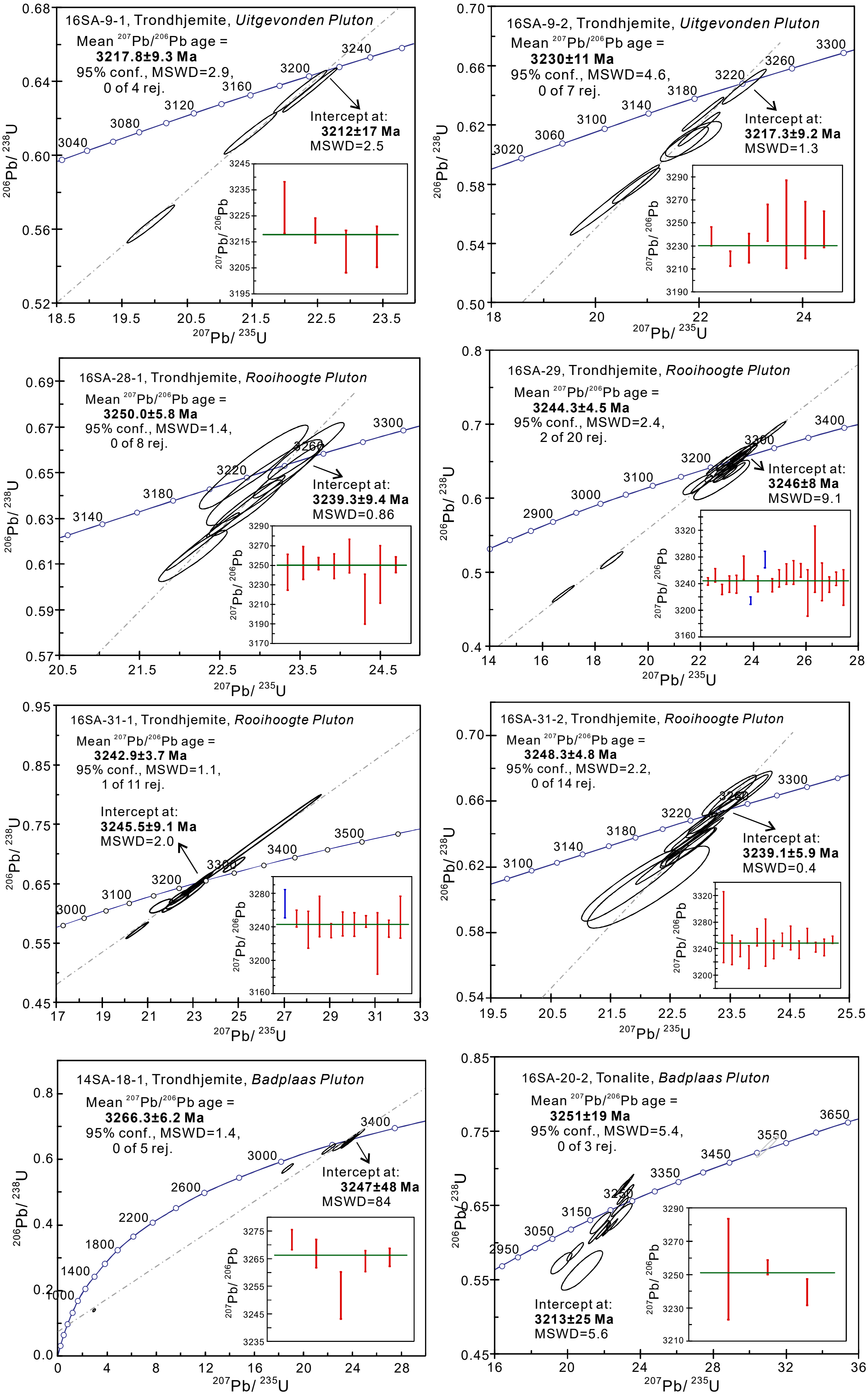

Fig. S2 (continued-1)

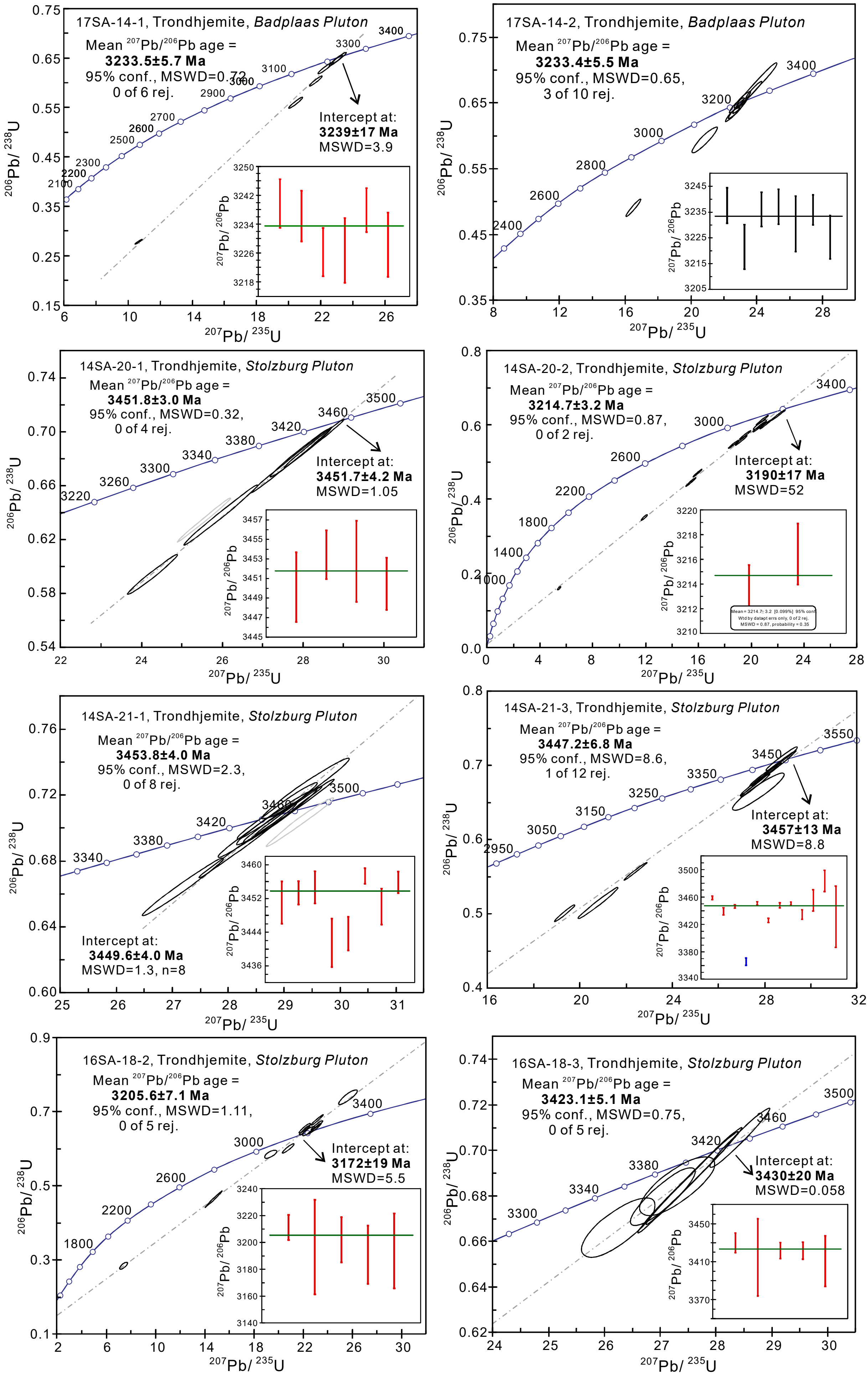

Fig. S2 (continued-2)

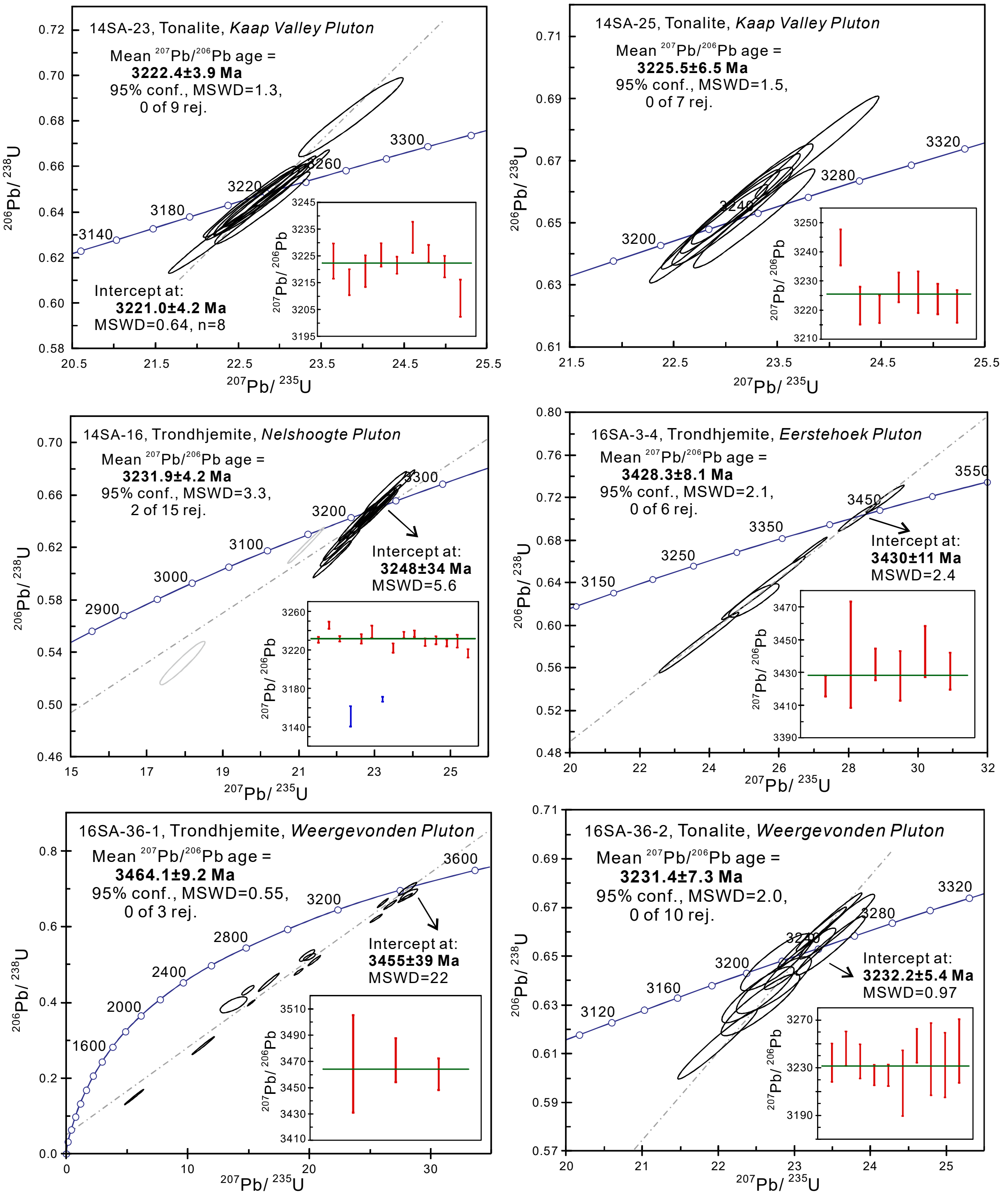

Fig. S2 (continued-3)

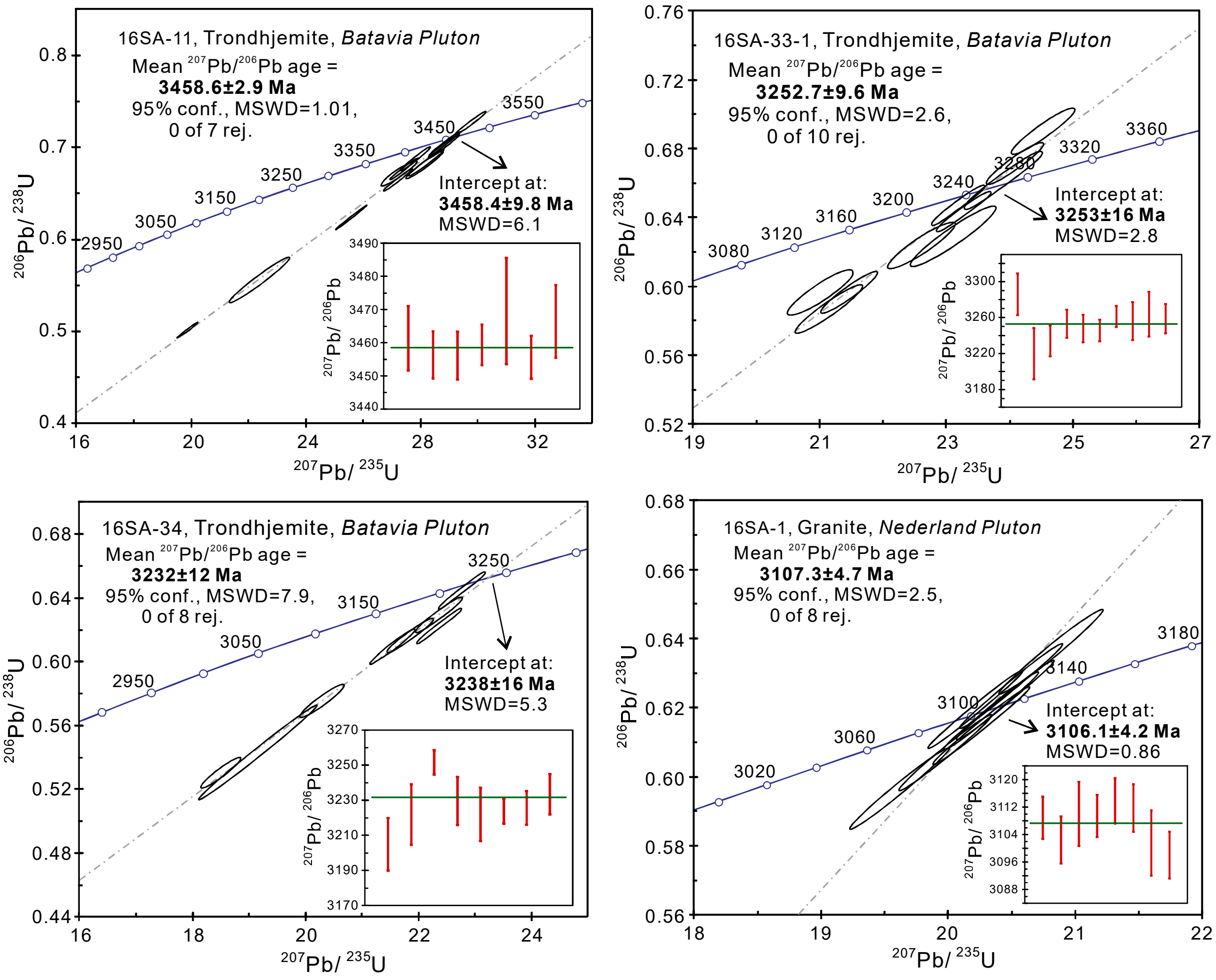

Fig. S2 (continued-4)

Supplementary Table S1 Sample information of the BGGT TTGs in South Africa

No.	Pluton / Intrusion	Sample	Rock type	Age (Ma)	GPS		Mineral assemblage
					Latitude (S)	Longitude (E)	
1	Badplaas	14SA-18-1	Trondhjemite	3266	26º00'25.42''	30º39'43.58''	Pl 45% + Qz 47% + Bt 8%
2		16SA-20-2	Tonalite	3251	26º00'25.79''	30º39'43.45''	Pl 58% + Qz 25% + Hb 17%
3		17SA-12	Trondhjemite	3235	26º00'32''	30º39'39''	Pl 47% + Qz 35% + Bt 15% + Kf 3%
4		17SA-13-1	Trondhjemite	3260	25º58'33''	30º36'58''	Pl 55% + Qz 33% + Bt 10% + Kf 2%
5		17SA-14-1	Trondhjemite	3234	25º58'55''	30º38'34''	Pl 55% + Qz 37% + Bt 8%
6		17SA-14-2	Trondhjemite	3233.4	25º58'55''	30º38'34''	Pl 50% + Qz 41% + Bt 9%
7	Batavia	16SA-11	Trondhjemite	3458.6	26º02'29.15''	30º38'14.49''	Pl 30% + Qz 50% + Kf 10% + Bt 10%
8		16SA-33-1	Trondhjemite	3252.7	26º02'27.37''	30º38'07.35''	Pl 50% + Qz 30% + Kf 10% + Bt 10%
9		16SA-33-2	Trondhjemite	3250	26º02'27.37''	30º38'07.35''	Pl 52% + Qz 28% + Kf 13% + Bt 7%
10		16SA-34	Trondhjemite	3238	26º02'15.63''	30º38'34.07''	Pl 55% + Qz 32% + Bt 10% + Kf 3%
11	Eerstehoek	16SA-3-4	Trondhjemite	3428.3	26º04'09.04''	30º44'58.49''	Pl 40% + Qz 35% + Bt 20% + Kf 5%
12	Honingklip	16SA-17	Trondhjemite	3220	26º02'11.07''	30º47'40.08''	Pl 20% + Qz 50% + Bt 15% + Kf 15%
13		17SA-7	Trondhjemite	3220	26º02'36''	30º47'23''	Pl 50% + Qz 35% + Bt 13% + Kf 2%
14		17SA-8	Trondhjemite	3216	26º02'32''	30º47'28''	Pl 45% + Qz 35% + Bt 17% + Kf 3%
15	Kaap Valley	14SA-23	Tonalite	3222	25º45'47.43''	30º48'44.09''	Pl 55% + Qz 30% + Bt 15%
16		14SA-25	Tonalite	3225.5	25º46'12.56''	31º03'57.37''	Pl 50% + Qz 35% + Bt 10% + Ep 5%
17	Nederland	16SA-1	Granite	3106.1	26º04'44.77''	30º41'30.12''	Kf 60% + Qz 30% + Bt 10%
18	Nelshoogte	14SA-16	Trondhjemite	3232	25º53'30.23''	30º37'19.85''	Pl 35% + Qz 55% + Bt 10%
19	Stolzburg	14SA-20-1	Trondhjemite	3451.8	26º01'30.78''	30º45'43.15''	Pl 35% + Qz 40% + Kf 15% + Bt 10%
20		14SA-20-2	Trondhjemite	3214.7	26º01'30.78''	30º45'43.15''	Pl 35% + Qz 55% + Bt 10%
21		14SA-21	Trondhjemite	3454	26º01'59.91''	30º50'23.64''	Pl 55% + Qz 38% + Bt 7%
22	Stolzburg	14SA-21-3	Trondhjemite	3447	26º01'57.88''	30º50'23.57''	Pl 50% + Qz 30% + Bt 20%
23		16SA-12	Trondhjemite	3450	25º57'46.19''	30º41'16.06''	Pl 55% + Qz 23% + Bt 13.5% + Kf 5.5% + Hb 3%
24		16SA-18-2	Trondhjemite	3205.6	26º01'27.35''	30º45'42.47''	Pl 30% + Qz 42%+ Bt 15% + Kf 8% + Hb 5% + minor Sph
25		16SA-18-3	Trondhjemite	3423.1	26º01'27.35''	30º45'42.47''	Pl 40% + Qz 40% + Kf 15% + Bt 5%
26	Theespruit	14SA-5-1	Trondhjemite	3452	26º00'06.88"	30º49'58.43"	Pl 40% + Qz 35% + Bt 15%
27		14SA-11	Trondhjemite	3448	26º03'19.52''	30º50'44.14''	Pl 30% + Qz 45% + Bt 10% + Kf 5% + Aln 10%
28		14SA-22	Trondhjemite	3453	26º01'15.77''	30º48'34.09''	Pl 40% + Qz 40% + Bt 15% + Kf 5%
29		16SA-10-1	Trondhjemite	3460	26º03'20.79''	30º51'01.53''	Pl 30% + Qz 37% + Kf 15% + Bt 10% + Hb 7% + Ep 1% + minor Sph
30		17SA-9-1	Tonalite	3450	26º01'16''	30º48'34''	Pl 50% + Qz 35% + Bt 15%
31		17SA-10-1	Tonalite	3453	26º01'60''	30º50'23''	Pl 55% + Qz 22% + Hb 15% + Bt 8% + minor Sph
32	Uitgevonden	16SA-4-1	Trondhjemite	3232	26º04'08.47''	30º50'46.47''	Pl 50% + Qz 26% + Bt 9% + Kf 8% + Hb 7%
33		16SA-4-2	Trondhjemite	3232	26º04'08.47''	30º50'46.47''	Pl 38% + Qz 50 % + Bt 6% + Hb 4% + Kf 2%
34		16SA-4-3	Trondhjemite	3232	26º04'08.47''	30º50'46.47''	Pl 35% + Qz 50% + Hb 7% + Bt 5% + Kf 3%
35		16SA-5-1	Trondhjemite	3232	26º04'57.96''	30º51'09.39''	Pl 45% + Qz 35% + Bt 15% + Kf 5%
36		16SA-6-1	Trondhjemite	3232	26º04'51.49''	30º51'14.78''	Pl 53% + Qz 22% + Kf 18% + Bt 6% + Aln 1%
37		16SA-7	Trondhjemite	3232.7	26º05'22.57''	30º50'47.35''	Pl 45% + Qz 22% + Kf 20% + Bt 13%
38		16SA-8-1	Trondhjemite	3232	26º05'25.40''	30º50'43.23''	Pl 45% + Qz 25% + Kf 18% + Bt 12%
39		16SA-8-2	Trondhjemite	3229.5	26º05'25.40''	30º50'43.23''	Pl 50% + Qz 25% + Bt 20% + Kf 5%
40		16SA-9-1	Trondhjemite	3217.8	26º04'52.30''	30º51'48.79''	Pl 55% + Qz 20% + Kf 14% + Bt 11%
41		16SA-9-2	Trondhjemite	3230	26º04'52.30''	30º51'48.79''	Pl 35% + Qz 40% + Bt 20% + Kf 5%
42		16SA-9-3	Tonalite	3232	26º04'52.30''	30º51'48.79''	Pl 40 % + 40% Qz + Bt 11% + Kf 8% + Sph 1%
43		16SA-6-2	Tonalite	3232	26º04'51.49''	30º51'14.78''	Pl 55% + Qz 32% + Hb 15% + Bt 8%
44		16SA-28-1	Trondhjemite	3250	26º05'10.96''	30º23'41.01''	Pl 50% + Qz 30% + Bt 17% + Kf 3%

45	Rooihoogte Pass	16SA-28-2	Trondhjemite	3245	26º05'10.96''	30º23'41.01''	Pl 45% + Qz 40% + Bt 13% + Kf 2%
46		16SA-29	Trondhjemite	3244.3	26º05'22.56''	30º24'00.14''	Pl 50% + Qz 30% + Bt 15% + minor Sph, Aln, Ep and Ap
47		16SA-30	Trondhjemite	3245	26º03'25.44''	30º23'29.89''	Pl 62% + Qz 25% + Bt 8% + Kf 5%
48		16SA-31-1	Trondhjemite	3242.9	26º00'38.24''	30º27'02.72''	Pl 50% + Qz 25% + Bt 15% + Kf 10%
49		16SA-31-2	Trondhjemite	3248.3	26º00'38.24''	30º27'02.72''	Pl 55% + Qz 25% + Bt 12% + Kf 8%
50	Weergevonden	16SA-36-1	Trondhjemite	3464.1	26º05'24.22''	30º43'59.99''	Pl 50% + Qz 35% + Bt 10% + Kf 5%
51		16SA-36-2	Tonalite	3232.2	26º05'24.22''	30º43'59.99''	Pl 35% + Qz 30% + Hb 20% + Bt 15%

Notes: Abbreviation: Pl - plagioclase; Qz - quartz; Hb - hornblende; Bt - biotite; Kf - K-feldspar; Aln - allanite; Sph - sphene; Ep - epidote; Ap - apatite. Sometimes we collected few samples that may have different ages in one location, and in this regard, only one representative sample is shown in Fig. 1A. Detailed ages can be found in the concordia diagrams and Table S3.

Supplementary Table S2 Major (wt%) and trace element (ppm) analyses of the BGGT TTGs in South Africa

No.	1	2	3	4	5	6	7	8	9	10
Sample	14SA-18-1	16SA-20-2	17SA-12	17SA-13-1	17SA-14-1	17SA-14-2	16SA-11	16SA-33-1	16SA-33-2	16SA-34
Rock type	Trond.	Tonalite	Trond.	Trond.	Trond.	Trond.	Trond.	Trond.	Trond.	Trond.
Pluton/Intrusion	Badplaas						Batavia			
Age (Ma)	3266	3251	*3235*	*3260*	3234	3233	3459	3253	*3250*	3238
$\delta^{18}O$ (‰)	5.64	5.85	5.12	-	5.68	5.50	5.76	5.39	-	5.88
2SD Error (‰)	0.51	0.1	0.24	-	0.12	0.15	0.21	0.41	-	0.27
SiO_2	70.85	65.85	70.37	71.94	69.48	68.98	71.52	72.29	73.79	70.19
TiO_2	0.09	0.33	0.37	0.21	0.23	0.26	0.15	0.14	0.19	0.17
Al_2O_3	18.32	18.27	15.60	15.66	16.94	17.19	15.64	15.37	14.99	16.29
Fe_2O_3	0.01	2.22	2.87	1.38	2.39	2.17	0.60	1.04	1.10	1.38
MnO	0.00	0.04	0.02	0.02	0.03	0.04	0.01	0.02	0.02	0.04
MgO	0.17	1.51	0.98	0.57	0.87	0.63	0.73	0.35	0.61	0.90
CaO	4.01	6.87	3.05	1.56	3.51	3.60	1.49	1.97	1.86	2.02
Na_2O	6.05	4.34	4.61	5.79	4.88	5.20	5.80	5.45	5.09	6.11
K_2O	0.35	0.27	1.21	1.32	1.02	0.89	2.28	1.79	1.78	1.83
P_2O_5	0.03	0.14	0.10	0.07	0.09	0.10	0.04	0.05	0.05	0.06
LOI	0.40	0.40	0.43	1.07	0.81	0.88	0.80	0.60	0.20	0.58
TOTAL	100.27	100.24	99.62	99.58	100.25	99.93	99.07	99.08	99.67	99.59
Li	5.72	0.41	1.87	14.2	2.49	1.39	22.5	30.1	34.6	21.2
Be	0.96	0.78	0.83	0.71	0.85	0.76	1.85	1.18	1.24	1.28
Sc	6.27	4.06	2.46	2.00	1.62	2.24	1.64	1.79	1.84	2.04
V	6.02	49.1	36.6	12.6	17.1	13.9	9.81	11.6	15.4	22.9
Cr	4.32	14.9	10.9	4.64	4.24	2.01	15.1	8.61	11.1	8.26
Co	1.43	9.06	7.43	3.01	5.13	2.69	2.67	1.97	2.88	2.55
Ni	3.62	7.97	6.34	2.91	2.14	0.51	6.77	2.09	3.43	2.03
Cu	3.43	39.9	2.68	6.00	6.23	1.24	1.57	0.72	3.43	3.91
Zn	29.3	29.3	64.7	24.2	42.3	34.9	27.9	27.8	39.7	39.2
Ga	14.8	13.7	14.6	15.8	12.8	13.8	16.4	15.2	14.3	16.3
As	0.70	12.9	1.44	2.45	1.87	2.58	11.9	11.2	11.5	11.3
Rb	5.11	1.33	43.2	30.3	48.9	34.1	42.6	43.3	46.5	54.6
Sr	416	496	469	429	529	602	582	358	323	327
Y	1.74	7.42	4.54	2.63	2.97	5.20	3.45	3.28	3.53	4.81
Zr	48.0	198	147	123	119	125	95.8	79.0	89.4	83.8
Nb	0.50	1.59	3.70	1.31	2.30	2.16	2.91	3.29	4.33	2.40
Cs	0.75	0.72	1.95	0.78	7.32	2.63	0.77	1.01	1.29	1.11
Ba	67.2	27.8	205	194	127	107	512	184	175	285
La	11.6	20.9	13.9	17.0	10.5	9.82	15.9	5.70	9.50	14.8
Ce	21.8	46.1	27.9	29.9	20.7	21.4	25.5	15.5	21.5	24.8
Pr	2.55	4.97	3.35	3.31	2.16	2.33	2.90	1.45	2.05	2.94
Nd	9.32	18.6	12.4	11.3	7.83	8.58	10.3	5.36	7.40	10.9
Sm	1.65	3.01	2.22	1.66	1.31	1.58	1.66	1.07	1.43	1.88
Eu	0.47	0.86	0.68	0.56	0.47	0.54	0.50	0.42	0.43	0.57
Gd	1.08	2.12	1.72	1.01	1.03	1.30	1.13	0.83	1.09	1.45
Tb	0.14	0.28	0.21	0.11	0.13	0.17	0.14	0.12	0.15	0.20
Dy	0.50	1.34	0.98	0.47	0.61	0.92	0.61	0.59	0.71	0.88
Ho	0.095	0.25	0.16	0.08	0.10	0.18	0.10	0.10	0.12	0.15
Er	0.16	0.71	0.36	0.23	0.24	0.50	0.28	0.28	0.31	0.37
Tm	0.02	0.11	0.04	0.03	0.03	0.08	0.04	0.04	0.04	0.05
Yb	0.12	0.66	0.24	0.23	0.21	0.51	0.24	0.24	0.26	0.27
Lu	0.02	0.10	0.04	0.03	0.03	0.08	0.04	0.04	0.04	0.04
Hf	1.23	4.11	3.36	2.73	2.84	2.96	2.68	2.08	2.46	2.18
Ta	0.04	0.14	0.24	0.07	0.09	0.12	0.26	0.29	0.28	0.18
Pb	4.04	3.15	3.54	2.64	4.61	3.76	6.60	4.71	4.29	3.98
Th	2.62	3.53	1.84	2.01	1.71	1.32	2.54	1.01	1.54	2.32
U	0.30	0.54	0.21	0.48	0.25	0.31	0.80	0.19	0.25	0.41
Sr/Y	239	66.9	103	163	178	116	169	109	91.5	68.0
La/Yb	96.7	31.7	57.8	73.8	50.1	19.3	66.6	24.1	36.8	54.6

Note: 1) The age results in italic font are just given by the age of adjacent samples;

2) -, not detected;

3) Trond., Trondhjemite.

11	12	13	14	15	16	17	18	19	20	21
16SA-3-4	16SA-17	17SA-7	17SA-8	14SA-23	14SA-25	16SA-1	14SA-16	14SA-20-1	14SA-20-2	14SA-21
Trond.	Trond.	Trond.	Trond.	Tonalite	Tonalite	Granite	Trond.	Trond.	Trond.	Trond.
Eerstehoek	Honingklip			Kaap Valley		Nederland	Nelshoogte	Stolzburg		
3428	*3220*	*3220*	3216	3222	3226	3106	3232	3452	3215	3454
5.21	-	-	6.96	6.14	6.21	6.69	5.92	5.49	5.95	6.02
0.16	-	-	0.51	0.10	0.13	0.24	0.09	0.13	0.25	0.15
72.24	69.56	72.45	73.18	61.04	66.01	68.46	71.07	70.82	67.16	67.67
0.28	0.34	0.25	0.29	0.52	0.40	0.51	0.32	0.22	0.38	0.30
15.37	16.46	15.15	14.62	16.49	15.81	15.05	16.25	16.98	16.70	16.23
1.73	1.81	1.65	1.86	4.97	3.80	2.97	2.02	1.15	2.65	2.27
0.03	0.02	0.03	0.03	0.07	0.04	0.05	0.02	0.03	0.04	0.03
0.98	0.79	0.40	0.48	2.78	1.58	0.91	0.92	0.50	0.79	1.26
3.02	2.68	1.91	2.05	4.24	2.43	1.49	3.00	2.15	2.64	2.83
4.71	5.53	4.89	4.78	4.94	5.24	4.70	5.07	6.21	6.10	5.77
1.26	1.74	2.59	2.04	1.35	1.03	4.65	1.41	1.81	1.38	1.57
0.08	0.10	0.07	0.08	0.22	0.14	0.28	0.09	0.04	0.16	0.08
0.80	0.98	0.72	0.68	3.01	2.75	0.80	0.39	0.40	1.99	2.72
100.51	100.01	100.12	100.08	99.62	99.23	99.86	100.57	100.30	99.99	100.72
147	18.8	49.4	44.6	15.3	6.27	14.3	32.4	39.7	51.3	33.4
1.81	1.97	2.20	1.59	1.44	0.9	6.57	0.79	2.38	2.62	1.41
1.90	1.89	2.37	2.08	15.2	11.4	5.45	6.62	8.39	7.43	8.24
21.7	30.2	10.2	14.0	92.8	71.9	40.6	25.2	10.3	23.6	26.0
27.6	10.0	4.26	3.54	71.1	49.9	14.3	13.5	10.3	7.13	25.2
5.05	4.45	2.91	3.50	16.7	12.2	5.48	5.49	3.01	4.86	7.83
14.6	3.02	1.78	1.41	41.5	29.5	5.16	11.9	8.35	4.51	33.5
2.19	2.27	2.95	3.96	25.5	5.94	14.7	9.02	1.85	10.4	7.44
43.6	58.5	56.5	59.7	94.3	111	64.0	60.9	63.7	88.1	74.1
13.4	16.6	20.2	18.6	19.5	18.1	24.8	17.9	21.7	19.1	20.1
11.4	12.3	1.83	1.87	1.41	1.68	15.6	0.81	1.28	0.71	0.77
52.9	39.2	80.4	55.7	39.5	29.6	165	24.8	46.8	44.6	32.3
280	626	488	499	533	610	884	454	457	615	516
8.41	4.71	6.40	6.67	12.1	7.44	29.6	3.18	4.17	5.45	5.59
135	139	111	126	130	97.2	319	123	99.7	161	130
7.79	3.90	5.63	5.88	5.19	3.09	19.7	2.25	4.77	9.24	4.30
5.69	2.05	5.59	2.96	0.78	0.59	3.14	1.54	2.70	4.18	1.73
169	369	534	437	326	239	1111	167	215	482	324
13.5	20.2	17.7	18.4	21.8	11.3	91.3	10.6	10.0	25.5	8.61
26.8	39.6	33.2	35.7	46.4	24.5	185	19.5	18.2	48.7	16.0
2.93	4.46	4.11	4.33	5.52	2.89	23.8	2.12	2.07	6.05	1.93
11.3	16.8	14.2	15.3	21.6	10.9	89.4	7.31	7.65	23.0	7.71
2.27	2.83	2.70	2.99	4.04	2.21	13.4	1.11	1.34	3.76	1.64
0.80	0.82	0.73	0.79	1.22	0.81	3.07	0.48	0.38	0.91	0.59
2.08	1.89	2.01	2.23	3.75	2.04	9.84	0.85	1.18	2.45	1.31
0.30	0.23	0.25	0.29	0.47	0.26	1.30	0.11	0.14	0.31	0.19
1.61	0.94	1.17	1.28	2.42	1.37	5.68	0.55	0.67	1.22	1.02
0.29	0.16	0.19	0.21	0.49	0.29	0.96	0.12	0.15	0.26	0.23
0.74	0.44	0.48	0.51	1.27	0.68	2.54	0.30	0.37	0.51	0.53
0.10	0.06	0.07	0.07	0.17	0.10	0.35	0.04	0.05	0.06	0.06
0.62	0.34	0.41	0.41	0.92	0.69	2.10	0.28	0.34	0.37	0.39
0.09	0.06	0.06	0.06	0.14	0.10	0.31	0.05	0.04	0.05	0.06
3.42	3.55	2.78	3.08	3.03	2.67	7.66	3.01	2.82	3.70	3.19
0.65	0.23	0.40	0.38	0.30	0.42	1.52	0.16	0.50	0.62	0.36
6.82	11.3	15.5	11.8	6.34	6.42	28.5	4.42	11.8	11.1	8.39
2.80	3.46	2.99	2.86	3.81	2.19	17.8	1.19	3.29	5.27	2.44
0.47	1.27	1.62	1.10	0.88	0.48	4.29	0.29	1.04	1.51	0.77
33.3	133	76.2	74.7	44.0	82.0	29.9	143	110	113	92.3
21.8	59.5	42.6	44.8	23.7	16.4	43.5	37.9	29.4	68.9	22.1

22	23	24	25	26	27	28	29	30	31	32
14SA-21-3	16SA-12	16SA-18-2	16SA-18-3	14SA-5-1	14SA-11	14SA-22	16SA-10-1	17SA-9-1	17SA-10-1	16SA-4-1
Trond.	Trond.	Trond.	Trond.	Trond.	Trond.	Trond.	Trond.	Tonalite	Tonalite	Trond.
Stolzburg				Theespruit						Uitgevonden
3447	*3450*	3206	3423	3452	3448	3453	3460	*3450*	3453	*3232*
5.72	-	5.95	5.07	5.26	5.75	5.86	5.96	-	5.72	-
0.2	-	0.25	0.2	0.53	0.11	0.25	0.12	-	0.14	-
72.37	69.87	68.81	72.03	71.94	71.44	68.76	71.32	72.10	71.07	70.33
0.19	0.30	0.36	0.11	0.27	0.30	0.29	0.22	0.29	0.30	0.28
14.37	16.58	16.78	15.21	15.61	15.17	15.90	15.55	14.37	14.86	15.51
1.40	1.90	2.16	0.76	1.57	1.91	2.19	1.41	2.35	2.39	1.83
0.02	0.03	0.04	0.01	0.03	0.03	0.04	0.03	0.04	0.04	0.03
0.95	1.01	0.79	0.30	1.03	1.27	1.12	1.10	1.09	1.25	1.20
2.46	3.41	2.79	1.57	2.36	2.66	2.52	2.45	2.42	2.68	2.67
5.43	5.37	5.65	5.53	5.42	5.23	5.32	5.42	4.64	4.99	5.25
0.33	0.80	1.66	2.66	1.65	1.66	1.79	1.74	1.49	1.50	1.42
0.05	0.08	0.14	0.04	0.06	0.07	0.07	0.06	0.07	0.08	0.07
1.67	0.60	0.78	0.98	0.19	0.58	1.20	0.98	0.75	0.64	0.97
99.24	99.94	99.97	99.20	100.13	100.33	99.18	100.26	99.61	99.79	99.55
21.3	5.57	41.6	27.1	28.0	24.6	56.6	16.6	24.3	11.5	5.60
1.03	0.86	2.06	1.89	1.57	1.53	1.85	1.71	1.61	1.39	1.66
6.84	1.84	3.24	1.17	9.4	9.01	9.01	2.19	2.21	2.64	2.92
21.7	32.9	24.6	7.64	18.4	24.4	25.1	40.6	20.1	20.8	25.5
28.8	21.8	11.5	10.6	19.4	31.8	18.4	22.8	17.1	21.4	30.6
6.54	5.29	4.48	1.37	5.73	7.6	6.41	5.05	6.43	7.36	6.17
23.7	7.63	3.10	2.86	25.1	39.2	22.8	19.9	23.0	30.3	22.9
44.1	4.84	3.17	1.24	3.91	4.44	2.17	0.22	0.08	1.21	0.40
74.8	46.6	81.8	26.2	105	86.2	68.9	44.6	49.2	51.0	43.4
13.8	12.5	19.1	15.0	18.9	19.2	18.2	14.8	23.6	15.1	14.8
0.66	11.4	12.1	11.4	2.18	1.12	1.37	11.5	4.0	2.6	11.8
13.0	23.0	55.3	45.1	33.9	58.8	35.5	54.1	74.2	47.7	40.0
239	559	704	468	424	537	370	520	401	534	476
4.46	3.82	8.27	1.88	7.03	6.60	6.92	4.81	7.71	5.87	6.64
121	126	168	74.9	135	129	139	117	136	136	128
4.63	2.76	11.2	2.58	5.82	4.91	6.22	4.34	7.15	5.26	5.49
0.58	1.49	2.98	1.33	0.87	1.85	2.72	2.02	4.72	2.04	0.69
60.3	101	827	1137	385	345	273	326	251	291	275
7.41	5.24	27.5	8.19	16.5	43.3	10.9	10.4	106	10.3	13.4
13.4	12.3	54.2	14.7	29.7	71.9	18.0	18.2	180	21.4	23.7
1.38	1.38	6.34	1.47	3.10	7.14	2.05	1.94	16.2	2.28	2.52
5.12	5.66	24.6	4.82	10.1	23.7	7.01	7.21	48.9	8.62	9.40
0.94	1.21	4.44	0.72	1.82	3.05	1.45	1.35	5.34	1.83	1.77
0.37	0.53	1.23	0.34	0.55	0.70	0.52	0.49	0.77	0.58	0.59
0.75	1.07	3.14	0.49	1.43	2.13	1.49	1.10	2.99	1.47	1.51
0.12	0.14	0.39	0.06	0.20	0.27	0.21	0.15	0.36	0.21	0.22
0.62	0.74	1.61	0.28	1.03	1.23	1.06	0.81	1.49	1.10	1.12
0.14	0.13	0.25	0.05	0.23	0.25	0.22	0.15	0.26	0.20	0.21
0.40	0.35	0.64	0.15	0.63	0.68	0.63	0.42	0.76	0.55	0.58
0.06	0.05	0.09	0.02	0.09	0.08	0.09	0.07	0.10	0.08	0.09
0.37	0.31	0.48	0.15	0.59	0.53	0.63	0.40	0.68	0.49	0.52
0.05	0.05	0.06	0.02	0.08	0.08	0.09	0.06	0.10	0.06	0.08
3.00	2.97	4.00	2.03	3.71	3.40	3.21	2.97	3.60	3.30	3.17
0.62	0.17	0.89	0.16	0.53	0.48	0.69	0.40	0.88	0.47	0.44
4.15	2.42	11.2	9.90	13.6	9.62	12.6	9.43	12.3	7.61	6.79
2.05	0.63	5.24	1.78	5.00	8.39	3.68	2.76	17.0	2.75	3.24
0.51	0.19	1.53	0.50	1.20	0.98	1.06	0.79	1.47	1.02	0.80
53.6	146	85.1	249	60.3	81.4	53.5	108	52.0	91.1	71.6
20.0	16.9	57.2	56.1	28.0	81.7	17.3	25.9	154	21.0	25.6

33	34	35	36	37	38	39	40	41	42	43
16SA-4-2	16SA-4-3	16SA-5-1	16SA-6-1	16SA-7	16SA-8-1	16SA-8-2	16SA-9-1	16SA-9-3	16SA-9-2	16SA-6-2
Trond.	Trond.	Trond.	Trond.	Trond.	Trond.	Trond.	Trond.	Trond.	Tonalite	Tonalite
					Uitgevonden					
3232	*3232*	*3232*	*3232*	3233	*3232*	3230	3218	*3232*	3230	*3232*
-	-	-	-	6.58	-	6.66	6.34	-	-	-
-	-	-	-	0.21	-	0.14	0.31	-	-	-
70.30	70.63	71.42	72.30	70.09	70.33	69.46	68.45	71.10	64.42	60.61
0.26	0.26	0.28	0.24	0.30	0.31	0.38	0.38	0.31	0.63	0.65
15.93	15.58	15.51	15.54	16.32	16.12	16.97	16.77	15.85	16.88	16.07
1.72	1.73	1.37	1.16	1.71	1.90	1.91	2.18	1.68	3.64	6.97
0.03	0.03	0.03	0.03	0.02	0.02	0.02	0.03	0.02	0.05	0.14
1.12	1.22	0.85	0.66	0.80	0.76	0.88	0.90	0.73	1.34	2.19
2.78	2.75	0.98	1.96	2.41	2.42	2.82	2.36	2.41	3.42	4.44
5.46	5.37	6.14	5.09	5.50	5.39	5.87	5.39	5.10	5.39	4.69
1.44	1.57	1.89	2.50	1.71	2.02	1.23	2.35	2.32	1.87	2.49
0.07	0.07	0.07	0.07	0.08	0.09	0.09	0.12	0.10	0.24	0.38
0.00	0.79	0.39	0.79	0.59	0.39	0.59	0.96	0.40	1.09	0.79
99.11	100.02	98.92	100.34	99.53	99.77	100.22	99.88	100.02	98.97	99.41
2.15	5.18	29.0	17.7	31.2	33.1	28.4	27.7	25.1	40.7	63.6
1.90	1.59	2.56	1.97	1.72	1.73	1.54	2.36	2.05	1.42	1.99
2.37	2.58	2.64	1.82	2.58	2.52	2.36	3.20	1.92	5.11	9.43
26.2	24.6	18.7	19.8	21.7	25.7	25.0	32.8	20.8	52.4	69.7
20.2	24.0	16.6	20.6	19.3	59.4	12.5	14.3	17.2	17.6	30.9
5.52	6.00	3.96	3.27	4.44	4.72	4.95	5.25	3.91	8.20	12.7
17.8	21.7	5.36	4.74	6.61	22.5	4.69	3.92	5.16	6.07	8.22
1.62	0.25	1.04	13.1	0.67	17.9	4.42	8.85	5.08	13.9	2.13
35.4	40.4	21.5	46.6	56.8	56.9	59.8	62.9	54.4	93.2	119
14.2	15.0	15.0	16.1	16.9	17.1	17.0	18.8	15.1	21.2	22.7
11.4	11.9	11.7	11.9	11.8	11.9	12.2	12.3	11.7	13.4	13.6
39.8	43.6	53.7	58.8	48.3	51.2	42.8	63.5	66.3	54.3	95.1
482	484	335	531	617	592	699	592	503	1089	946
6.05	7.82	5.98	5.21	4.59	6.50	5.98	7.00	5.72	11.8	21.5
117	131	129	105	135	125	137	146	119	253	198
5.47	5.58	3.89	4.22	4.24	4.72	3.93	4.88	4.36	7.15	12.7
0.68	0.81	0.95	1.12	4.09	2.84	3.59	3.71	24.0	3.76	6.28
261	310	529	472	462	597	267	545	658	901	923
13.5	19.3	15.7	16.7	16.1	20.6	24.1	23.1	18.1	59.2	55.8
24.7	29.4	36.6	32.2	33.7	40.4	44.8	46.1	34.3	115	113
2.56	3.46	3.61	3.52	3.58	4.49	5.25	5.24	3.98	13.7	13.6
9.51	12.6	13.9	13.3	13.5	16.7	19.6	20.1	15.2	51.8	53.5
1.76	2.24	2.64	2.30	2.35	2.87	3.30	3.57	2.75	7.83	8.54
0.58	0.69	0.72	0.64	0.66	0.86	0.97	0.97	0.78	2.18	2.35
1.44	1.86	1.92	1.66	1.63	2.09	2.35	2.55	2.05	5.39	6.48
0.21	0.26	0.25	0.21	0.21	0.27	0.30	0.33	0.26	0.65	0.88
1.08	1.34	1.17	0.97	0.99	1.18	1.26	1.45	1.15	2.64	4.15
0.20	0.25	0.20	0.17	0.17	0.20	0.20	0.23	0.19	0.41	0.73
0.55	0.67	0.53	0.44	0.45	0.53	0.54	0.59	0.48	1.07	1.96
0.08	0.10	0.07	0.06	0.06	0.07	0.07	0.08	0.06	0.13	0.27
0.50	0.56	0.42	0.36	0.38	0.40	0.38	0.45	0.35	0.72	1.56
0.07	0.08	0.06	0.05	0.05	0.06	0.05	0.06	0.05	0.10	0.22
2.91	3.31	3.05	2.67	3.21	3.03	3.15	3.45	2.98	5.34	4.69
0.40	0.40	0.27	0.27	0.26	0.33	0.23	0.34	0.36	0.45	0.78
6.72	7.09	3.62	13.7	10.5	10.7	7.83	10.2	10.2	10.1	13.8
3.29	4.36	2.83	2.76	3.02	4.27	3.59	3.44	2.78	10.7	7.86
0.76	0.85	0.68	0.89	0.60	0.80	0.46	1.04	0.57	1.07	2.28
79.7	61.9	56.0	102	134	91.1	117	84.6	88.1	91.9	44.0
26.9	34.3	37.6	46.7	42.5	51.3	63.0	51.9	51.9	82.1	35.7

44	45	46	47	48	49	50	51
16SA-28-1	16SA-28-2	16SA-29	16SA-30	16SA-31-1	16SA-31-2	16SA-36-1	16SA-36-2
Trond.	Trond.	Trond.	Trond.	Trond.	Trond.	Trond.	Tonalite
Rooihoogte Pass						Weergevonden	
3250	*3245*	3244	*3245*	3243	3248	3464	3232
5.73	-	5.70	-	5.74	5.87	5.60	7.08
0.23	-	0.12	-	0.19	0.13	0.18	0.13
71.19	71.76	68.40	68.14	71.50	69.09	72.17	57.90
0.32	0.30	0.41	0.41	0.21	0.31	0.18	1.14
15.97	15.85	16.98	17.32	15.93	16.01	16.10	15.11
2.04	1.92	2.35	2.33	1.31	2.29	0.81	7.41
0.03	0.03	0.04	0.03	0.04	0.05	0.01	0.07
0.81	0.74	1.07	1.35	0.99	1.49	0.71	5.39
2.96	2.98	3.61	3.24	2.18	3.25	2.26	6.67
5.03	4.98	5.11	5.05	5.33	5.00	5.82	4.09
1.09	1.04	1.13	1.12	1.59	0.83	1.54	1.16
0.10	0.11	0.13	0.12	0.07	0.10	0.05	0.34
0.40	0.40	0.39	1.00	1.18	1.18	0.40	1.00
99.94	100.11	99.62	100.10	100.33	99.59	100.06	100.29
22.4	24.7	67.2	9.70	23.5	14.9	-	79.6
0.72	0.73	3.16	0.91	1.43	1.16	-	1.50
2.00	2.20	3.43	2.06	2.23	2.37	-	17.5
17.6	16.9	39.9	46.9	23.2	42.1	-	158
10.7	9.88	12.8	10.9	17.9	27.8	-	138
3.95	3.70	5.96	5.99	3.73	7.23	-	35.5
2.23	2.12	5.88	5.57	6.71	16.7	-	71.7
4.42	6.95	14.9	23.0	11.2	5.79	-	68.9
41.2	40.2	44.1	37.2	33.4	44.1	-	108
12.5	12.9	15.1	13.4	14.4	13.3	-	20.0
11.3	11.8	11.3	11.6	10.8	11.6	-	13.3
30.5	30.5	44.5	28.5	37.7	27.3	-	32.4
498	496	533	489	428	442	-	759
3.91	4.43	5.67	4.08	5.70	6.40	-	14.2
168	178	158	123	94.2	131	-	97.3
2.37	2.80	5.21	2.95	3.16	4.09	-	3.73
0.93	1.01	4.69	2.30	1.68	1.10	-	2.27
105	117	116	101	186	90.4	-	265
14.9	19.6	18.1	13.0	11.8	11.7	-	21.0
30.2	37.5	37.8	26.2	23.3	26.3	-	50.1
2.92	3.70	3.85	2.64	2.49	2.57	-	6.75
10.2	12.8	14.0	9.45	9.22	9.77	-	30.6
1.45	1.87	2.22	1.52	1.62	1.86	-	6.62
0.56	0.66	0.69	0.56	0.51	0.62	-	1.77
1.04	1.36	1.69	1.16	1.25	1.57	-	5.23
0.14	0.18	0.23	0.16	0.18	0.23	-	0.67
0.68	0.81	1.12	0.80	0.94	1.20	-	3.19
0.13	0.15	0.20	0.14	0.17	0.22	-	0.54
0.40	0.43	0.54	0.38	0.50	0.60	-	1.32
0.06	0.06	0.07	0.05	0.08	0.09	-	0.17
0.40	0.40	0.42	0.28	0.49	0.54	-	0.92
0.06	0.07	0.06	0.04	0.07	0.08	-	0.12
3.56	3.80	3.60	2.89	2.46	3.13	-	2.41
0.11	0.11	0.69	0.15	0.32	0.33	-	0.19
2.78	3.15	3.72	2.70	4.91	4.00	-	5.80
2.03	2.86	2.19	1.62	1.69	1.61	-	2.23
0.33	0.38	0.61	0.20	0.83	0.60	-	0.89
127	112	93.9	120	75.1	69.0	-	53.5
37.4	48.6	43.5	46.8	24.1	21.6	-	22.8

Supplementary Table S3 SIMS zircon U-Pb isotope results for the BGGT TTGs in South Africa (Part I-by CAMECA)

Sample/ spot #	(^{238}U/ ^{206}Pb)$_{uc}$	±σ %	(^{207}Pb/ ^{206}Pb)$_{uc}$	±σ %	(^{207}Pb/ ^{235}U)$_c$	±σ %	(^{206}Pb/ ^{238}U)$_c$	±σ %	ρ	Disc. %	^{207}Pb/ ^{206}Pb	±σ	^{207}Pb/ ^{235}U	±σ	^{206}Pb/ ^{238}U	±σ	[U] ppm	[Th] ppm	[Pb] ppm	Th/U	f_{206}%
**Mount T289**																					
14SA-11@01	1.4414	1.61	0.2956	0.35	28.27	1.65	0.6938	1.61	0.98	-1.9	3447.1	5.4	3428.6	16.3	3397.0	42.6	153	51	147	0.33	{0.01}
14SA-11@02	1.4508	1.62	0.2972	0.15	28.24	1.62	0.6893	1.62	1.00	-2.8	3455.6	2.3	3427.6	16.0	3379.8	42.6	175	66	168	0.38	{0.00}
14SA-11@03	1.4465	2.03	0.2961	0.61	28.21	2.12	0.6912	2.03	0.96	-2.3	3449.3	9.4	3426.4	21.0	3387.2	53.7	125	43	120	0.34	0.02
14SA-11@04	1.4229	1.61	0.2964	0.15	28.70	1.62	0.7026	1.61	1.00	-0.8	3450.6	2.4	3443.2	16.0	3430.4	42.9	165	64	162	0.39	0.03
14SA-11@05	1.4749	1.61	0.2956	0.13	27.63	1.61	0.6779	1.61	1.00	-4.1	3447.2	2.0	3406.1	15.9	3336.5	42.0	241	103	230	0.43	0.01
14SA-11@06	1.5396	1.61	0.2894	0.26	25.89	1.63	0.6493	1.61	0.99	-7.0	3413.3	4.0	3342.4	16.1	3225.4	41.1	200	79	181	0.39	0.03
14SA-11@07	1.4578	1.61	0.2928	0.16	27.68	1.61	0.6859	1.61	0.99	-2.4	3431.8	2.5	3407.7	15.9	3366.8	42.3	250	99	239	0.40	0.02
14SA-11@08	1.4297	1.61	0.2962	0.21	28.56	1.63	0.6994	1.61	0.99	-1.2	3450.1	3.2	3438.3	16.1	3418.2	42.9	141	48	137	0.34	0.01
14SA-11@09	4.3484	1.61	0.2166	0.21	6.60	1.63	0.2276	1.61	0.99	-60.1	2907.4	4.0	2059.1	14.5	1322.0	19.3	1136	674	350	0.59	1.02
14SA-11@10	1.5063	1.61	0.2975	0.17	27.07	1.62	0.6624	1.61	0.99	-6.4	3451.2	2.7	3385.9	16.0	3276.6	41.5	167	77	156	0.46	0.22
14SA-11@11	1.4618	1.83	0.2968	0.45	27.98	1.89	0.6839	1.83	0.97	-3.5	3453.0	7.0	3418.3	18.7	3359.4	48.2	197	90	191	0.46	0.02
14SA-11@12	1.4749	1.61	0.2964	0.17	27.68	1.62	0.6778	1.61	0.99	-4.2	3450.5	2.6	3407.9	16.0	3336.0	42.0	136	41	127	0.30	0.03
14SA-21@01	1.4153	1.61	0.2964	0.33	28.86	1.64	0.7065	1.61	0.98	-0.2	3451.0	5.0	3448.9	16.2	3445.3	43.1	176	65	173	0.37	0.01
14SA-21@2	1.4118	1.61	0.2968	0.18	28.98	1.62	0.7083	1.61	0.99	-0.1	3453.3	2.8	3452.8	16.0	3451.9	43.1	159	45	155	0.28	0.01
14SA-21@3	1.5030	1.75	0.2979	0.24	27.20	1.77	0.6642	1.75	0.99	-6.3	3454.6	3.8	3390.7	17.5	3283.5	45.2	163	59	152	0.36	0.17
14SA-21@4	1.3819	1.71	0.2945	0.37	29.38	1.75	0.7236	1.71	0.98	2.6	3441.5	5.8	3466.4	17.4	3509.6	46.5	114	44	115	0.38	{0.01}
14SA-21@5	1.4078	1.62	0.2949	0.26	28.88	1.64	0.7103	1.62	0.99	0.6	3443.7	4.0	3449.6	16.2	3459.8	43.5	138	50	136	0.36	{0.00}
14SA-21@6	1.4571	1.61	0.2975	0.12	28.15	1.61	0.6863	1.61	1.00	-3.3	3457.4	1.9	3424.4	15.9	3368.4	42.3	258	108	249	0.42	{0.01}
14SA-21@7	1.4303	1.61	0.2964	0.28	28.53	1.63	0.6988	1.61	0.99	-1.3	3450.1	4.3	3437.6	16.1	3416.1	42.8	175	62	170	0.35	0.05
14SA-21@8	1.4038	1.61	0.2972	0.16	29.19	1.62	0.7124	1.61	0.99	0.4	3455.8	2.5	3460.1	16.0	3467.4	43.2	277	120	279	0.43	{0.00}
14SA-22@01	1.4011	1.68	0.2953	0.30	29.04	1.71	0.7135	1.68	0.98	1.0	3444.9	4.7	3454.8	16.9	3471.8	45.2	180	64	179	0.35	0.03
14SA-22@2	1.4436	1.64	0.2976	0.54	28.41	1.72	0.6926	1.64	0.95	-2.4	3457.2	8.4	3433.4	17.0	3392.6	43.3	158	54	152	0.34	0.02
14SA-22@3	1.3696	1.62	0.2960	0.55	29.77	1.71	0.7298	1.62	0.95	3.2	3448.4	8.5	3479.1	16.9	3532.7	44.1	161	54	162	0.34	0.04
14SA-22@4	1.4398	1.61	0.2974	0.13	28.38	1.61	0.6936	1.61	1.00	-2.1	3453.3	2.0	3432.3	15.9	3396.5	42.6	228	93	222	0.41	0.13
14SA-22@5	1.4118	1.83	0.2968	0.51	28.98	1.90	0.7083	1.83	0.96	0.0	3453.4	7.9	3452.9	18.8	3452.1	49.0	205	65	200	0.31	{0.01}
14SA-22@6	1.6303	1.61	0.2988	0.17	25.08	1.62	0.6118	1.61	0.99	-13.8	3456.6	2.8	3311.4	15.9	3077.1	39.5	251	105	217	0.42	0.26
14SA-22@7	1.4595	1.63	0.2971	0.20	27.93	1.64	0.6840	1.63	0.99	-3.4	3450.3	3.2	3416.7	16.2	3359.6	42.7	107	43	100	0.40	0.17
14SA-22@8	1.5357	1.61	0.2951	0.15	26.14	1.62	0.6481	1.61	0.99	-7.8	3431.3	2.7	3351.9	15.9	3220.8	40.9	210	98	193	0.47	0.47
14SA-22@9	1.4508	1.61	0.2969	0.33	28.18	1.65	0.6889	1.61	0.98	-2.8	3452.6	5.2	3425.2	16.3	3378.6	42.6	209	77	201	0.37	0.05
14SA-23@01	1.5505	1.61	0.2561	0.42	22.78	1.66	0.6450	1.61	0.97	-0.6	3223.1	6.6	3217.5	16.3	3208.4	40.9	67	35	60	0.53	{0.00}
14SA-23@2	1.5515	1.61	0.2551	0.31	22.64	1.64	0.6443	1.61	0.98	-0.4	3215.3	4.8	3211.8	16.1	3206.0	40.8	72	40	65	0.55	0.03
14SA-23@3	1.5543	1.62	0.2555	0.37	22.67	1.66	0.6434	1.62	0.97	-0.7	3219.4	5.9	3212.8	16.3	3202.2	41.0	37	18	33	0.48	{0.00}
14SA-23@4	1.5908	1.61	0.2571	0.27	22.21	1.64	0.6280	1.61	0.99	-3.3	3225.5	4.4	3193.1	16.0	3141.7	40.3	58	30	51	0.51	0.10
14SA-23@5	1.5496	1.61	0.2559	0.20	22.77	1.62	0.6453	1.61	0.99	-0.5	3221.7	3.2	3217.2	15.9	3209.9	40.9	109	61	99	0.56	{0.01}
14SA-23@6	1.5584	1.62	0.2576	0.37	22.79	1.66	0.6417	1.62	0.98	-1.4	3232.1	5.8	3218.1	16.3	3195.7	40.9	32	19	29	0.58	{0.01}
14SA-23@7	1.5348	1.61	0.2566	0.21	23.05	1.62	0.6516	1.61	0.99	0.3	3225.9	3.3	3229.1	15.9	3234.3	41.0	101	48	91	0.47	{0.00}
14SA-23@8	1.5416	1.61	0.2558	0.25	22.88	1.63	0.6487	1.61	0.99	0.1	3221.1	4.0	3221.8	16.0	3223.0	41.0	68	36	62	0.53	{0.00}
14SA-23@9	1.4674	1.68	0.2539	0.44	23.86	1.74	0.6815	1.68	0.97	5.6	3209.3	7.0	3262.6	17.1	3350.1	44.1	71	47	69	0.67	{0.01}
14SA-25@01	1.5351	1.62	0.2592	0.39	23.28	1.66	0.6514	1.62	0.97	-0.3	3241.6	6.2	3238.6	16.3	3233.8	41.2	27	18	26	0.66	{0.01}
14SA-25@2	1.5169	1.61	0.2559	0.41	23.26	1.66	0.6592	1.61	0.97	1.7	3221.6	6.5	3237.8	16.3	3264.2	41.5	37	33	36	0.91	{0.00}
14SA-25@3	1.5360	1.62	0.2557	0.30	22.95	1.65	0.6510	1.62	0.98	0.5	3220.3	4.7	3224.9	16.2	3232.2	41.3	47	39	45	0.83	{0.01}
14SA-25@4	1.4832	1.62	0.2569	0.33	23.88	1.65	0.6742	1.62	0.98	3.7	3227.8	5.1	3263.6	16.2	3322.2	42.1	39	37	40	0.93	{0.03}
14SA-25@5	1.5338	1.61	0.2566	0.46	23.07	1.67	0.6520	1.61	0.96	0.4	3226.1	7.2	3229.9	16.4	3235.9	41.0	31	22	30	0.71	{0.03}
14SA-25@6	1.5470	1.62	0.2563	0.33	22.84	1.65	0.6464	1.62	0.98	-0.4	3223.8	5.2	3220.1	16.2	3214.1	41.0	39	34	38	0.88	{0.02}

14SA-25@7	1.5243	1.62	0.2558	0.36	23.14	1.65	0.6561	1.62	0.98	1.2	3221.3	5.6	3233.0	16.2	3251.8	41.4	33	29	32	0.88	{0.01}
**Mount T290**																					
14SA-5-1@2	2.0148	1.62	0.2831	0.66	18.62	1.80	0.4898	1.63	0.91	-27.8	3338.9	11.9	3022.4	17.5	2569.9	34.6	612	255	419	0.42	1.31
14SA-5-1@3	2.5850	2.25	0.3234	0.90	15.10	2.56	0.3682	2.19	0.86	-48.1	3456.8	20.3	2821.4	24.7	2020.7	38.2	695	1004	375	1.44	4.83
14SA-5-1@4	2.1396	1.51	0.3170	0.22	18.28	1.54	0.4487	1.51	0.98	-36.5	3446.6	5.2	3004.6	15.0	2389.5	30.1	438	790	284	1.80	3.99
14SA-5-1@5	1.5068	1.55	0.2984	0.39	27.20	1.60	0.6627	1.55	0.97	-6.6	3458.2	6.1	3390.7	15.8	3277.7	39.9	177	101	168	0.57	0.14
14SA-5-1@6	1.5508	1.51	0.2844	0.13	25.20	1.52	0.6440	1.51	1.00	-6.7	3383.6	2.1	3315.8	14.9	3204.9	38.3	566	333	522	0.59	0.12
14SA-5-1@7	1.4367	1.50	0.2958	0.12	28.14	1.51	0.6939	1.50	1.00	-1.6	3439.4	2.1	3423.9	14.9	3397.3	39.8	364	158	356	0.43	0.31
14SA-16@01	1.5330	1.50	0.2574	0.20	23.15	1.52	0.6523	1.50	0.99	0.3	3230.6	3.1	3233.1	14.9	3237.1	38.4	402	71	342	0.18	0.01
14SA-16@02	1.6373	1.51	0.2599	0.23	21.88	1.52	0.6108	1.51	0.99	-6.7	3245.9	3.5	3178.6	14.9	3073.1	36.9	112	54	95	0.48	{0.01}
14SA-16@03	1.5812	1.52	0.2576	0.17	22.46	1.53	0.6323	1.52	0.99	-2.9	3231.8	2.7	3203.6	15.0	3158.9	38.1	188	112	168	0.60	0.01
14SA-16@04	1.8754	2.07	0.2463	0.66	17.95	2.17	0.5318	2.06	0.95	-15.6	3151.0	10.6	2986.9	21.1	2749.2	46.3	350	70	243	0.20	0.26
14SA-16@05	1.6024	1.51	0.2575	0.31	22.16	1.55	0.6241	1.51	0.98	-4.1	3231.7	5.0	3190.8	15.1	3126.1	37.6	56	27	48	0.48	{0.01}
14SA-16@06	1.6137	1.52	0.2587	0.40	22.11	1.57	0.6197	1.52	0.97	-5.1	3238.9	6.4	3188.4	15.4	3108.7	37.6	81	31	68	0.38	{0.02}
14SA-16@07	1.6092	1.51	0.2477	0.16	21.20	1.52	0.6213	1.51	0.99	-2.1	3168.9	2.6	3147.9	14.9	3115.0	37.5	266	112	224	0.42	0.02
14SA-16@08	1.5633	1.50	0.2560	0.31	22.58	1.53	0.6397	1.50	0.98	-1.4	3222.2	4.9	3208.9	15.0	3187.7	37.9	58	23	51	0.40	{0.02}
14SA-16@09	1.5623	1.51	0.2583	0.21	22.78	1.52	0.6400	1.51	0.99	-1.8	3235.8	3.3	3217.8	14.9	3188.9	38.0	151	31	127	0.21	0.02
14SA-16@10	1.5403	1.51	0.2586	0.21	23.12	1.52	0.6490	1.51	0.99	-0.5	3237.0	3.3	3232.1	14.9	3224.2	38.3	138	58	122	0.42	0.04
14SA-16@11	1.5068	1.56	0.2570	0.25	23.52	1.58	0.6637	1.56	0.99	2.1	3228.3	4.0	3248.5	15.6	3281.4	40.4	182	109	171	0.60	{0.01}
14SA-16@12	1.5506	1.51	0.2573	0.26	22.88	1.53	0.6449	1.51	0.99	-0.9	3230.2	4.1	3221.8	15.0	3208.3	38.3	81	27	70	0.33	{0.01}
14SA-16@13	1.5380	1.50	0.2568	0.22	23.02	1.52	0.6502	1.50	0.99	0.1	3227.3	3.5	3227.9	14.9	3228.9	38.2	154	35	132	0.23	{0.01}
14SA-16@14	1.5376	1.53	0.2574	0.43	23.05	1.59	0.6501	1.53	0.96	0.0	3229.2	6.7	3228.9	15.6	3228.5	38.9	55	20	49	0.37	0.04
14SA-16@15	1.5106	1.56	0.2551	0.28	23.28	1.58	0.6620	1.56	0.98	2.3	3216.6	4.4	3238.9	15.5	3275.0	40.1	187	42	162	0.22	{0.01}
14SA-18-1@01	1.7502	1.51	0.2389	0.16	18.73	1.52	0.5706	1.51	0.99	-7.9	3107.2	2.5	3028.1	14.7	2910.4	35.5	963	23	685	0.02	0.13
14SA-18-1@2	1.5414	1.50	0.2642	0.23	23.63	1.52	0.6488	1.50	0.99	-1.9	3271.9	3.6	3253.3	14.9	3223.4	38.2	127	45	112	0.35	{0.01}
14SA-18-1@3	1.5855	1.54	0.2549	0.13	22.12	1.54	0.6303	1.54	1.00	-2.5	3213.3	2.1	3189.1	15.1	3150.9	38.4	333	7	264	0.02	0.06
14SA-18-1@4	1.5311	1.59	0.2635	0.32	23.71	1.62	0.6530	1.59	0.98	-1.0	3266.8	5.1	3256.6	15.9	3240.0	40.5	118	47	105	0.40	0.02
14SA-18-1@5	1.5644	1.50	0.2612	0.54	22.97	1.60	0.6388	1.50	0.94	-2.6	3251.7	8.5	3225.7	15.7	3184.2	37.8	76	32	66	0.42	0.07
14SA-18-1@6	1.5141	1.52	0.2630	0.24	23.94	1.54	0.6604	1.52	0.99	0.2	3264.0	3.8	3265.8	15.1	3268.6	39.0	250	270	255	1.08	0.01
14SA-18-1@7	7.1167	1.50	0.1572	0.47	2.96	1.58	0.1398	1.50	0.95	-68.8	2387.7	8.3	1398.2	12.1	843.5	11.9	3120	41	509	0.01	0.51
14SA-18-1@8	1.4834	1.51	0.2633	0.21	24.45	1.53	0.6739	1.51	0.99	2.2	3265.5	3.3	3286.4	15.0	3320.9	39.3	162	85	154	0.52	0.03
14SA-21-3@01	1.4872	1.51	0.2979	0.18	27.61	1.52	0.6723	1.51	0.99	-5.3	3459.0	2.8	3405.3	15.0	3314.8	39.1	150	59	141	0.39	0.02
14SA-21-3@2	1.4105	1.51	0.2942	0.34	28.74	1.55	0.7089	1.51	0.98	0.6	3439.3	5.2	3444.7	15.3	3454.2	40.6	122	49	121	0.40	0.02
14SA-21-3@3	1.4539	1.50	0.2955	0.15	28.01	1.51	0.6876	1.50	0.99	-2.7	3446.3	2.3	3419.3	14.9	3373.6	39.5	205	73	196	0.36	0.03
14SA-21-3@4	1.9957	1.51	0.2814	0.34	19.35	1.55	0.5003	1.51	0.98	-27.0	3365.8	5.3	3059.4	15.1	2614.9	32.5	258	95	178	0.37	0.16
14SA-21-3@5	1.4824	1.50	0.2963	0.16	27.55	1.51	0.6745	1.50	0.99	-4.7	3450.7	2.5	3403.2	14.9	3323.1	39.2	218	94	207	0.43	0.02
14SA-21-3@6	1.7935	1.50	0.2919	0.19	22.40	1.51	0.5572	1.50	0.99	-20.6	3426.0	3.0	3201.3	14.8	2855.2	34.7	181	67	140	0.37	0.06
14SA-21-3@7	1.4212	1.53	0.2958	0.24	28.69	1.55	0.7036	1.53	0.99	-0.5	3448.1	3.8	3443.0	15.3	3434.3	40.9	134	49	131	0.36	{0.01}
14SA-21-3@8	1.4538	1.50	0.2963	0.14	28.09	1.51	0.6878	1.50	1.00	-2.8	3450.8	2.2	3422.4	14.9	3374.2	39.6	236	120	232	0.51	0.01
**Mount T120**																					
14SA-20-1@04	1.5751	1.69	0.2924	0.16	25.56	1.69	0.6346	1.69	1.00	-9.6	3428.7	2.4	3329.7	16.7	3167.6	42.4	220	76	189	0.35	0.05
14SA-20-1@02	3.1320	1.75	0.2951	0.28	12.85	1.77	0.3181	1.75	0.99	-54.8	3433.7	4.4	2668.7	16.8	1780.2	27.2	265	60	116	0.23	0.39
14SA-20-1@07	1.4613	1.72	0.2964	0.23	27.93	1.74	0.6839	1.72	0.99	-3.4	3450.1	3.6	3416.5	17.2	3359.5	45.3	410	137	388	0.33	0.06
14SA-20-1@10	3.9520	1.70	0.2515	0.22	6.57	1.77	0.2339	1.71	0.97	-58.0	2855.9	7.4	2055.2	15.7	1354.9	20.9	1156	435	357	0.38	7.56
14SA-20-1@14	1.4465	1.72	0.2970	0.16	28.28	1.73	0.6911	1.72	1.00	-2.5	3453.4	2.5	3428.8	17.1	3386.7	45.5	184	54	175	0.29	0.03
14SA-20-1@17	1.6766	1.70	0.2991	0.26	24.29	1.72	0.5938	1.70	0.99	-16.2	3452.7	4.2	3279.9	16.9	3004.7	41.0	236	94	193	0.40	0.45
14SA-20-1@01	1.5674	2.30	0.3041	2.80	26.70	3.63	0.6376	2.30	0.63	-11.2	3489.5	42.7	3372.6	36.1	3179.5	58.0	270	96	240	0.35	0.07
14SA-20-1@2	1.5661	2.31	0.2954	0.26	25.98	2.32	0.6382	2.31	0.99	-9.7	3445.1	4.0	3345.7	23.0	3182.1	58.2	396	190	352	0.48	0.05
14SA-20-1@3	1.4696	2.36	0.2972	0.17	27.74	2.37	0.6792	2.36	1.00	-4.0	3450.5	2.7	3409.9	23.5	3341.4	61.9	304	120	285	0.39	0.18

**Mount T121**																					
14SA-20-2@01	1.7923	1.56	0.2507	0.24	19.25	1.58	0.5576	1.56	0.99	-12.8	3187.2	3.9	3054.5	15.4	2856.8	36.2	402	14	281	0.04	0.06
14SA-20-2@2	2.2331	1.51	0.2332	0.19	14.34	1.52	0.4473	1.51	0.99	-26.7	3069.5	3.1	2772.5	14.5	2383.2	30.1	321	11	178	0.04	0.12
14SA-20-2@3	1.7466	1.50	0.2483	0.12	19.58	1.51	0.5724	1.50	1.00	-10.0	3173.1	2.0	3071.1	14.6	2917.5	35.3	519	13	372	0.03	0.03
14SA-20-2@4	1.6588	1.51	0.2548	0.14	21.15	1.51	0.6026	1.51	1.00	-6.8	3213.4	2.2	3145.5	14.8	3040.2	36.6	290	20	222	0.07	0.05
14SA-20-2@5	2.8431	1.51	0.2500	0.38	11.93	1.56	0.3501	1.51	0.97	-44.8	3167.2	6.3	2599.2	14.7	1934.9	25.2	76	14	34	0.18	0.47
14SA-20-2@6	1.6815	1.50	0.2527	0.23	20.65	1.52	0.5941	1.50	0.99	-7.5	3198.0	3.6	3122.4	14.8	3006.1	36.2	410	247	344	0.60	0.10
14SA-20-2@09	3.9155	1.55	0.2056	0.34	6.90	1.60	0.2524	1.55	0.96	-53.8	2811.9	7.0	2098.7	14.3	1451.0	20.1	474	355	159	0.75	1.16
14SA-20-2@10	1.6625	1.51	0.2513	0.53	20.66	1.60	0.5999	1.51	0.94	-6.1	3183.4	8.6	3122.9	15.6	3029.6	36.5	70	10	54	0.14	0.26
14SA-20-2@11	2.1179	1.50	0.2486	0.15	15.95	1.51	0.4701	1.50	0.99	-25.7	3159.4	2.6	2873.6	14.5	2484.0	31.0	309	11	181	0.04	0.44
14SA-20-2@12	2.3017	1.50	0.2402	0.21	13.82	1.52	0.4295	1.50	0.99	-29.7	3075.3	3.6	2737.5	14.5	2303.6	29.1	480	49	258	0.10	1.14
14SA-20-2@13	1.6351	1.52	0.2472	0.26	20.83	1.54	0.6114	1.52	0.99	-3.6	3166.2	4.1	3130.7	15.1	3075.8	37.3	510	5	390	0.01	0.02
14SA-20-2@14	1.5269	1.50	0.2940	0.26	26.26	1.53	0.6524	1.50	0.98	-7.1	3427.8	4.2	3356.2	15.0	3237.6	38.3	214	57	189	0.27	0.39
14SA-20-2@15	1.8215	1.54	0.2475	0.12	18.71	1.54	0.5488	1.54	1.00	-13.5	3167.8	2.0	3027.2	15.0	2820.1	35.2	406	8	279	0.02	0.04
14SA-20-2@16	1.5646	1.50	0.2888	0.14	25.37	1.51	0.6384	1.50	1.00	-8.4	3407.9	2.2	3322.5	14.8	3182.7	37.8	245	89	217	0.36	0.12
14SA-20-2@17	6.1849	1.56	0.2522	0.51	5.48	1.65	0.1604	1.56	0.94	-74.7	3170.4	8.8	1897.6	14.3	959.3	13.9	80	28	16	0.35	0.76
14SA-20-2@18	1.9787	1.52	0.2872	0.15	19.97	1.53	0.5050	1.52	1.00	-27.3	3400.6	2.3	3090.0	14.9	2635.3	33.0	393	156	279	0.40	0.07
14SA-20-2@21	1.5960	1.50	0.2552	0.16	22.03	1.51	0.6264	1.50	0.99	-3.2	3216.4	2.5	3185.0	14.8	3135.2	37.4	336	5	264	0.01	0.03
14SA-20-2@22	2.2390	1.67	0.2521	0.29	15.46	1.70	0.4461	1.67	0.99	-30.4	3193.5	4.6	2844.1	16.3	2377.7	33.3	255	95	143	0.37	0.13
14SA-20-2@23	0.7229	2.02	0.2829	0.91	52.04	2.27	1.3666	2.03	0.90	101.2	3341.6	15.7	4032.2	22.8	5553.4	76.0	176	20	327	0.11	1.20
14SA-20-2@24	1.7846	1.61	0.2593	4.39	19.96	4.69	0.5597	1.61	0.34	-14.2	3238.3	67.7	3089.4	46.4	2865.5	37.4	333	4	235	0.01	0.11
14SA-20-2@25	3.5678	1.62	0.2468	0.17	9.28	1.62	0.2780	1.61	0.99	-55.6	3133.0	3.0	2365.6	15.0	1581.4	22.7	658	111	231	0.17	0.81
**Mount T687**																					
16SA-28-1@01	1.5168	1.53	0.2594	0.58	23.58	1.64	0.6593	1.53	0.93	0.8	3242.9	9.2	3251.1	16.1	3264.3	39.3	183	6	153	0.03	{0.01}
16SA-28-1@02	1.8618	1.94	0.2344	0.32	17.27	1.97	0.5363	1.94	0.98	-12.3	3076.3	5.4	2949.8	19.1	2768.0	43.8	170	28	117	0.17	0.15
16SA-28-1@03	2.5289	3.42	0.2212	2.16	11.99	4.05	0.3948	3.41	0.84	-32.9	2982.1	34.6	2603.4	38.7	2145.1	62.6	357	5	173	0.01	0.16
16SA-28-1@04	2.0591	2.85	0.2398	0.36	15.85	2.87	0.4838	2.84	0.99	-21.8	3103.6	5.9	2867.6	27.8	2543.9	60.0	339	80	214	0.24	0.37
16SA-28-1@06	1.6884	1.51	0.2436	0.66	19.87	1.65	0.5921	1.51	0.92	-5.7	3142.2	10.4	3085.0	16.0	2997.9	36.3	396	113	309	0.29	0.03
16SA-28-1@07	1.5750	1.56	0.2612	0.53	22.83	1.65	0.6346	1.56	0.95	-3.3	3252.3	8.4	3219.7	16.2	3167.8	39.2	54	25	47	0.47	0.05
16SA-28-1@08	2.2411	2.21	0.2446	3.41	14.20	4.48	0.4388	2.20	0.49	-28.5	3084.4	60.9	2763.2	43.4	2345.3	43.4	257	6	139	0.02	1.66
16SA-28-1@09	1.6132	1.69	0.2610	0.20	22.29	1.70	0.6197	1.69	0.99	-5.5	3251.8	3.1	3196.3	16.7	3108.8	41.8	181	9	143	0.05	0.03
16SA-28-1@10	1.6537	1.50	0.2684	2.20	19.49	3.63	0.5796	1.58	0.43	-7.8	3145.2	51.0	3066.2	35.7	2947.2	37.4	32	1	28	0.04	4.15
16SA-28-1@11	1.5367	1.51	0.2606	0.40	23.36	1.56	0.6505	1.51	0.97	-0.7	3249.1	6.3	3241.9	15.3	3230.2	38.4	46	16	40	0.35	0.04
16SA-28-1@12	1.6299	1.50	0.2622	0.55	22.17	1.60	0.6134	1.50	0.94	-6.8	3259.4	8.6	3191.0	15.7	3083.5	37.0	39	17	33	0.43	* 0.02
16SA-28-1@13	1.5328	2.29	0.2549	0.81	22.93	2.43	0.6524	2.29	0.94	0.9	3215.3	12.7	3223.8	23.9	3237.6	58.5	234	50	200	0.21	{0.03}
16SA-28-1@14	1.5479	2.38	0.2594	0.94	23.06	2.56	0.6456	2.38	0.93	-1.2	3240.7	14.7	3229.3	25.2	3211.0	60.5	118	71	108	0.60	0.07
16SA-28-1@15	1.5728	1.75	0.2612	0.25	22.82	1.77	0.6352	1.75	0.99	-3.1	3250.5	4.0	3219.5	17.3	3169.9	43.9	61	30	54	0.49	0.10
16SA-29@01	1.5578	1.56	0.2596	0.18	22.96	1.57	0.6418	1.56	0.99	-1.9	3243.7	2.9	3225.4	15.4	3196.1	39.4	117	53	103	0.46	0.02
16SA-29@02	1.7889	1.89	0.2909	2.34	18.24	4.24	0.5227	1.81	0.43	-18.8	3204.2	59.4	3002.6	41.7	2710.8	40.1	1	0	[0]	0.04	6.49
16SA-29@03	1.5173	1.58	0.2610	0.32	23.72	1.61	0.6591	1.58	0.98	0.4	3252.7	5.0	3256.8	15.8	3263.5	40.6	37	12	33	0.32	{0.24}
16SA-29@04	1.5359	1.67	0.2575	0.24	23.12	1.68	0.6511	1.67	0.99	0.0	3231.6	3.8	3231.9	16.5	3232.5	42.5	64	16	55	0.26	{0.07}
16SA-29@05	1.5103	1.53	0.2589	0.39	23.63	1.58	0.6621	1.53	0.97	1.4	3239.7	6.2	3253.3	15.5	3275.4	39.4	72	27	65	0.38	{0.03}
16SA-29@06	1.6160	1.53	0.2591	0.42	22.07	1.59	0.6185	1.53	0.96	-5.3	3239.3	6.7	3186.8	15.5	3104.0	37.8	118	7	93	0.06	0.05
16SA-29@07	1.5808	1.51	0.2630	0.56	22.92	1.61	0.6324	1.51	0.94	-4.1	3264.0	8.7	3223.7	15.8	3159.2	37.9	118	58	104	0.49	0.02
16SA-29@08	1.5517	1.57	0.2550	0.18	22.64	1.58	0.6443	1.57	0.99	-0.4	3214.9	2.8	3211.4	15.5	3205.9	39.8	187	2	151	0.01	0.03
16SA-29@09	1.5115	1.52	0.2589	0.38	23.61	1.57	0.6616	1.52	0.97	1.3	3239.9	6.0	3252.6	15.4	3273.3	39.2	28	10	25	0.34	{0.04}
16SA-29@10	1.5700	1.50	0.2652	0.40	23.26	1.56	0.6366	1.50	0.97	-3.9	3276.4	6.3	3237.8	15.3	3175.8	37.8	41	18	36	0.43	0.05
16SA-29@11	2.1187	2.00	0.2593	0.31	16.80	2.02	0.4714	2.00	0.99	-27.8	3237.8	4.9	2923.7	19.6	2489.6	41.4	106	30	68	0.28	0.13
16SA-29@12	1.5280	1.55	0.2604	0.41	23.48	1.61	0.6543	1.55	0.97	-0.1	3248.3	6.5	3247.0	15.8	3244.9	39.7	38	11	34	0.30	* 0.03

16SA-29@13	1.5512	1.91	0.2615	0.49	23.22	1.97	0.6445	1.91	0.97	-1.9	3254.6	7.7	3236.2	19.3	3206.5	48.3	61	25	54	0.41	0.03
16SA-29@14	1.9339	1.78	0.2624	0.57	18.63	1.86	0.5165	1.78	0.95	-21.4	3256.9	8.9	3023.1	18.1	2684.2	39.1	140	16	94	0.11	0.12
16SA-29@15	1.5762	1.50	0.2624	0.33	22.94	1.54	0.6343	1.50	0.98	-3.6	3260.3	5.1	3224.3	15.0	3166.7	37.6	78	34	68	0.44	* 0.02
16SA-29@16	1.6161	1.77	0.2574	1.10	21.87	2.09	0.6180	1.77	0.85	-4.9	3226.4	17.4	3178.1	20.5	3102.0	43.7	55	19	46	0.34	0.12
16SA-29@17	1.6000	3.45	0.2655	1.60	22.82	3.81	0.6245	3.45	0.91	-5.7	3276.8	25.0	3219.3	37.7	3127.8	86.1	72	22	60	0.30	0.08
16SA-29@18	1.5514	1.66	0.2687	0.84	22.68	1.88	0.6342	1.65	0.88	-3.0	3243.2	14.1	3213.4	18.5	3166.0	41.5	40	12	34	0.30	1.62
16SA-29@19	1.4911	1.50	0.2587	0.37	23.92	1.55	0.6706	1.50	0.97	2.7	3238.8	5.8	3265.2	15.2	3308.3	39.0	26	9	23	0.34	{0.05}
16SA-29@20	1.4476	1.56	0.2601	0.32	24.78	1.59	0.6908	1.56	0.98	5.5	3247.6	5.0	3299.5	15.7	3385.6	41.2	66	23	63	0.35	{0.80}
16SA-29@21	1.5305	2.12	0.2580	0.85	23.24	2.29	0.6534	2.12	0.93	0.3	3234.6	13.4	3237.2	22.5	3241.3	54.3	107	38	94	0.35	{0.02}
16SA-31-1@01	1.4654	1.50	0.2635	0.54	24.79	1.59	0.6824	1.50	0.94	3.4	3267.7	8.4	3300.1	15.7	3353.6	39.4	90	47	87	0.52	{0.95}
16SA-31-1@02	1.7466	1.92	0.2614	0.32	20.54	1.95	0.5717	1.92	0.99	-12.8	3250.0	5.0	3117.0	19.0	2914.8	45.2	249	92	193	0.37	0.15
16SA-31-1@03	1.5891	1.54	0.2586	0.70	22.41	1.69	0.6290	1.54	0.91	-3.5	3236.7	11.0	3201.6	16.6	3145.8	38.5	133	53	114	0.40	0.04
16SA-31-1@04	1.5933	3.30	0.2611	0.77	22.58	3.39	0.6275	3.30	0.97	-4.4	3252.4	12.1	3208.9	33.5	3139.6	82.5	120	24	99	0.20	0.02
16SA-31-1@05	1.5568	1.50	0.2584	0.27	22.86	1.53	0.6421	1.50	0.98	-1.5	3235.6	4.2	3220.9	15.0	3197.3	38.0	198	59	170	0.30	0.03
16SA-31-1@06	1.3827	8.70	0.2595	0.46	25.88	8.71	0.7232	8.70	1.00	10.6	3243.7	7.2	3341.9	89.0	3508.2	239.9	99	41	98	0.41	{0.03}
16SA-31-1@07	1.5797	2.26	0.2601	0.45	22.61	2.31	0.6323	2.26	0.98	-3.3	3242.8	7.1	3210.3	22.7	3158.5	56.7	385	157	329	0.41	0.12
16SA-31-1@08	1.5539	1.54	0.2602	0.22	23.06	1.56	0.6432	1.54	0.99	-1.7	3246.5	3.5	3229.3	15.3	3201.8	39.0	233	147	213	0.63	0.04
16SA-31-1@09	1.6311	1.50	0.2561	1.17	21.60	1.90	0.6127	1.50	0.79	-5.4	3220.4	18.4	3166.0	18.6	3080.8	36.8	234	66	190	0.28	0.06
16SA-31-1@10	1.5239	1.60	0.2586	0.32	23.39	1.63	0.6562	1.60	0.98	0.6	3238.0	5.1	3243.5	16.0	3252.4	41.0	139	94	131	0.68	{0.02}
16SA-31-1@11	1.6108	1.62	0.2612	0.80	22.31	1.81	0.6204	1.62	0.90	-5.4	3251.6	12.5	3197.3	17.7	3111.4	40.1	207	64	173	0.31	0.07
16SA-31-2@02	1.6449	3.36	0.2647	1.72	22.14	3.78	0.6076	3.36	0.89	-8.1	3272.7	26.8	3190.0	37.4	3060.3	82.5	116	38	95	0.33	0.06
16SA-31-2@03	1.5061	1.71	0.2586	0.71	23.68	1.85	0.6640	1.71	0.92	1.7	3238.4	11.1	3255.2	18.2	3282.5	44.0	41	17	37	0.41	{0.05}
16SA-31-2@04	1.5083	1.60	0.2589	0.38	23.67	1.65	0.6630	1.60	0.97	1.5	3240.1	5.9	3254.8	16.2	3278.9	41.4	100	48	92	0.48	{0.04}
16SA-31-2@05	1.5078	1.50	0.2569	0.55	23.49	1.60	0.6632	1.50	0.94	2.1	3227.6	8.6	3247.4	15.7	3279.7	38.8	187	64	165	0.34	{0.04}
16SA-31-2@06	1.5830	1.59	0.2620	0.41	22.79	1.65	0.6314	1.59	0.97	-4.0	3257.3	6.5	3218.0	16.2	3155.1	39.9	66	39	59	0.59	0.05
16SA-31-2@07	1.6404	2.89	0.2617	1.13	21.84	3.10	0.6083	2.88	0.93	-7.2	3249.3	17.8	3176.7	30.5	3063.0	70.6	19	0	14	0.01	0.22
16SA-31-2@08	1.5602	1.50	0.2591	0.43	22.85	1.56	0.6404	1.50	0.96	-1.9	3238.9	6.8	3220.4	15.3	3190.8	37.9	31	10	26	0.33	0.08
16SA-31-2@09	1.6097	1.61	0.2613	0.32	22.36	1.64	0.6211	1.61	0.98	-5.4	3253.6	5.0	3199.6	16.1	3114.1	39.8	91	44	79	0.49	0.03
16SA-31-2@10	1.5819	1.52	0.2618	0.57	22.79	1.62	0.6320	1.52	0.94	-3.8	3256.3	9.0	3218.2	15.9	3157.4	38.1	69	38	61	0.55	0.03
16SA-31-2@11	1.5316	1.62	0.2588	0.42	23.28	1.67	0.6527	1.62	0.97	0.0	3238.6	6.6	3238.7	16.4	3238.9	41.3	177	74	158	0.42	0.03
16SA-31-2@12	1.5810	1.50	0.2624	0.35	22.85	1.54	0.6322	1.50	0.97	-3.9	3259.6	5.5	3220.5	15.1	3158.1	37.6	197	116	176	0.59	0.06
16SA-31-2@13	1.5564	1.58	0.2596	0.23	22.96	1.60	0.6422	1.58	0.99	-1.7	3242.4	3.7	3225.3	15.7	3197.8	40.0	106	49	94	0.46	0.05
16SA-31-2@14	1.5381	1.55	0.2597	0.39	23.22	1.60	0.6496	1.55	0.97	-0.6	3242.1	6.2	3236.2	15.7	3226.8	39.5	54	13	46	0.25	0.08
16SA-31-2@15	1.5713	1.73	0.2613	0.18	22.91	1.74	0.6363	1.73	0.99	-3.1	3253.5	2.8	3223.0	17.1	3174.3	43.6	184	82	161	0.45	0.02
**Mount T688**																					
16SA-18-2@01	1.6797	1.50	0.2507	1.36	19.36	2.13	0.5847	1.50	0.71	-6.1	3120.8	23.8	3060.0	20.8	2968.1	35.9	21	1	16	0.03	1.78
16SA-18-2@02	2.1621	2.15	0.2224	0.45	13.68	2.18	0.4581	2.13	0.97	-21.2	2955.0	7.9	2727.6	20.9	2431.3	43.2	826	85	467	0.10	0.95
16SA-18-2@03	1.6620	1.86	0.2515	0.68	20.80	1.99	0.6012	1.86	0.94	-6.1	3190.9	10.8	3129.5	19.4	3034.6	45.2	95	24	76	0.26	0.09
16SA-18-2@04	1.8183	1.62	0.2729	0.38	16.05	1.80	0.5096	1.57	0.87	-15.4	3040.7	14.2	2879.7	17.4	2655.1	34.2	107	38	70	0.36	7.33
16SA-18-2@05	1.7797	1.55	0.2164	0.65	16.34	1.69	0.5582	1.55	0.92	-2.7	2922.9	10.9	2896.7	16.3	2859.1	35.9	167	8	114	0.05	0.66
16SA-18-2@06	1.5426	1.58	0.2514	0.88	22.47	1.81	0.6482	1.58	0.87	1.1	3193.9	13.9	3204.4	17.8	3221.3	40.2	344	312	331	0.91	{0.12}
16SA-18-2@07	1.4003	1.58	0.2876	0.56	28.32	1.68	0.7141	1.58	0.94	2.6	3404.8	8.6	3430.2	16.6	3474.0	42.6	388	117	379	0.30	{0.19}
16SA-18-2@08	2.1967	1.62	0.2533	0.59	15.07	1.75	0.4480	1.61	0.92	-28.8	3145.8	11.0	2819.5	16.8	2386.3	32.2	105	25	59	0.24	1.59
16SA-18-2@09	1.4759	1.81	0.2476	0.28	23.13	1.84	0.6775	1.81	0.99	6.7	3169.4	4.4	3232.4	18.0	3334.9	47.4	303	32	262	0.10	{0.19}
16SA-18-2@10	1.5077	1.60	0.2528	0.54	23.11	1.68	0.6632	1.60	0.95	3.1	3202.1	8.4	3231.7	16.5	3279.7	41.2	62	2	52	0.04	{0.87}
16SA-18-2@11	1.5407	1.51	0.2471	0.42	22.11	1.57	0.6490	1.51	0.96	2.3	3166.3	6.7	3188.7	15.4	3224.5	38.4	46	3	38	0.07	{0.06}
16SA-18-2@12	1.3532	2.26	0.2519	1.12	25.66	2.52	0.7390	2.26	0.90	15.1	3196.6	17.6	3333.8	24.9	3566.8	62.1	187	95	196	0.51	{1.13}
16SA-18-2@13	2.4594	3.02	0.2084	0.70	11.16	3.12	0.4020	3.02	0.97	-27.3	2837.0	12.7	2536.6	29.5	2178.4	56.1	593	29	292	0.05	1.12
16SA-18-2@14	3.4890	2.63	0.1944	1.89	7.41	3.32	0.2843	2.61	0.79	-46.2	2733.7	33.4	2162.0	30.2	1612.7	37.4	1396	30	476	0.02	0.83

16SA-18-2@15	1.5278	1.57	0.2455	0.59	22.16	1.67	0.6545	1.57	0.94	3.6	3156.3	9.3	3190.8	16.4	3245.9	40.1	366	260	342	0.71	{0.13}
16SA-18-2@16	1.7026	1.54	0.2607	0.94	21.03	1.81	0.5866	1.54	0.85	-10.4	3246.8	14.8	3140.0	17.7	2975.7	36.7	188	232	177	1.23	0.12
16SA-18-2@17	1.5297	2.00	0.2542	0.30	22.91	2.02	0.6537	2.00	0.99	1.2	3211.3	4.7	3223.3	19.9	3242.7	51.2	97	56	89	0.58	{0.26}
16SA-18-2@18	2.7352	2.16	0.2975	0.87	11.66	2.78	0.3367	1.94	0.70	-47.5	3192.8	31.2	2577.8	26.3	1870.6	31.5	223	47	101	0.21	7.92
16SA-18-2@20	2.1118	4.01	0.2440	0.43	14.75	4.03	0.4633	3.99	0.99	-23.7	3058.6	8.3	2799.4	39.0	2454.0	82.0	213	21	124	0.10	2.16
16SA-18-3@01	1.3333	1.60	0.3473	0.79	26.25	2.06	0.6661	1.58	0.77	-3.9	3394.9	20.5	3355.9	20.4	3290.9	40.8	395	127	352	0.32	11.18
16SA-18-3@02	1.4174	1.52	0.2923	0.33	28.43	1.55	0.7055	1.52	0.98	0.4	3429.8	5.1	3434.1	15.3	3441.6	40.5	346	96	334	0.28	{0.02}
16SA-18-3@03	2.2952	2.74	0.3797	2.13	15.84	3.99	0.3752	2.44	0.61	-48.0	3501.5	47.9	2866.9	38.9	2053.6	43.1	550	241	288	0.44	13.89
16SA-18-3@04	1.4523	1.54	0.2927	1.28	27.31	2.02	0.6844	1.54	0.76	-2.0	3414.6	20.3	3394.7	20.0	3361.2	40.5	304	96	285	0.32	0.60
16SA-18-3@05	1.9464	2.47	0.2649	0.69	18.36	2.55	0.5102	2.44	0.96	-22.2	3252.4	11.7	3008.6	24.9	2657.6	53.4	879	307	608	0.35	0.69
16SA-18-3@06	1.6627	1.61	0.2813	0.18	23.21	1.62	0.6004	1.61	0.99	-12.4	3365.1	2.8	3235.9	15.9	3031.5	39.1	608	245	504	0.40	0.17
16SA-18-3@07	1.4552	2.77	0.2909	0.27	27.54	2.79	0.6870	2.77	1.00	-1.9	3421.6	4.2	3402.9	27.7	3371.3	73.2	435	201	420	0.46	0.03
16SA-18-3@08	1.5270	1.63	0.2966	0.48	24.89	1.75	0.6384	1.63	0.93	-7.3	3378.2	9.8	3303.9	17.2	3182.8	41.1	430	203	378	0.47	2.51
16SA-18-3@09	1.6163	2.04	0.3002	0.92	25.33	2.24	0.6162	2.03	0.91	-13.3	3460.3	14.4	3320.9	22.1	3094.9	50.1	640	378	572	0.59	0.40
16SA-18-3@10	1.4535	2.42	0.2909	0.29	27.57	2.44	0.6877	2.42	0.99	-1.8	3421.6	4.5	3403.9	24.2	3374.0	63.8	275	78	257	0.28	0.04
16SA-18-3@11	1.4319	1.55	0.3034	0.69	27.05	1.75	0.6796	1.53	0.87	-2.5	3410.6	13.3	3385.4	17.3	3342.9	40.1	425	161	401	0.38	2.69
16SA-18-3@12	7.0931	11.18	0.3290	2.43	2.91	14.35	0.1107	8.36	0.58	-79.2	2747.3	179.9	1384.5	114.7	677.0	54.0	3373	1330	463	0.39	21.46
16SA-20-2@01	1.5748	1.91	0.2605	0.77	21.83	2.09	0.6264	1.90	0.91	-2.6	3201.7	13.6	3176.0	20.5	3135.5	47.3	26	4	21	0.14	1.35
16SA-20-2@02	1.4848	1.50	0.2475	0.30	22.98	1.53	0.6735	1.50	0.98	6.1	3168.8	4.8	3226.2	15.0	3319.3	39.1	83	6	71	0.07	{0.06}
16SA-20-2@03	1.6265	2.13	0.2568	0.40	21.72	2.17	0.6144	2.13	0.98	-5.4	3224.7	6.3	3171.3	21.3	3087.5	52.5	117	7	91	0.06	0.07
16SA-20-2@04	1.7007	1.74	0.2529	0.68	20.43	1.87	0.5874	1.74	0.93	-8.6	3199.1	10.7	3112.0	18.2	2978.8	41.6	345	76	265	0.22	0.10
16SA-20-2@05	1.4726	1.52	0.2480	0.19	23.22	1.53	0.6791	1.52	0.99	6.8	3172.3	3.1	3236.4	15.0	3340.8	39.7	195	54	174	0.28	{0.10}
16SA-20-2@06	1.7759	3.90	0.2693	2.16	20.80	4.46	0.5621	3.89	0.87	-15.8	3296.2	33.7	3129.1	44.2	2875.4	91.0	71	9	52	0.13	0.17
16SA-20-2@07	1.5447	1.61	0.2567	0.42	22.88	1.66	0.6471	1.61	0.97	-0.3	3225.0	6.6	3221.8	16.3	3216.7	40.9	329	61	277	0.19	0.05
16SA-20-2@08	1.3702	1.64	0.3083	0.29	31.02	1.66	0.7298	1.64	0.98	0.8	3512.2	4.5	3519.6	16.5	3532.7	44.7	127	106	141	0.83	{0.04}
16SA-20-2@09	1.6214	1.56	0.2594	0.25	21.99	1.58	0.6161	1.56	0.99	-5.6	3239.5	4.0	3183.1	15.4	3094.5	38.4	149	22	119	0.15	0.10
16SA-20-2@10	1.7396	2.03	0.2510	1.27	19.67	2.41	0.5729	2.02	0.84	-10.1	3178.7	20.6	3075.4	23.6	2919.8	47.6	379	6	272	0.01	0.34
16SA-20-2@11	1.5045	1.70	0.2529	0.35	23.18	1.74	0.6647	1.70	0.98	3.3	3203.2	5.6	3234.5	17.1	3285.2	43.9	160	77	146	0.48	{0.04}
16SA-20-2@12	1.5744	2.39	0.2616	0.96	22.85	2.58	0.6346	2.39	0.93	-3.3	3253.3	15.1	3220.4	25.4	3167.8	60.1	84	35	73	0.42	0.08
16SA-20-2@14-1	1.6098	1.52	0.2618	0.13	22.36	1.52	0.6207	1.52	1.00	-5.5	3254.3	2.1	3199.4	14.9	3112.5	37.6	500	60	398	0.12	0.08
**Mount T690**																					
16SA-9-2@01	1.6193	1.52	0.2590	0.26	22.01	1.54	0.6172	1.52	0.99	-5.4	3238.3	4.1	3184.0	15.1	3098.7	37.4	191	9	150	0.05	0.06
16SA-9-2@02	2.3504	1.67	0.2121	0.94	12.36	1.92	0.4248	1.67	0.87	-25.7	2913.8	15.3	2632.3	18.2	2282.1	32.1	673	642	415	0.95	0.16
16SA-9-2@03	1.5904	1.51	0.2571	0.18	22.09	1.52	0.6270	1.51	0.99	-3.2	3219.0	3.2	3187.5	14.9	3137.8	37.6	252	44	206	0.17	0.28
16SA-9-2@04	1.5416	1.50	0.2597	0.36	22.88	1.56	0.6457	1.50	0.97	-0.7	3228.2	6.3	3221.6	15.3	3211.2	38.2	100	2	81	0.02	0.47
16SA-9-2@05	1.7129	1.73	0.2656	0.23	20.79	1.80	0.5787	1.72	0.96	-11.7	3250.0	8.0	3128.9	17.5	2943.6	40.8	278	86	208	0.31	0.87
16SA-9-2@06	1.6317	1.64	0.2630	1.20	21.90	2.04	0.6100	1.63	0.80	-6.9	3248.8	19.1	3179.2	20.0	3070.1	40.0	532	463	489	0.87	0.46
16SA-9-2@07	1.6352	1.60	0.2640	0.71	21.71	1.78	0.6068	1.60	0.90	-7.2	3243.8	12.3	3171.0	17.4	3057.0	39.0	247	26	193	0.10	0.78
16SA-9-2@08	1.6792	1.52	0.2503	0.24	20.45	1.54	0.5946	1.52	0.99	-6.8	3180.9	3.9	3112.7	15.0	3007.9	36.5	367	61	282	0.17	0.16
16SA-9-2@09	1.8330	1.51	0.2388	0.37	17.91	1.56	0.5451	1.51	0.97	-12.0	3108.5	5.8	2984.8	15.1	2804.6	34.5	598	560	481	0.94	0.09
16SA-9-2@10	1.7503	3.47	0.2618	0.46	20.38	3.49	0.5692	3.46	0.99	-13.0	3244.4	7.8	3109.4	34.4	2904.6	81.4	186	6	136	0.03	0.37
16SA-9-2@11	2.7443	3.10	0.2100	1.77	10.47	3.57	0.3637	3.09	0.87	-35.9	2895.7	28.7	2477.0	33.7	1999.5	53.4	930	905	496	0.97	0.20
16SA-9-2@13	1.6966	4.54	0.2824	1.92	22.84	4.92	0.5884	4.53	0.92	-14.3	3371.1	29.8	3220.0	49.1	2983.1	109.1	656	580	571	0.88	0.16
16SA-9-2@14	2.5851	7.20	0.2017	3.29	10.65	7.91	0.3859	7.18	0.91	-29.9	2827.4	53.2	2493.1	76.2	2103.8	130.2	592	6	275	0.01	0.24
16SA-10-1@01	1.4101	1.72	0.2984	0.18	29.16	1.73	0.7090	1.72	0.99	-0.3	3461.4	2.8	3458.9	17.2	3454.5	46.2	130	51	128	0.39	0.03
16SA-10-1@02	1.4293	1.50	0.2980	0.23	28.72	1.52	0.6994	1.50	0.99	-1.5	3459.0	3.6	3444.1	15.0	3418.5	40.0	148	56	144	0.38	0.03
16SA-10-1@03	1.4473	1.56	0.2997	0.31	28.53	1.60	0.6907	1.56	0.98	-3.0	3467.7	4.9	3437.3	15.8	3385.4	41.3	228	90	220	0.39	0.03
16SA-10-1@04	1.4599	1.61	0.2997	0.29	28.28	1.63	0.6848	1.61	0.98	-3.9	3467.8	4.5	3428.9	16.2	3362.7	42.3	242	150	242	0.62	0.03
16SA-10-1@05	1.6375	1.63	0.3013	0.36	25.29	1.67	0.6100	1.63	0.98	-14.6	3473.6	5.6	3319.4	16.4	3070.0	40.0	172	60	147	0.35	0.11

16SA-10-1@06	1.4779	2.67	0.3098	1.00	28.27	2.85	0.6711	2.65	0.93	-6.9	3498.3	15.9	3428.5	28.3	3310.2	69.1	190	76	179	0.40	0.82
16SA-10-1@07	1.4409	1.50	0.2969	0.41	28.36	1.56	0.6935	1.50	0.96	-2.1	3452.3	6.3	3431.6	15.4	3396.1	39.7	157	58	151	0.37	0.07
16SA-10-1@08	1.4864	1.57	0.2996	0.27	27.58	1.59	0.6709	1.56	0.98	-5.6	3460.6	4.2	3404.3	15.7	3309.5	40.6	135	51	126	0.38	0.27
16SA-10-1@09	1.3829	1.51	0.2962	0.74	29.53	1.68	0.7231	1.51	0.90	2.1	3450.5	11.4	3471.4	16.7	3507.6	41.0	175	53	174	0.30	{0.03}
16SA-10-1@10	1.4235	1.52	0.2988	0.25	28.92	1.54	0.7023	1.52	0.99	-1.2	3463.0	3.8	3450.7	15.2	3429.5	40.6	197	80	194	0.40	0.03
16SA-10-1@11	3.3491	11.53	0.2280	4.85	6.93	13.28	0.2772	10.6	0.80	-45.8	2663.8	126.1	2102.0	125.3	1577.4	150.5	1712	469	581	0.27	7.15
16SA-10-1@12	2.7204	1.53	0.2580	0.43	11.83	1.61	0.3568	1.53	0.95	-42.8	3123.0	7.7	2590.9	15.2	1966.8	26.0	906	285	416	0.31	2.95
16SA-10-1@13	2.5156	1.50	0.2644	0.32	13.37	1.55	0.3878	1.50	0.97	-39.3	3185.5	5.9	2706.4	14.7	2112.6	27.1	708	253	360	0.36	2.44
16SA-10-1@14	1.4872	1.56	0.2936	0.19	27.13	1.57	0.6716	1.56	0.99	-4.5	3433.5	3.0	3388.2	15.5	3312.0	40.6	129	46	120	0.36	0.12
16SA-11@01	1.4266	1.60	0.2985	0.31	28.82	1.63	0.7007	1.60	0.98	-1.4	3461.4	4.8	3447.4	16.1	3423.5	42.6	164	59	160	0.36	0.03
16SA-11@02	1.7952	3.68	0.2937	1.00	22.41	3.80	0.5557	3.67	0.96	-20.9	3430.5	15.6	3201.5	37.6	2848.8	85.0	395	159	307	0.40	0.24
16SA-11@03	1.4195	1.51	0.2975	0.23	28.87	1.53	0.7043	1.51	0.99	-0.7	3456.4	3.5	3449.2	15.1	3437.0	40.4	137	40	132	0.29	0.02
16SA-11@04	1.4797	1.53	0.2944	0.24	27.39	1.55	0.6755	1.53	0.99	-4.2	3439.6	3.7	3397.7	15.3	3327.0	39.8	190	72	178	0.38	0.05
16SA-11@05	1.4529	1.54	0.2940	0.26	27.83	1.56	0.6877	1.54	0.99	-2.3	3436.1	4.1	3413.0	15.4	3373.8	40.5	149	61	143	0.41	0.09
16SA-11@06	1.3776	1.50	0.2973	0.23	29.75	1.52	0.7259	1.50	0.99	2.3	3456.1	3.6	3478.7	15.0	3518.1	40.8	161	61	164	0.38	{0.93}
16SA-11@07	1.4887	1.73	0.2949	0.69	27.26	1.86	0.6713	1.73	0.93	-4.8	3441.6	10.6	3392.9	18.4	3311.0	44.9	329	146	311	0.44	0.06
16SA-11@08	1.6024	1.77	0.2991	0.17	25.58	1.78	0.6227	1.77	0.99	-12.3	3459.3	3.0	3330.5	17.6	3120.6	43.9	338	134	296	0.39	0.22
16SA-11@09	1.4681	1.83	0.3001	0.52	28.15	1.90	0.6809	1.83	0.96	-4.5	3469.6	8.0	3424.4	18.8	3347.6	48.0	115	30	107	0.26	0.05
16SA-11@10	1.9912	1.53	0.2891	0.20	19.88	1.55	0.5010	1.53	0.99	-28.0	3405.5	3.3	3085.4	15.1	2618.1	33.1	146	56	99	0.39	0.24
16SA-11@11	1.5015	1.51	0.2981	0.21	27.25	1.52	0.6649	1.51	0.99	-6.3	3455.7	3.2	3392.4	15.0	3286.3	39.0	198	72	183	0.36	0.16
16SA-11@12	1.4618	1.79	0.2996	0.35	28.21	1.83	0.6836	1.79	0.98	-4.0	3466.4	5.5	3426.4	18.1	3358.2	47.1	172	61	162	0.36	0.07
**Mount T692**																					
16SA-1@01	1.6153	1.52	0.2384	0.19	20.34	1.53	0.6190	1.52	0.99	-0.1	3108.9	3.1	3107.7	14.9	3105.8	37.5	140	96	123	0.69	0.02
16SA-1@02	1.6933	1.60	0.2427	1.06	19.71	1.92	0.5901	1.60	0.83	-5.8	3134.8	16.9	3077.3	18.7	2989.9	38.3	162	159	144	0.98	0.07
16SA-1@03	1.5965	1.63	0.2374	0.22	20.50	1.64	0.6264	1.63	0.99	1.3	3102.4	3.4	3115.2	16.0	3135.2	40.5	111	108	104	0.97	{0.03}
16SA-1@04	1.6719	1.63	0.2400	0.28	19.62	1.66	0.5966	1.63	0.98	-3.8	3110.0	4.7	3072.8	16.1	3016.2	39.4	150	99	127	0.66	0.25
16SA-1@05	2.3101	2.08	0.2305	0.67	12.87	2.59	0.4252	2.07	0.80	-27.6	2977.4	24.9	2670.3	24.7	2284.0	40.0	335	246	203	0.74	1.78
16SA-1@06	1.6076	1.55	0.2384	0.19	20.45	1.56	0.6220	1.55	0.99	0.3	3109.4	3.1	3112.8	15.2	3118.0	38.3	137	111	124	0.81	{0.04}
16SA-1@07	1.6264	1.55	0.2392	0.21	20.26	1.56	0.6147	1.55	0.99	-1.0	3113.9	3.3	3104.0	15.2	3088.9	38.1	135	93	118	0.69	0.02
16SA-1@08	2.6814	4.91	0.2325	0.83	11.15	4.93	0.3660	4.80	0.97	-37.9	2988.2	17.8	2536.0	47.0	2010.5	83.5	344	144	173	0.42	1.87
16SA-1@09	1.6451	1.50	0.2397	0.21	19.98	1.52	0.6069	1.50	0.99	-2.2	3111.8	3.5	3090.4	14.8	3057.7	36.7	116	83	101	0.72	0.15
16SA-1@10	1.5721	1.59	0.2373	0.30	20.81	1.62	0.6361	1.59	0.98	2.9	3101.6	4.8	3129.7	15.8	3173.6	40.1	308	181	271	0.59	{0.01}
16SA-1@11	3.4698	5.54	0.2153	3.12	6.86	6.67	0.2735	5.17	0.78	-46.7	2669.7	68.2	2093.0	60.9	1558.3	72.0	434	174	155	0.40	5.12
16SA-1@12	1.6147	1.50	0.2367	0.21	20.21	1.52	0.6193	1.50	0.99	0.4	3098.0	3.4	3101.6	14.8	3107.2	37.1	115	90	103	0.79	{0.03}
16SA-3-4@01	1.7057	4.02	0.2975	0.48	23.70	4.03	0.5833	4.00	0.99	-17.4	3442.8	7.8	3256.3	40.0	2962.0	95.6	89	30	73	0.34	0.51
16SA-3-4@02	1.4267	1.54	0.2926	0.36	28.26	1.58	0.7007	1.54	0.97	-0.3	3430.8	5.7	3428.1	15.7	3423.5	41.1	173	48	166	0.28	0.03
16SA-3-4@03	1.6583	1.50	0.2823	0.50	23.31	1.59	0.6016	1.50	0.95	-12.3	3368.8	7.9	3240.2	15.6	3036.4	36.5	270	94	221	0.35	0.23
16SA-3-4@04	1.5726	3.83	0.2927	0.48	25.56	3.86	0.6350	3.83	0.99	-9.5	3427.9	7.6	3329.8	38.4	3169.2	96.5	195	96	176	0.49	0.14
16SA-3-4@05	1.6519	1.52	0.2845	0.22	23.56	1.53	0.6037	1.52	0.99	-12.4	3379.7	3.6	3250.3	15.0	3044.9	36.9	436	145	359	0.33	0.27
16SA-3-4@06	2.4092	3.23	0.2117	2.82	11.64	4.34	0.4109	3.20	0.74	-26.7	2869.4	47.0	2575.6	41.4	2219.1	60.3	537	20	276	0.04	1.01
16SA-3-4@07	1.3915	1.51	0.2933	0.31	29.06	1.54	0.7186	1.51	0.98	2.1	3434.9	4.8	3455.4	15.3	3490.9	40.9	124	41	123	0.33	{0.04}
16SA-3-4@08	2.8602	1.96	0.2066	1.36	9.60	2.41	0.3465	1.95	0.81	-37.2	2833.9	22.9	2397.1	22.4	1917.8	32.4	922	52	391	0.06	0.90
16SA-3-4@09	1.4885	1.52	0.2917	0.20	26.89	1.53	0.6706	1.52	0.99	-4.2	3421.7	3.2	3379.3	15.1	3308.2	39.4	244	101	229	0.41	0.18
16SA-3-4@10	1.6024	2.12	0.2953	1.05	25.29	2.37	0.6230	2.12	0.90	-11.7	3440.8	16.3	3319.4	23.4	3122.0	52.6	160	53	138	0.33	0.16
16SA-3-4@11	2.5239	1.52	0.2252	2.70	11.64	3.24	0.3905	1.51	0.47	-32.8	2952.5	45.6	2575.8	30.8	2125.0	27.4	356	41	172	0.12	1.45
16SA-3-4@12	1.5944	1.50	0.2834	0.19	24.43	1.51	0.6266	1.50	0.99	-9.1	3378.6	3.0	3285.9	14.9	3136.1	37.4	439	219	389	0.50	0.10
16SA-7@01	1.5632	1.74	0.2714	0.42	22.12	1.84	0.6239	1.74	0.95	-4.0	3229.2	9.4	3189.0	18.0	3125.5	43.3	292	36	234	0.12	2.47
16SA-7@2	1.6210	1.57	0.2553	0.34	21.69	1.61	0.6167	1.57	0.98	-4.7	3216.6	5.3	3169.9	15.7	3096.6	38.8	410	45	325	0.11	0.04
16SA-7@3	1.7418	2.33	0.2574	0.88	19.48	2.49	0.5664	2.31	0.93	-11.2	3181.4	14.7	3066.1	24.3	2893.1	54.0	396	62	289	0.16	1.34

16SA-7@4	1.6901	1.50	0.2622	0.30	20.83	1.54	0.5868	1.50	0.97	-9.8	3231.3	5.5	3130.8	15.0	2976.4	35.9	484	70	363	0.15	0.82
16SA-7@5	1.5945	1.65	0.2573	0.21	22.18	1.67	0.6265	1.65	0.99	-3.6	3227.0	3.3	3191.7	16.3	3135.8	41.2	334	36	269	0.11	0.10
16SA-7@6	2.3075	1.53	0.2493	0.66	13.16	1.73	0.4183	1.53	0.88	-30.6	3039.4	12.9	2691.3	16.5	2252.8	29.2	503	31	264	0.06	3.47
16SA-7@7	1.5740	1.55	0.2579	0.20	22.56	1.56	0.6350	1.55	0.99	-2.5	3232.4	3.1	3208.1	15.3	3169.5	39.0	324	41	266	0.13	0.04
16SA-7@8	1.5690	1.58	0.2590	0.19	22.62	1.59	0.6361	1.58	0.99	-2.3	3233.7	3.1	3210.6	15.6	3173.8	39.8	267	32	219	0.12	0.19
16SA-7@9	1.5377	1.50	0.2594	0.25	23.18	1.52	0.6496	1.50	0.99	-0.5	3239.2	3.9	3234.4	14.9	3226.7	38.3	245	27	205	0.11	0.11
16SA-7@10	1.6701	1.53	0.2629	0.22	21.11	1.55	0.5936	1.53	0.99	-8.9	3233.9	3.8	3143.5	15.1	3004.0	36.8	275	32	210	0.12	0.86
16SA-7@11	1.5171	1.66	0.2751	0.83	22.97	2.12	0.6415	1.68	0.79	-1.9	3244.9	20.3	3225.7	20.8	3195.0	42.4	306	28	254	0.09	2.68
16SA-7@12	1.9830	1.51	0.2501	0.19	16.12	1.53	0.4932	1.51	0.99	-20.1	3100.0	3.9	2883.8	14.7	2584.6	32.2	475	4	[299]	0.01	2.19
16SA-8-2@01	1.5538	1.51	0.2597	0.13	22.76	1.51	0.6411	1.51	0.99	-1.5	3231.4	2.5	3216.8	14.8	3193.5	38.0	329	37	272	0.11	0.38
16SA-8-2@02	1.5350	1.55	0.2573	0.35	23.11	1.59	0.6515	1.55	0.97	0.2	3230.0	5.6	3231.5	15.6	3233.9	39.5	250	29	210	0.12	{0.08}
16SA-8-2@03	1.7368	2.15	0.2785	1.88	21.50	2.88	0.5705	2.13	0.74	-15.5	3325.1	30.0	3161.3	28.3	2909.7	50.1	151	42	116	0.28	0.92
16SA-8-2@04	1.5378	1.60	0.2979	0.64	26.62	1.73	0.6495	1.60	0.93	-8.4	3456.0	10.0	3369.7	17.1	3226.3	40.8	225	84	204	0.37	0.12
16SA-8-2@05	1.9524	1.54	0.2578	0.34	17.95	1.59	0.5099	1.54	0.97	-21.2	3217.8	6.0	2986.9	15.4	2656.3	33.6	312	47	207	0.15	0.44
16SA-8-2@06	1.6444	1.50	0.2553	0.31	21.12	1.54	0.6056	1.50	0.98	-5.9	3203.0	5.1	3143.9	15.0	3052.3	36.7	404	61	317	0.15	0.42
16SA-8-2@07	1.6126	1.51	0.2578	0.28	21.85	1.53	0.6185	1.51	0.98	-4.7	3223.9	4.5	3177.2	15.0	3103.8	37.3	355	47	283	0.13	0.26
16SA-8-2@08	1.5561	1.50	0.2584	0.25	22.83	1.53	0.6420	1.50	0.99	-1.4	3233.9	4.0	3219.7	14.9	3197.0	38.0	326	40	270	0.12	0.09
16SA-8-2@09	1.7049	1.55	0.2612	0.27	20.82	1.58	0.5839	1.55	0.98	-10.5	3238.2	4.6	3130.2	15.4	2964.6	36.9	266	38	202	0.14	0.45
16SA-8-2@10	2.1489	1.53	0.2330	1.35	14.41	2.08	0.4607	1.53	0.74	-23.2	3030.5	22.3	2777.3	19.9	2442.6	31.1	315	7	179	0.02	1.00
16SA-8-2@11	1.9379	1.57	0.2612	0.23	18.11	1.59	0.5119	1.57	0.99	-21.2	3225.7	3.8	2995.5	15.4	2664.6	34.4	463	89	310	0.19	0.80
16SA-8-2@12	1.7082	1.51	0.2584	0.26	20.66	1.54	0.5837	1.51	0.98	-10.1	3226.6	4.5	3122.8	15.0	2963.8	36.0	210	14	156	0.07	0.29
16SA-8-2@14	1.5603	1.50	0.2896	0.14	24.82	1.51	0.6342	1.50	0.99	-8.1	3384.1	2.4	3301.1	14.8	3166.1	37.7	322	283	288	0.88	1.05
16SA-9-1@01	1.6969	1.54	0.2539	0.47	20.53	1.62	0.5884	1.54	0.96	-8.6	3204.0	7.5	3116.7	15.8	2983.0	37.0	377	42	283	0.11	0.15
16SA-9-1@02	1.7820	3.34	0.2553	0.69	19.18	3.43	0.5561	3.31	0.97	-13.0	3185.2	13.9	3050.7	33.6	2850.7	76.8	46	24	35	0.53	0.89
16SA-9-1@03	1.6655	1.50	0.2599	0.70	21.32	1.67	0.5987	1.50	0.90	-8.2	3236.2	11.4	3153.2	16.3	3024.5	36.3	388	93	305	0.24	0.29
16SA-9-1@04	1.6967	1.59	0.2493	0.22	20.20	1.60	0.5888	1.59	0.99	-7.5	3176.8	3.5	3100.8	15.6	2984.8	38.0	730	119	555	0.16	0.09
16SA-9-1@05	1.9672	1.78	0.2420	0.28	16.67	1.80	0.5058	1.78	0.98	-18.5	3113.4	5.0	2916.0	17.4	2638.8	38.6	299	25	191	0.08	0.49
16SA-9-1@06	1.8323	1.59	0.2655	2.02	19.61	2.60	0.5425	1.59	0.61	-17.6	3259.6	32.0	3072.3	25.4	2794.0	36.2	354	165	267	0.47	0.59
16SA-9-1@07	1.7600	1.52	0.2623	0.29	19.94	1.55	0.5629	1.52	0.98	-13.4	3228.2	5.0	3088.7	15.1	2878.7	35.4	326	110	247	0.34	0.92
16SA-9-1@08	1.5523	1.50	0.2774	0.33	24.59	1.54	0.6438	1.50	0.98	-5.4	3346.4	5.2	3292.2	15.1	3204.0	38.0	200	42	172	0.21	0.06
16SA-9-1@09	1.7011	1.70	0.2635	0.72	20.94	1.92	0.5843	1.71	0.89	-10.8	3246.5	13.9	3135.9	18.8	2966.1	40.7	325	122	246	0.38	0.61
16SA-9-1@10	1.5732	1.56	0.2558	0.15	22.38	1.56	0.6353	1.56	1.00	-1.9	3219.4	2.4	3200.5	15.3	3170.6	39.1	292	21	236	0.07	0.05
16SA-9-1@11	1.5752	1.52	0.2546	0.26	22.24	1.54	0.6344	1.52	0.99	-1.7	3211.3	4.1	3194.2	15.1	3167.2	38.2	315	21	254	0.07	0.06
16SA-9-1@12	1.6331	1.56	0.2550	0.25	21.47	1.58	0.6119	1.56	0.99	-5.3	3213.2	3.9	3160.2	15.4	3077.4	38.3	314	22	244	0.07	0.08
**Mount T689**																					
16SA-33-1@01	1.5886	2.31	0.2672	0.74	23.11	2.42	0.6288	2.31	0.95	-5.4	3286.0	11.7	3231.6	23.9	3144.7	57.7	137	46	116	0.34	0.11
16SA-33-1@03	1.6741	1.81	0.2566	0.91	21.01	2.02	0.5963	1.81	0.89	-8.0	3219.7	14.3	3139.2	19.8	3014.9	43.6	262	78	208	0.30	0.17
16SA-33-1@4	1.5379	1.54	0.2487	0.18	22.30	1.55	0.6502	1.54	0.99	2.1	3176.8	2.8	3197.0	15.2	3229.2	39.3	363	120	313	0.33	{0.04}
16SA-33-1@5	1.5940	1.53	0.2479	0.51	21.41	1.61	0.6270	1.53	0.95	-1.3	3169.8	8.1	3157.4	15.7	3137.9	38.0	581	113	472	0.19	0.05
16SA-33-1@6	2.0193	2.01	0.2514	0.27	16.86	2.03	0.4926	2.00	0.99	-22.6	3173.9	5.3	2927.1	19.6	2581.8	42.7	136	75	92	0.55	0.53
16SA-33-1@7	2.6444	4.47	0.2208	1.01	11.04	4.54	0.3740	4.41	0.97	-35.2	2936.2	17.2	2526.2	43.2	2048.3	77.9	918	221	434	0.24	1.09
16SA-33-1@09	3.4772	3.12	0.1866	2.74	6.72	4.33	0.2817	3.06	0.71	-42.9	2586.7	50.3	2075.0	39.0	1599.9	43.5	1384	513	490	0.37	2.05
16SA-33-1@10	1.4493	1.61	0.2579	0.54	24.54	1.70	0.6900	1.61	0.95	5.9	3233.9	8.4	3289.9	16.7	3382.6	42.6	149	69	141	0.46	{0.03}
16SA-33-1@11	1.6707	1.68	0.2630	0.48	21.47	1.75	0.5965	1.68	0.96	-9.1	3253.2	7.8	3160.1	17.1	3015.7	40.6	153	56	122	0.37	0.34
16SA-33-1@13	1.4894	1.50	0.2602	0.49	24.09	1.58	0.6714	1.50	0.95	2.5	3247.9	7.6	3271.9	15.5	3311.3	39.1	181	62	164	0.34	{0.04}
16SA-33-1@16	1.5440	1.50	0.2604	0.38	23.18	1.55	0.6471	1.50	0.97	-1.1	3245.8	6.1	3234.7	15.2	3216.8	38.1	214	68	185	0.32	0.09
16SA-33-1@17	1.5541	1.52	0.2627	0.38	23.27	1.56	0.6432	1.52	0.97	-2.3	3261.4	5.9	3238.4	15.3	3201.4	38.4	238	92	208	0.38	0.05
16SA-33-1@18	6.2424	16.48	0.2163	3.28	4.43	16.57	0.1572	16.1	0.97	-71.9	2860.7	58.9	1717.3	147.4	940.9	143.0	1530	166	303	0.11	1.90
16SA-33-1@19	1.7025	1.98	0.2624	0.67	21.15	2.09	0.5866	1.98	0.95	-10.7	3256.2	10.5	3145.7	20.4	2975.5	47.2	297	115	237	0.39	0.14

16SA-33-1@20	1.5977	1.78	0.2649	0.77	22.61	1.95	0.6237	1.78	0.91	-5.4	3263.9	12.5	3210.1	19.2	3124.6	44.3	136	54	115	0.40	0.35
16SA-33-1@21	1.5118	2.10	0.2620	0.52	23.90	2.16	0.6615	2.10	0.97	0.5	3259.0	8.1	3264.3	21.3	3272.9	54.1	87	26	77	0.30	{0.27}
16SA-34@01	1.8712	1.52	0.2581	0.46	18.50	1.60	0.5299	1.53	0.95	-17.7	3204.9	7.5	3016.1	15.5	2741.1	34.2	202	84	143	0.42	0.84
16SA-34@02	1.7295	1.53	0.2590	0.51	20.30	1.62	0.5752	1.53	0.94	-11.3	3221.8	8.6	3105.6	15.8	2929.1	36.1	362	91	275	0.25	0.52
16SA-34@03	1.6065	1.50	0.2614	0.22	22.37	1.52	0.6219	1.50	0.99	-5.2	3251.8	3.5	3199.7	14.9	3117.4	37.2	405	121	336	0.30	0.09
16SA-34@04	1.6029	1.56	0.2642	1.02	22.59	1.87	0.6227	1.56	0.83	-5.6	3265.5	16.2	3209.4	18.4	3120.5	38.7	105	26	87	0.25	0.19
16SA-34@05	1.6199	1.53	0.2587	0.42	21.84	1.59	0.6158	1.53	0.96	-5.3	3229.6	6.8	3176.5	15.6	3093.1	37.8	220	88	182	0.40	0.25
16SA-34@06	1.8368	4.59	0.2576	0.47	19.16	4.61	0.5429	4.58	0.99	-16.3	3222.1	7.6	3050.0	45.4	2795.7	104.7	394	108	284	0.28	0.27
16SA-34@07	1.5497	1.52	0.2568	0.22	22.78	1.53	0.6446	1.52	0.99	-0.7	3223.8	3.6	3217.4	15.0	3207.1	38.4	187	40	158	0.22	0.10
16SA-34@08	5.6841	3.22	0.1351	4.01	2.91	5.55	0.1727	3.15	0.57	-52.2	1987.7	79.1	1384.3	42.8	1027.2	30.0	2153	65	426	0.03	1.81
16SA-34@09	2.7855	1.69	0.2478	1.01	10.02	2.12	0.3395	1.62	0.76	-41.2	2936.7	22.0	2436.8	19.8	1884.5	26.5	577	836	256	1.45	5.42
16SA-34@10	2.2195	1.55	0.2385	0.22	14.45	1.57	0.4474	1.55	0.99	-27.0	3081.5	3.8	2779.8	15.0	2383.5	30.9	656	794	404	1.21	0.71
16SA-34@11	1.6253	1.53	0.2703	0.79	22.86	1.72	0.6146	1.52	0.89	-8.2	3304.8	12.4	3221.1	16.9	3088.6	37.5	110	73	97	0.67	0.10
16SA-34@12	2.7758	3.24	0.1876	2.59	8.74	4.23	0.3553	3.18	0.75	-29.8	2639.2	45.6	2311.8	39.3	1959.7	54.0	1144	302	506	0.26	1.38
16SA-34@13	2.1274	1.54	0.2525	0.55	15.05	1.65	0.4587	1.54	0.93	-25.9	3106.8	9.7	2818.6	15.9	2433.8	31.2	466	207	273	0.44	2.41
16SA-34@14	4.9689	1.51	0.1984	5.40	4.11	8.72	0.1892	1.51	0.17	-58.7	2431.5	138.7	1657.2	73.8	1117.0	15.5	2169	453	491	0.21	5.99
16SA-34@15	1.6371	1.57	0.2584	0.23	21.54	1.60	0.6089	1.57	0.98	-6.2	3225.7	4.8	3163.2	15.6	3065.6	38.4	455	148	367	0.33	0.32
16SA-34@16	1.8057	1.52	0.2469	0.37	18.71	1.57	0.5526	1.52	0.97	-12.5	3156.2	5.9	3026.8	15.2	2835.8	35.1	445	128	325	0.29	0.23
16SA-34@17	2.0564	1.53	0.2330	0.54	15.36	1.63	0.4840	1.53	0.94	-20.1	3053.1	9.1	2837.8	15.7	2544.8	32.2	665	203	418	0.30	0.46
16SA-34@18	1.5870	1.52	0.2589	0.36	22.36	1.56	0.6290	1.52	0.97	-3.4	3233.5	5.7	3199.6	15.3	3145.7	37.9	152	52	126	0.34	0.18
16SA-36-1@01	1.4516	1.89	0.3039	1.18	28.23	2.23	0.6834	1.88	0.84	-4.1	3468.2	18.5	3427.2	22.1	3357.4	49.3	385	117	362	0.30	0.80
16SA-36-1@02	1.4330	2.08	0.2932	0.79	28.11	2.23	0.6970	2.09	0.94	-0.8	3431.1	12.2	3423.0	22.1	3409.2	55.5	218	79	210	0.36	0.12
16SA-36-1@03	1.9177	1.56	0.2832	1.36	20.01	2.08	0.5184	1.56	0.75	-24.3	3362.5	21.5	3091.7	20.4	2692.3	34.3	720	484	517	0.67	0.59
16SA-36-1@04	6.5283	11.66	0.2899	0.55	5.61	11.31	0.1487	11.3	1.00	-78.0	3327.0	11.1	1917.9	102.5	893.9	95.0	1712	468	359	0.27	2.90
16SA-36-1@05	2.4763	1.53	0.2853	0.38	15.24	1.58	0.3982	1.52	0.96	-41.5	3349.0	6.6	2830.1	15.2	2160.6	28.0	603	180	326	0.30	1.40
16SA-36-1@06	1.4808	1.67	0.3004	0.54	27.93	1.75	0.6750	1.67	0.95	-5.4	3470.8	8.3	3416.7	17.3	3325.1	43.5	352	91	325	0.26	0.05
16SA-36-1@07	2.5132	4.60	0.2620	4.41	13.78	6.49	0.3927	4.52	0.70	-39.2	3212.5	71.8	2734.4	63.4	2135.3	82.6	700	280	370	0.40	1.31
16SA-36-1@08	1.9497	2.09	0.2952	0.39	20.46	2.12	0.5093	2.07	0.98	-27.4	3424.9	6.9	3113.3	20.7	2653.5	45.3	327	99	229	0.30	0.71
16SA-36-1@09	1.5072	1.54	0.2886	0.22	26.10	1.56	0.6609	1.54	0.99	-4.8	3398.3	3.5	3350.3	15.3	3270.7	39.6	291	104	263	0.36	0.39
16SA-36-1@10	3.4539	6.90	0.2932	0.23	11.26	6.80	0.2857	6.79	1.00	-58.8	3395.5	4.7	2545.0	65.5	1619.8	98.0	823	583	324	0.71	1.33
16SA-36-1@11	1.6059	1.52	0.2993	0.38	25.54	1.57	0.6213	1.52	0.97	-12.5	3460.2	6.0	3328.9	15.5	3115.2	37.8	271	81	231	0.30	0.22
16SA-36-1@12	1.9039	2.46	0.2781	1.19	19.84	2.73	0.5226	2.45	0.90	-22.9	3336.4	18.9	3083.4	26.8	2710.3	54.4	472	288	345	0.61	0.50
16SA-36-1@13	2.3073	2.71	0.2547	1.18	14.95	2.96	0.4310	2.70	0.91	-32.8	3194.2	19.1	2811.9	28.5	2310.4	52.6	853	384	496	0.45	0.54
16SA-36-1@14	1.5206	1.66	0.2949	0.43	26.68	1.71	0.6571	1.66	0.97	-6.9	3441.5	6.6	3371.9	16.9	3256.1	42.5	420	309	398	0.74	0.07
16SA-36-1@15	2.0269	1.64	0.3042	0.50	19.13	1.73	0.4798	1.62	0.94	-31.3	3413.0	9.3	3048.3	16.8	2526.3	34.0	483	155	324	0.32	2.75
16SA-36-1@16	2.1880	4.46	0.2736	0.72	16.64	4.48	0.4518	4.41	0.98	-32.1	3289.3	12.2	2914.4	43.8	2403.2	89.0	930	382	575	0.41	1.15
16SA-36-2@01	1.5406	1.60	0.2582	0.51	23.08	1.68	0.6489	1.60	0.95	-0.4	3234.3	8.1	3230.2	16.5	3223.8	40.7	72	26	63	0.36	0.04
16SA-36-2@2	1.6225	1.94	0.2622	0.45	22.00	1.99	0.6138	1.94	0.97	-6.2	3246.0	7.3	3183.5	19.5	3085.3	47.6	104	57	90	0.55	0.41
16SA-36-2@3	1.5109	1.65	0.2581	0.45	23.56	1.71	0.6619	1.65	0.96	1.5	3235.4	7.1	3250.3	16.8	3274.4	42.5	78	30	71	0.39	{0.29}
16SA-36-2@05	1.5060	1.50	0.2563	0.27	23.46	1.52	0.6640	1.50	0.98	2.3	3223.9	4.3	3246.3	15.0	3282.7	38.7	336	172	310	0.51	{0.10}
16SA-36-2@6	1.5208	1.58	0.2562	0.29	23.23	1.60	0.6576	1.58	0.98	1.3	3223.7	4.6	3236.7	15.7	3257.7	40.5	323	177	297	0.55	{0.02}
16SA-36-2@7	1.5392	1.50	0.2572	0.86	22.78	1.74	0.6474	1.50	0.86	0.0	3217.2	13.8	3217.5	17.0	3218.1	38.1	60	23	53	0.38	0.35
16SA-36-2@8	8.5291	1.91	0.1356	0.50	2.15	1.98	0.1169	1.91	0.97	-70.4	2142.6	8.9	1164.6	13.8	712.5	12.9	1646	107	221	0.07	0.33
16SA-36-2@9	1.5287	1.52	0.2603	0.45	23.48	1.59	0.6542	1.52	0.96	-0.2	3248.5	7.1	3247.0	15.6	3244.5	38.9	132	52	118	0.39	{0.91}
16SA-36-2@11	1.5636	1.51	0.2599	0.95	22.73	1.79	0.6380	1.51	0.84	-2.2	3237.2	15.1	3215.6	17.6	3181.0	38.0	120	66	108	0.54	0.25
16SA-36-2@12	1.5643	1.50	0.2623	0.78	22.53	1.74	0.6342	1.50	0.87	-2.6	3232.3	13.6	3206.7	17.0	3166.0	37.8	75	30	65	0.39	0.80
16SA-36-2@13	1.5856	1.67	0.2600	0.85	22.56	1.87	0.6302	1.66	0.89	-3.6	3244.2	13.3	3208.0	18.3	3150.5	41.6	231	102	200	0.44	0.07

Note: The susscript "uc" means uncorrected, while the subscript "c" means corrected.

Supplementary Table S3 SIMS zircon U-Pb isotope results for the BGGT TTGs in South Africa (Part II-by SHRIMP)

Spot	% $^{206}Pb_c$	ppm U	ppm Th	$^{232}Th/^{238}U$	ppm $^{206}Pb^*$	(1) $^{206}Pb/^{238}U$ Age	(1) $^{207}Pb/^{206}Pb$ Age	% Dis-cor-dant	Total $^{238}U/^{206}Pb$	±%	Total $^{207}Pb/^{206}Pb$	±%	(1) $^{207}Pb^*/^{206}Pb^*$	±%	(1) $^{207}Pb^*/^{235}U$	±%	(1) $^{206}Pb^*/^{238}U$	±%	err corr
**Mount T1037**																			
17SA-14-2-1	4.77	189	42	0.23	85.8	2,578 ±160	3,106 ± 240	17	1.890	6.2	0.2800	4.7	0.2380	15	16.1	17	0.4920	8.2	.473
17SA-14-2-2	0.41	124	11	0.09	70	3,240 ± 34	3,225.00 ± 8.4	0	1.522	1.3	0.2601	0.45	0.2565	0.53	23.09	1.4	0.6530	1.3	.928
17SA-14-2-3	0.11	225	44	0.20	129	3,300 ± 33	3,235.80 ± 5.8	-2	1.493	1.3	0.2592	0.35	0.2582	0.37	23.8	1.3	0.6685	1.3	.961
17SA-14-2-4	0.33	74	22	0.31	43.4	3,325 ± 90	3,230 ± 11	-3	1.474	3.5	0.2602	0.58	0.2573	0.68	23.95	3.5	0.6750	3.5	.981
17SA-14-2-5	--	148	2	0.02	82.1	3,214 ± 33	3,237.00 ± 6.8	1	1.547	1.3	0.2584	0.43	0.2584	0.43	23.03	1.4	0.6464	1.3	.949
17SA-14-2-6	0.62	47	16	0.34	24.4	2,997 ± 55	3,214 ± 16	7	1.674	2.3	0.2602	0.71	0.2548	10	20.78	2.5	0.5920	2.3	.916
17SA-14-2-7	0.41	156	3	0.02	66	2,570 ± 36	3,147.20 ± 8.9	18	2.030	1.7	0.2478	0.47	0.2442	0.56	16.49	1.8	0.4897	1.7	.951
17SA-14-2-8	0.10	152	67	0.45	82.8	3,158 ± 32	3,236.00 ± 6.6	2	1.579	1.3	0.2591	0.41	0.2582	0.42	22.51	1.3	0.6322	1.3	.950
17SA-14-2-9	0.51	119	12	0.10	66.5	3,207 ± 33	3,221.20 ± 8.6	0	1.540	1.3	0.2603	0.46	0.2559	0.55	22.74	1.4	0.6446	1.3	.922
17SA-14-2-10	0.12	160	14	0.09	88	3,180 ± 32	3,237.50 ± 6.9	2	1.565	1.3	0.2595	0.41	0.2585	0.44	22.73	1.3	0.6377	1.3	.946
17SA-14-1-1	0.43	195	28	0.15	108	3,206 ±31	3,236.20 ±7.0	1	1.542	1.2	0.2621	0.38	0.2583	0.44	22.95	1.3	0.6444	1.2	.942
17SA-14-1-3	0.33	201	24	0.12	104	3,035 ±30	3,239.7 ± 6.7	6	1.655	1.2	0.2618	0.37	0.2589	0.43	21.45	1.3	0.6012	1.2	.945
17SA-14-1-4	0.46	117	2	0.01	63.9	3,147 ± 33	3,228.3 ± 8.9	3	1.578	1.3	0.2610	0.47	0.2570	0.57	22.3	1.4	0.6292	1.3	.920
17SA-14-1-5	0.03	207	1	0.01	115	3,223 ± 33	3,237.9 ± 6.1	0	1.541	1.3	0.2588	0.39	0.2586	0.39	23.13	1.4	0.6488	1.3	.958
17SA-14-1-7	0.35	141	23	0.17	67.1	2,829 ± 29	3,226.7 ± 9.0	12	1.806	1.3	0.2598	0.45	0.2568	0.57	19.5	1.4	0.5509	1.3	.914
17SA-14-1-10	0.27	554	223	0.42	128	1,532 ± 15	3,226.2 ± 6.7	52	3.711	1.1	0.2591	0.35	0.2567	0.42	9.5	1.2	0.2684	1.1	.936
17SA-10-1	--	137	47	0.35	85.5	3,515 ±34	3,459.0 ± 6.0	-2	1.379	1.3	0.2978	0.39	0.2978	0.39	29.77	1.3	0.7250	1.3	.956
17SA-10-2	0.07	138	47	0.35	86.7	3,526 ±34	3,456.2 ± 6.1	-2	1.372	1.3	0.2979	0.38	0.2973	0.39	29.84	1.3	0.7281	1.3	.955
17SA-10-3	0.22	192	75	0.40	117	3,446 ±40	3,445.7 ± 5.6	0	1.410	1.5	0.2972	0.32	0.2953	0.36	28.77	1.5	0.7070	1.5	.971
17SA-10-4	1.42	211	76	0.37	114	3,089 ±29	3,442.8 ± 7.6	10	1.592	1.2	0.3070	0.32	0.2949	0.52	24.98	1.3	0.6146	1.2	.925
17SA-10-5	0.04	202	107	0.55	119	3,364 ±33	3,459.2 ± 6.1	3	1.459	1.3	0.2982	0.39	0.2979	0.39	28.14	1.3	0.6851	1.3	.954
17SA-10-6	2.98	124	39	0.32	77.3	3,384 ±35	3,431 ± 16	1	1.382	1.3	0.3183	0.38	0.2928	1.1	27.83	1.7	0.6903	1.3	.798
17SA-10-7	0.02	222	88	0.41	135	3,444 ±31	3,448.3 ± 4.8	0	1.416	1.2	0.2959	0.31	0.2958	0.31	28.81	1.2	0.7063	1.2	.966
17SA-12-1	8.41	176	51	0.30	101	2,986 ± 38	2,731 ± 68	-9	1.506	1.3	0.2642	0.43	0.1892	4.3	15.33	4.5	0.5891	1.7	.378
17SA-12-2	19.72	203	655	3.33	67.5	1,685 ± 58	1,696 ± 560	1	2.587	1.3	0.2755	0.95	0.1040	29	4.3	31	0.2990	4.4	.142
17SA-12-3	1.56	193	7	0.04	107	3,156 ± 31	3,196 ± 13	1	1.547	1.2	0.2656	0.37	0.2519	0.85	21.92	1.5	0.6316	1.3	.839
17SA-12-4	8.09	177	177	1.04	91	2,757 ± 37	2,769 ± 75	0	1.670	1.2	0.2657	0.38	0.1936	4.6	14.21	4.9	0.5336	1.8	.367
17SA-12-5	5.40	138	22	0.16	76.1	3,003 ± 52	3,056 ± 33	2	1.556	2	0.2786	0.86	0.2309	2.2	18.86	3	0.5930	2.1	.717
17SA-12-6	1.10	168	37	0.23	85.3	2,955 ± 29	3,193 ± 9.8	7	1.692	1.2	0.2610	0.45	0.2514	0.64	20.15	1.4	0.5815	1.2	.894
17SA-12-7	23.01	191	296	1.60	99.6	2,401 ± 54	1,470 ± 400	-63	1.644	1.4	0.2899	1.1	0.0930	21	5.7	21	0.4510	2.8	.131
17SA-12-8	2.80	248	363	1.51	88.9	2,169 ± 23	3,127 ± 18	31	2.399	1.2	0.2659	0.43	0.2413	1.2	13.29	1.7	0.4000	1.2	.742
17SA-12-9	5.69	120	5	0.05	66.3	2,997 ±170	3,126 ± 110	4	1.550	6.9	0.2920	4	0.2410	6.8	19.7	9.9	0.5920	7.1	.714
17SA-12-10	0.54	544	1128	2.14	152	1,803 ± 84	3,236 ± 11	44	3.070	5.3	0.2629	0.6	0.2582	0.67	11.49	5.3	0.3230	5.3	.992
**Mount T1038**																			
17SA-8@7	3.62	2413	320	0.14	702	1,802 ±20	2,649 ± 33	32	2.952	1.2	0.2121	1.1	0.1797	2.1	7.98	2.3	0.3225	1.2	.533
17SA-8-6	0.21	241	23	0.10	135	3,217 ±33	3,221.7 ± 5.6	0	1.541	1.3	0.2577	0.32	0.2559	0.35	22.83	1.3	0.6471	1.3	.964
17SA-8-5	0.15	350	25	0.07	181	3,024 ±30	3,191.0 ± 4.6	5	1.667	1.2	0.2523	0.27	0.2510	0.29	20.72	1.3	0.5987	1.2	.973
17SA-8-4	3.85	502	42	0.09	235	2,677 ±29	3,110 ± 27	14	1.835	1.2	0.2728	0.65	0.2389	1.8	16.94	2.2	0.5149	1.4	.624

17SA-8-3	3.73	475	41	0.09	193	2,382 ±27	3,113 ± 17	23	2.118	1.3	0.2721	0.47	0.2392	1.2	14.72	1.7	0.4469	1.3	.773
17SA-8-2	0.27	390	25	0.07	199	2,988 ±30	3,134.5 ± 5.2	5	1.689	1.3	0.2446	0.29	0.2422	0.33	19.69	1.3	0.5897	1.3	.968
17SA-8-1	3.18	401	27	0.07	166	2,433 ±27	3,106 ± 26	22	2.082	1.3	0.2662	1	0.2381	1.7	15.03	2.1	0.4584	1.4	.631

Errors are 1-sigma; Pb_c and Pb^* indicate the common and radiogenic portions, respectively.

Error in Standard calibration was 0.34%(not included in above errors but required when comparing data from different mounts).

(1) Common Pb corrected using measured ^{204}Pb.

Supplementary Table S4 SIMS zircon in-situ oxygen isotope results for the BGGT TTGs in South Africa

Analysis	*Age (Ma)*	*IP(nA)*	$\delta^{18}O$	*2SE*
T689				
penglai@01		2.0738	5.17	0.31
penglai@04		2.0569	4.94	0.18
penglai@05		2.0932	5.32	0.19
penglai@06		2.0372	5.24	0.32
penglai@07		2.0487	5.55	0.21
penglai@08		2.0348	5.38	0.25
penglai@09		2.0350	5.38	0.26
penglai@10		2.0290	5.24	0.22
penglai@11		2.0410	5.28	0.17
penglai@12		2.0277	5.23	0.23
penglai@13		2.0348	5.22	0.22
penglai@14		2.0418	5.19	0.23
penglai@15		2.0575	5.33	0.13
penglai@16		2.0332	5.10	0.20
penglai@17		2.0335	5.13	0.23
penglai@18		2.0306	4.98	0.23
penglai@19		2.0261	5.34	0.19
penglai@2		2.0708	4.82	0.24
penglai@20		2.0145	5.47	0.26
penglai@21		1.6713	5.70	0.20
qinghu@01-1		2.0702	5.41	0.29
qinghu@02		2.0552	5.13	0.23
qinghu@03		2.0421	5.20	0.20
qinghu@04		2.0314	5.63	0.18
qinghu@05		2.0379	5.57	0.24
qinghu@06		2.0393	5.25	0.21
qinghu@07		2.0512	5.69	0.24
qinghu@08		2.0325	5.40	0.25
qinghu@09		2.0333	5.69	0.21
16SA-36-2@1	3232.2	2.0436	6.93	0.24
16SA-36-2@1	3232.2	2.0426	7.10	0.23
16SA-36-2@1	3232.2	2.0411	7.24	0.20
16SA-36-2@1	3232.2	2.0390	7.31	0.25
16SA-36-2@1	3232.2	2.0343	7.18	0.19
16SA-36-2@1	3232.2	2.0396	6.85	0.22
16SA-36-2@1	3232.2	2.0400	6.91	0.29
16SA-36-2@1	3232.2	2.0436	6.10	0.20
16SA-36-2@0	3232.2	2.0477	6.93	0.27
16SA-36-2@0	3232.2	2.0581	7.43	0.16
16SA-36-2@0	3232.2	2.0622	7.02	0.20
16SA-36-2@0	3232.2	2.0588	7.11	0.21
16SA-36-2@0	3232.2	2.0586	5.86	0.21
16SA-36-2@0	3232.2	2.0579	7.15	0.19
16SA-36-2@0	3232.2	2.0633	6.80	0.26
16SA-36-2@0	3232.2	2.0656	6.58	0.23
16SA-36-1@6	3464.1	2.0366	5.58	0.21
16SA-36-1@2	3464.1	2.0308	5.85	0.21
16SA-36-1@0	3464.1	2.0224	5.76	0.22
16SA-34@11	3251.8	1.8856	6.01	0.18
16SA-34@07	3251.8	2.0310	5.83	0.17
16SA-34@05	3251.8	2.0355	6.00	0.15
16SA-34@04	3251.8	2.0371	6.15	0.23
16SA-34@03	3251.8	2.0166	5.70	0.16
16SA-33-1@17	3252.7	2.0361	5.78	0.19
16SA-33-1@16	3252.7	2.0432	5.62	0.21
16SA-33-1@15	3252.7	2.0382	6.04	0.33
16SA-33-1@14	3252.7	2.0576	6.08	0.24
16SA-33-1@13	3252.7	2.0565	5.71	0.19
16SA-33-1@12	3252.7	2.0479	3.50	0.29
16SA-33-1@10	3252.7	2.0416	6.36	0.23
16SA-33-1@08	3252.7	2.0432	5.55	0.26
16SA-33-1@05	3252.7	2.0432	4.01	0.22
16SA-33-1@04	3252.7	2.0411	5.65	0.26
16SA-33-1@03	3252.7	2.0332	5.72	0.32
16SA-33-1@02	3252.7	2.0293	4.96	0.21
16SA-33-1@01	3252.7	2.0281	5.63	0.18
T687				
PENGLAI@09		1.8726	5.44	0.21
PENGLAI@10		1.8741	5.09	0.32
PENGLAI@11		1.8597	5.15	0.32
PENGLAI@12		1.8619	4.93	0.28
PENGLAI@13		1.8449	5.03	0.24
PENGLAI@14		1.8595	5.11	0.21
PENGLAI@15		1.8686	5.14	0.23
PENGLAI@16		1.8786	5.07	0.31
PENGLAI@17		1.8761	4.83	0.19
PENGLAI@18		1.8659	5.39	0.20
PENGLAI@19		1.8721	5.35	0.23
PENGLAI@20		1.8685	5.35	0.22
PENGLAI@21		1.8735	5.20	0.33
PENGLAI@22		1.8664	5.55	0.20
PENGLAI@23		1.8663	5.12	0.24
PENGLAI@24		1.8699	5.77	0.25
PENGLAI@01		1.8870	5.24	0.24
PENGLAI@2		1.8966	5.39	0.23
PENGLAI@3		1.8945	5.17	0.21
PENGLAI@04		1.8837	5.29	0.20
PENGLAI@05		1.8728	5.46	0.23
PENGLAI@06		1.8624	5.23	0.19
PENGLAI@07		1.8671	5.38	0.22
PENGLAI@08		1.8581	5.32	0.26
QINGHU@01		1.9292	5.48	0.23
QINGHU@02		1.8754	5.69	0.23
QINGHU@03		1.8630	5.34	0.24
QINGHU@04		1.8665	5.49	0.21
QINGHU@05		1.8584	5.66	0.18
QINGHU@06		1.8496	5.45	0.19
QINGHU@07		1.8727	5.70	0.23
QINGHU@08		1.8801	5.19	0.27
QINGHU@09		1.8760	5.50	0.25
QINGHU@10		1.8704	5.36	0.25
QINGHU@11		1.8671	5.23	0.16
QINGHU@12		1.8678	6.01	0.29
16SA-31-2@01	3248.3	1.8783	5.56	0.32
16SA-31-2@03	3248.3	1.8715	5.79	0.19

Analysis	Age (Ma)	IP(nA)	$\delta^{18}O$	2SE	Analysis	Age (Ma)	IP(nA)	$\delta^{18}O$	2SE
16SA-31-2@0	3248.3	1.8687	6.09	0.18	Penglai@06		1.7198	4.88	0.23
16SA-31-2@0	3248.3	1.8661	5.55	0.23	Penglai@07		1.7317	5.12	0.31
16SA-31-2@0	3248.3	1.8706	6.10	0.19	Penglai@08		1.7289	5.16	0.14
16SA-31-2@0	3248.3	1.8686	5.46	0.26	Penglai@09		1.7217	5.38	0.14
16SA-31-2@0	3248.3	1.8679	5.82	0.26	Penglai@10		1.7282	5.24	0.23
16SA-31-2@0	3248.3	1.8742	5.89	0.27	Penglai@11		1.7183	5.22	0.25
16SA-31-2@1	3248.3	1.8742	5.87	0.18	Penglai@12		1.7352	5.36	0.23
16SA-31-2@1	3248.3	1.8693	5.37	0.28	Penglai@13		1.7230	5.63	0.17
16SA-31-2@1	3248.3	1.8648	6.17	0.21	Penglai@14		1.7513	5.42	0.30
16SA-31-2@1	3248.3	1.8637	5.90	0.24	Penglai@15		1.7644	5.18	0.22
16SA-31-2@1	3248.3	1.8657	5.90	0.22	Penglai@16		1.8021	5.27	0.21
16SA-31-2@1	3248.3	1.8672	6.10	0.17	Penglai@17		1.7917	5.18	0.15
16SA-31-1@0	3242.9	1.8690	6.06	0.23	Penglai@18		1.7849	5.37	0.31
16SA-31-1@3	3242.9	1.8763	5.61	0.18	Penglai@19		1.7626	5.21	0.27
16SA-31-1@4	3242.9	1.8761	5.69	0.23	Penglai@20		1.7508	5.74	0.25
16SA-31-1@5	3242.9	1.8701	5.51	0.22	Penglai@21		1.7329	5.25	0.29
16SA-31-1@7	3242.9	1.8627	5.85	0.18	Penglai@22		1.7358	5.52	0.18
16SA-31-1@8	3242.9	1.8672	6.23	0.21	Penglai@23		1.7355	5.00	0.21
16SA-31-1@9	3242.9	1.8694	5.62	0.18	Penglai@24		1.7345	5.17	0.31
16SA-31-1@1	3242.9	1.8715	6.27	0.30	Penglai@25		1.7311	5.17	0.28
16SA-29@01	3244.3	1.8576	5.81	0.25	Penglai@26		1.7188	5.28	0.23
16SA-29@02	3244.3	1.8598	4.90	0.16	Penglai@27		1.7263	5.14	0.28
16SA-29@03	3244.3	1.8652	5.80	0.14					
16SA-29@04	3244.3	1.8686	5.55	0.22	Qinghu@01		1.7853	5.53	0.14
16SA-29@05	3244.3	1.8660	5.37	0.16	Qinghu@02		1.7252	5.67	0.30
16SA-29@06	3244.3	1.8691	4.87	0.20	Qinghu@03		1.7360	5.42	0.19
16SA-29@07	3244.3	1.8541	5.75	0.16	Qinghu@04		1.7194	5.56	0.27
16SA-29@08	3244.3	1.8608	5.41	0.29	Qinghu@05		1.7215	5.45	0.28
16SA-29@09	3244.3	1.8626	5.88	0.22	Qinghu@06		1.7239	5.53	0.28
16SA-29@10	3244.3	1.8626	5.78	0.30	Qinghu@07		1.7571	4.98	0.21
16SA-29@12	3244.3	1.8712	5.75	0.33	Qinghu@08		1.7842	5.06	0.34
16SA-29@13	3244.3	1.8736	6.01	0.22	Qinghu@09		1.7557	5.43	0.17
16SA-29@15	3244.3	1.9020	5.69	0.28	Qinghu@10		1.7266	5.18	0.21
16SA-29@16	3244.3	1.8646	5.52	0.18	Qinghu@11		1.7342	5.19	0.30
16SA-29@17	3244.3	1.8636	5.48	0.15	Qinghu@12		1.7328	5.17	0.18
16SA-29@18	3244.3	1.8618	5.77	0.38	Qinghu@13		1.7273	5.48	0.26
16SA-29@19	3244.3	1.8654	6.09	0.23	16SA-20-2@17	3251	1.7270	6.24	0.23
16SA-29@20	3244.3	1.8668	6.08	0.20	16SA-20-2@16	3251	1.7270	5.78	0.36
16SA-29@21	3244.3	1.8635	6.11	0.21	16SA-20-2@15	3251	1.7348	5.64	0.28
16SA-28-1@0	3250	1.8629	5.65	0.27	16SA-20-2@14	3251	1.7257	5.91	0.22
16SA-28-1@0	3250	1.8790	5.41	0.21	16SA-20-2@13	3251	1.7255	6.13	0.22
16SA-28-1@0	3250	1.8807	6.24	0.17	16SA-20-2@12	3251	1.7302	5.98	0.27
16SA-28-1@0	3250	1.8855	5.48	0.24	16SA-20-2@11	3251	1.7220	6.10	0.21
16SA-28-1@1	3250	1.8822	5.42	0.17	16SA-20-2@10	3251	1.7214	5.59	0.25
16SA-28-1@1	3250	1.8812	5.57	0.27	16SA-20-2@09	3251	1.7195	5.65	0.19
16SA-28-1@1	3250	1.9131	5.89	0.20	16SA-20-2@08	3512	1.7202	5.78	0.22
16SA-28-1@1	3250	1.8733	5.88	0.33	16SA-20-2@07	3251	1.7255	5.96	0.31
16SA-28-1@1	3250	1.8755	5.67	0.26	16SA-20-2@06	3251	1.7271	5.88	0.17
16SA-28-1@1	3250	1.8749	6.34	0.19	16SA-20-2@05	3251	1.7265	5.58	0.21
T688					16SA-20-2@04	3251	1.7313	5.77	0.24
Penglai@01		1.7869	5.29	0.29	16SA-20-2@03	3251	1.7281	5.72	0.23
Penglai@2		1.7841	5.35	0.20	16SA-20-2@02	3251	1.7230	5.91	0.34
Penglai@3		1.7840	5.02	0.23	16SA-20-2@01	3251	1.7187	5.87	0.28
Penglai@04		1.7504	4.83	0.26	16SA-18-3@01	3423.1	1.7121	5.00	0.20
Penglai@05		1.7327	5.39	0.52	16SA-18-3@02	3423.1	1.7128	5.00	0.30

Analysis	Age (Ma)	IP(nA)	$\delta^{18}O$	2SE
16SA-18-3@0	3423.1	1.7239	5.36	0.31
16SA-18-3@0	3423.1	1.7254	5.44	0.21
16SA-18-3@0	3423.1	1.7313	2.84	0.26
16SA-18-3@0	3423.1	1.7678	4.64	0.19
16SA-18-3@0	3423.1	1.7274	5.01	0.26
16SA-18-3@0	3423.1	1.7281	5.15	0.19
16SA-18-3@0	3423.1	1.7222	5.05	0.29
16SA-18-3@1	3423.1	1.7165	5.16	0.32
16SA-18-2@0	3205.6	1.7345	6.67	0.31
16SA-18-2@0	3205.6	1.7420	6.13	0.19
16SA-18-2@0	3205.6	1.7525	6.78	0.15
16SA-18-2@0	3404.8	1.7531	5.42	0.26
16SA-18-2@0	3205.6	1.7920	5.98	0.29
16SA-18-2@1	3205.6	1.7622	6.06	0.34
16SA-18-2@1	3205.6	1.7714	5.99	0.25
16SA-18-2@1	3205.6	1.8035	5.83	0.17
16SA-18-2@1	3205.6	1.7872	5.19	0.32
16SA-18-2@2	3205.6	1.7852	6.59	0.24
16SA-18-2@2	3205.6	1.7846	6.60	0.26
16SA-18-2@2	3205.6	1.7777	7.78	0.27
16SA-18-2@2	3205.6	1.7766	6.15	0.29
16SA-18-2@2	3205.6	1.7729	5.72	0.24
16SA-18-2@2	3205.6	1.7619	6.32	0.22
T690				
penglai2@1		2.0818	5.39	0.16
penglai2@2		2.0834	5.12	0.22
penglai2@3		2.0792	5.35	0.19
penglai@05		2.0582	5.04	0.18
penglai@06		2.0622	4.76	0.26
penglai@07		2.0503	5.42	0.16
penglai@08		2.0995	5.15	0.20
penglai@09		2.0377	5.16	0.26
penglai@10		2.0335	5.15	0.19
penglai@11		2.0267	5.00	0.21
penglai@12		2.0373	5.73	0.21
penglai@13		2.0250	5.25	0.19
penglai@15		2.0205	5.40	0.17
penglai@16		2.0235	5.48	0.28
penglai@17		2.0362	5.20	0.24
penglai@18		2.0271	5.43	0.32
penglai@19		2.0307	5.74	0.25
penglai@20		2.0207	5.14	0.34
penglai@21		2.0277	5.34	0.24
penglai@23		2.0309	5.01	0.19
penglai@24		2.0238	5.00	0.27
qinghu@02		2.0591	5.29	0.17
qinghu@03		2.0473	5.33	0.24
qinghu@04		2.0300	5.37	0.18
qinghu@05		2.0227	5.54	0.18
qinghu@06		2.0268	5.20	0.13
qinghu@08		2.0206	5.22	0.28
qinghu@09		2.0367	5.76	0.17
qinghu@10		2.0277	5.71	0.28
qinghu@11		2.0274	5.67	0.19

Analysis	Age (Ma)	IP(nA)	$\delta^{18}O$	2SE
qinghu@12		2.0300	4.89	0.25
qinghu2@1		2.0705	5.23	0.20
16SA-9-2@18	3230	2.0312	6.47	0.25
16SA-9-2@17	3230	2.0328	6.51	0.13
16SA-9-2@16	3230	2.0304	6.70	0.18
16SA-9-2@15	3230	2.0289	6.40	0.18
16SA-9-2@12	3230	2.0185	6.22	0.23
16SA-9-2@07	3230	2.0304	6.51	0.19
16SA-9-2@06	3230	2.0315	6.17	0.19
16SA-9-2@04	3230	2.0168	6.42	0.13
16SA-9-2@03	3230	2.0196	6.17	0.22
16SA-9-2@01	3230	2.0246	6.66	0.21
16SA-11@01	3458.6	2.0603	5.45	0.18
16SA-11@3	3458.6	2.0610	5.49	0.28
16SA-11@4	3458.6	2.0502	5.55	0.24
16SA-11@5	3458.6	2.0515	5.85	0.22
16SA-11@6	3458.6	2.0541	6.19	0.16
16SA-11@7	3458.6	2.0521	5.51	0.23
16SA-11@9	3458.6	2.0537	6.26	0.22
16SA-10-1@01	3460	2.0278	5.96	0.24
16SA-10-1@2	3460	2.0310	6.20	0.13
16SA-10-1@3	3460	2.0332	5.65	0.28
16SA-10-1@4	3460	2.0339	4.87	0.18
16SA-10-1@7	3460	2.0370	5.84	0.30
16SA-10-1@09	3460	2.0603	5.79	0.28
16SA-10-1@10	3460	2.0656	5.79	0.29
T692				
Penglai@01		1.9929	5.22	0.20
Penglai@3		1.9974	4.77	0.16
Penglai@04		1.9914	5.20	0.19
Penglai@05		1.9846	5.35	0.16
Penglai@06		1.9896	5.18	0.23
Penglai@07		1.9786	5.03	0.22
Penglai@08		1.9806	5.02	0.21
Penglai@09		1.9836	5.38	0.18
Penglai@10		1.9886	5.55	0.20
Penglai@11		1.9861	5.35	0.22
Penglai@12		1.9739	5.24	0.27
Penglai@13		1.9721	5.04	0.15
Penglai@14		1.9738	5.06	0.24
Penglai@15		1.9814	4.87	0.21
Penglai@16		1.9689	5.52	0.22
Penglai@17		1.9634	5.59	0.20
Penglai@18		1.9512	5.10	0.24
Penglai@19		1.9651	5.47	0.28
Penglai@20		1.9624	5.35	0.39
Penglai@21		1.9667	5.59	0.18
Penglai@22		1.9547	5.12	0.27
Penglai@23		1.9674	5.33	0.22
Penglai@24		1.9589	5.30	0.29
Penglai@25		1.9645	5.35	0.26
Penglai@26		1.9748	5.29	0.25
Qinghu@01		1.9824	5.45	0.26
Qinghu@02		1.9815	5.70	0.35

Analysis	Age (Ma)	IP(nA)	δ18O	2SE
Qinghu@03		1.9727	5.30	0.23
Qinghu@04		1.9805	5.41	0.19
Qinghu@05		1.9836	5.07	0.31
Qinghu@06		1.9753	5.34	0.31
Qinghu@07		1.9846	5.77	0.21
Qinghu@08		1.9777	5.96	0.23
Qinghu@09		1.9642	5.53	0.16
Qinghu@10		1.9648	5.89	0.25
Qinghu@12		1.9627	5.77	0.25
Qinghu@13		1.9679	5.53	0.19
16SA-9-1@10	3217.8	1.9617	6.54	0.15
16SA-8-2@20	3229.5	1.9559	6.69	0.16
16SA-8-2@19	3229.5	1.9599	6.56	0.28
16SA-8-2@18	3229.5	1.9584	6.70	0.20
16SA-8-2@17	3229.5	1.9738	6.49	0.16
16SA-8-2@16	3229.5	1.9710	7.06	0.20
16SA-8-2@15	3229.5	1.9724	6.92	0.17
16SA-8-2@14	3229.5	1.9712	6.77	0.25
16SA-8-2@13	3229.5	1.9724	6.27	0.21
16SA-8-2@08	3229.5	1.9771	6.38	0.24
16SA-8-2@07	3229.5	1.9767	7.27	0.25
16SA-8-2@02	3229.5	1.9724	6.20	0.20
16SA-8-2@01	3229.5	1.9720	6.53	0.21
16SA-7@11	3232.7	1.9777	6.69	0.23
16SA-7@09	3232.7	1.9768	6.96	0.19
16SA-7@08	3232.7	1.9710	6.51	0.25
16SA-7@07	3232.7	1.9678	6.64	0.15
16SA-7@05	3232.7	1.9703	6.66	0.33
16SA-7@02	3232.7	1.9823	6.37	0.34
16SA-7@01	3232.7	1.9740	6.23	0.23
16SA-3-4@16	3428.3	1.9815	5.31	0.35
16SA-3-4@15	3428.3	1.9778	5.58	0.14
16SA-3-4@14	3428.3	1.9745	5.38	0.17
16SA-3-4@13	3428.3	1.9747	4.53	0.25
16SA-3-4@09	3428.3	1.9771	5.44	0.37
16SA-3-4@07	3428.3	1.9713	5.40	0.23
16SA-3-4@02	3428.3	2.0240	5.11	0.23
16SA-1@12	3106.1	1.9800	6.92	0.20
16SA-1@10	3106.1	1.9786	7.04	0.13
16SA-1@09	3106.1	1.9827	6.78	0.22
16SA-1@07	3106.1	1.9878	6.91	0.18
16SA-1@06	3106.1	1.9879	6.86	0.23
16SA-1@04	3106.1	1.9839	5.97	0.21
16SA-1@03	3106.1	1.9768	6.79	0.24
16SA-1@01	3106.1	1.9799	6.24	0.15
T289				
PENGLAI@01		2.3893	5.52	0.21
PENGLAI@2		2.3916	5.48	0.13
PENGLAI@3		2.3845	5.33	0.21
PENGLAI@04		2.3132	4.78	0.18
PENGLAI@05		2.3158	5.25	0.21
PENGLAI@06		2.3026	4.94	0.17
PENGLAI@07		2.3035	5.24	0.22
PENGLAI@08		2.3067	4.95	0.19
PENGLAI@09		2.3078	5.25	0.16
PENGLAI@10		2.3031	5.18	0.22
PENGLAI@11		2.3024	5.58	0.18
PENGLAI@12		2.3054	5.21	0.19
PENGLAI@13		2.2922	5.20	0.19
PENGLAI@14		2.2891	5.53	0.21
PENGLAI@15		2.2775	5.32	0.18
QINGHU@01		2.3700	5.65	0.15
QINGHU@02		2.3183	5.58	0.16
QINGHU@03		2.3075	5.48	0.16
QINGHU@04		2.3079	5.79	0.17
QINGHU@05		2.3085	5.57	0.18
QINGHU@06		2.2930	5.50	0.22
QINGHU@07		2.2783	5.32	0.20
14SA-25@7	3225.5	2.2824	5.95	0.17
14SA-25@6	3225.5	2.2841	6.18	0.19
14SA-25@5	3225.5	2.2870	6.14	0.24
14SA-25@4	3225.5	2.2886	6.27	0.16
14SA-25@3	3225.5	2.2957	6.03	0.16
14SA-25@2	3225.5	2.2994	6.45	0.23
14SA-25@01	3225.5	2.2919	6.39	0.13
14SA-23@8	3222	2.2964	6.50	0.21
14SA-23@7	3222	2.3368	6.11	0.12
14SA-23@6	3222	2.3466	6.03	0.13
14SA-23@5	3222	2.2965	6.25	0.12
14SA-23@4	3222	2.2980	5.91	0.20
14SA-23@3	3222	2.2979	6.22	0.15
14SA-23@2	3222	2.3054	6.19	0.19
14SA-23@01	3222	2.3114	6.06	0.17
14SA-22@9	3453	2.3071	5.47	0.20
14SA-22@7	3453	2.3087	4.83	0.18
14SA-22@5	3453	2.3118	6.11	0.17
14SA-22@4	3453	2.3148	5.69	0.18
14SA-22@3	3453	2.3090	5.99	0.16
14SA-22@2	3453	2.3100	6.19	0.17
14SA-22@01	3453	2.3088	6.52	0.23
14SA-21@8	3454	2.3106	6.16	0.22
14SA-21@7	3454	2.3114	6.14	0.19
14SA-21@6	3454	2.3114	5.91	0.13
14SA-21@5	3454	2.3040	5.88	0.12
14SA-21@2	3454	2.3027	6.01	0.20
14SA-21@01	3454	2.3042	5.88	0.18
14SA-11@12	3448	2.3186	5.76	0.20
14SA-11@11	3448	2.3208	5.97	0.18
14SA-11@08	3448	2.3686	5.91	0.23
14SA-11@07	3448	2.3115	5.65	0.14
14SA-11@05	3448	2.3193	5.70	0.23
14SA-11@04	3448	2.3186	5.62	0.19
14SA-11@03	3448	2.3211	5.83	0.17
14SA-11@02	3448	2.3205	5.43	0.24
14SA-11@01	3448	2.3207	5.70	0.21
T290				
PENGLAI@01		2.3076	5.22	0.23
PENGLAI@2		2.3158	5.15	0.13
PENGLAI@3		2.3216	5.44	0.17
PENGLAI@04		2.3170	4.91	0.23

Analysis	Age (Ma)	IP(nA)	$\delta^{18}O$	2SE
PENGLAI@05		2.3477	5.15	0.24
PENGLAI@06		2.3684	5.04	0.21
PENGLAI@07		2.3351	5.41	0.24
PENGLAI@08		2.3346	5.05	0.19
PENGLAI@09		2.3258	5.11	0.14
PENGLAI@10		2.3376	5.25	0.15
PENGLAI@11		2.3347	4.95	0.16
PENGLAI@12		2.3132	4.96	0.17
PENGLAI@13		2.3172	5.71	0.18
PENGLAI@14		2.3186	5.16	0.25
PENGLAI@15		2.3279	5.35	0.16
PENGLAI@16		2.3297	5.25	0.13
PENGLAI@17		2.3152	5.59	0.16
PENGLAI@18		2.3337	5.61	0.19
PENGLAI@19		2.3164	5.17	0.18
PENGLAI@20		2.3240	5.51	0.17
QINGHU@01		2.3117	5.58	0.19
QINGHU@02		2.3420	5.33	0.22
QINGHU@03		2.3359	5.56	0.13
QINGHU@04		2.3253	5.60	0.19
QINGHU@05		2.3279	5.34	0.30
QINGHU@06		2.3159	5.56	0.23
QINGHU@07		2.3264	5.68	0.12
QINGHU@08		2.3135	5.55	0.28
QINGHU@09		2.3154	5.19	0.23
QINGHU@10		2.3268	5.27	0.23
14SA-5-1@07	3452	2.3321	5.31	0.17
14SA-21-3@1[illegible]	3447	2.3093	5.47	0.16
14SA-21-3@0[illegible]	3447	2.3156	5.56	0.19
14SA-21-3@0[illegible]	3447	2.3187	5.72	0.19
14SA-21-3@0[illegible]	3447	2.3233	5.31	0.22
14SA-21-3@0[illegible]	3447	2.3275	5.81	0.15
14SA-21-3@0[illegible]	3447	2.3417	5.44	0.28
14SA-21-3@0[illegible]	3447	2.3431	5.76	0.33
14SA-21-3@0	3447	2.3362	6.25	0.22
14SA-18-1@1	3266	2.3412	5.21	0.16
14SA-18-1@1[illegible]	3266	2.3386	3.55	0.21
14SA-18-1@0[illegible]	3266	2.3405	5.36	0.16
14SA-18-1@0[illegible]	3266	2.3369	5.78	0.12
14SA-18-1@0[illegible]	3266	2.3436	5.79	0.24
14SA-18-1@0[illegible]	3266	2.3541	4.79	0.19
14SA-18-1@0[illegible]	3266	2.3673	5.35	0.16
14SA-18-1@0[illegible]	3266	2.3752	4.53	0.18
14SA-16@15	3232	2.3293	5.81	0.12
14SA-16@14	3232	2.3235	5.87	0.14
14SA-16@13	3232	2.3208	5.86	0.20
14SA-16@12	3232	2.3140	6.24	0.13
14SA-16@11	3232	2.3124	5.76	0.24
14SA-16@10	3232	2.3174	5.83	0.15
14SA-16@09	3232	2.3840	5.81	0.16
14SA-16@08	3232	2.3247	5.79	0.21
14SA-16@07	3232	2.3269	4.68	0.25
14SA-16@05	3232	2.3225	6.10	0.16
14SA-16@03	3232	2.3116	5.63	0.29
14SA-16@01	3232	2.3129	5.85	0.15
T120				
QH@12		2.2898	5.67	0.15
PENGLAI1@01		2.2833	5.64	0.19
PENGLAI1@02		2.2823	5.04	0.18
QH@13		2.4263	5.18	0.24
PENGLAI1@03		2.2539	5.32	0.21
PENGLAI1@04		2.2334	5.13	0.16
14SA-20-1@03	3451.8	2.2956	5.41	0.20
14SA-20-1@05	3451.8	2.2970	5.77	0.15
14SA-20-1@06	3451.8	2.2905	5.34	0.20
14SA-20-1@07	3451.8	2.2950	4.87	0.16
14SA-20-1@11	3451.8	2.2709	5.63	0.19
14SA-20-1@12	3451.8	2.2705	5.35	0.16
14SA-20-1@13	3451.8	2.2535	5.21	0.25
14SA-20-1@14	3451.8	2.2949	5.82	0.16
14SA-20-1@15	3451.8	2.2977	5.31	0.17
14SA-20-1@16	3451.8	2.2313	5.33	0.24
14SA-20-1@19	3451.8	2.2548	5.18	0.18
QH@14		2.2576	5.28	0.15
PENGLAI1@05		2.2519	5.77	0.25
PENGLAI1@06		2.2569	5.16	0.12
QH@15		2.3022	5.25	0.14
PENGLAI1@07		2.2495	5.38	0.12
PENGLAI1@08		2.2731	4.96	0.13
QH@16		2.3133	5.08	0.19
PENGLAI1@09		2.2740	5.64	0.19
PENGLAI1@10		2.2500	5.03	0.19
QH@17		2.2728	5.19	0.13
PENGLAI1@11		2.2594	5.60	0.12
PENGLAI1@12		2.2306	5.30	0.17
QH@18		2.2229	5.06	0.12
PENGLAI1@13		2.2266	5.05	0.22
PENGLAI1@14		2.2186	5.54	0.16
T121				
QH@01		1.9767	5.99	0.19
PENGLAI1@01		1.9698	5.88	0.13
PENGLAI1@02		1.9419	5.64	0.20
QH@02		1.9347	6.28	0.16
PENGLAI1@03		1.9318	5.48	0.20
PENGLAI1@04		1.9146	5.33	0.25
QH@03		1.9116	5.54	0.25
PENGLAI1@05		1.9168	5.18	0.27
PENGLAI1@06		1.9263	5.34	0.16
QH@04		1.9746	5.69	0.22
PENGLAI1@07		1.9272	5.24	0.20
PENGLAI1@08		1.9258	5.21	0.13
14SA-20-2@04	3214.7	1.9009	6.21	0.18
14SA-20-2@06	3214.7	1.9293	7.04	0.23
14SA-20-2@10	3214.7	1.9271	6.51	0.16
14SA-20-2@13	3214.7	1.9544	5.71	0.20
14SA-20-2@21	3214.7	1.9413	6.55	0.17
QH@05		1.9369	5.70	0.21
PENGLAI1@09		1.9520	5.06	0.17
PENGLAI2@01		1.9485	5.41	0.19

Analysis	***Age (Ma)***	***IP(nA)***	**$\delta^{18}O$**	***2SE***	***Analysis***	***Age (Ma)***	***IP(nA)***	**$\delta^{18}O$**	***2SE***
QH@06		1.9383	5.49	0.14	QINGHU@07		2.0972	5.35	0.13
PENGLAI2@02		1.9442	5.47	0.18	QINGHU@08		2.0801	5.49	0.13
PENGLAI2@03		1.9572	5.43	0.15	QINGHU@09		2.0669	5.35	0.12
QH@07		1.9628	5.47	0.12	QINGHU@10		2.0349	5.43	0.16
PENGLAI2@04		1.9603	5.19	0.21	QINGHU@11		2.0291	5.35	0.13
PENGLAI2@05		1.9700	5.31	0.31	17SA-14-2@16	3233.4	2.0937	5.46	0.16
QH@08		1.9647	5.58	0.12	17SA-14-2@15	3233.4	2.0846	5.49	0.13
PENGLAI2@06		1.9613	5.38	0.18	17SA-14-2@14	3233.4	2.0975	5.58	0.17
PENGLAI2@07		1.9677	5.15	0.18	17SA-14-2@13	3233.4	2.0897	5.49	0.10
T1038					17SA-14-2@12	3233.4	2.0963	5.33	0.17
PENGLAI@01		2.0501	5.23	0.15	17SA-14-2@11	3233.4	2.0978	5.76	0.17
PENGLAI@04		2.0319	5.40	0.15	17SA-14-2@10	3233.4	2.0838	5.33	0.17
PENGLAI@05		2.0215	5.13	0.16	17SA-14-2@09	3233.4	2.1060	5.16	0.17
PENGLAI@06		2.0333	5.43	0.11	17SA-14-2@08	3233.4	2.0987	4.48	0.23
PENGLAI@07		2.0200	5.38	0.20	17SA-14-2@06	3233.4	2.0930	4.09	0.18
PENGLAI@08		2.0155	5.16	0.17	17SA-14-2@05	3233.4	2.0928	5.55	0.17
PENGLAI@09		2.0198	5.25	0.19	17SA-14-2@04	3233.4	2.0945	5.92	0.12
PENGLAI@10		2.0182	5.13	0.16	17SA-14-2@03	3233.4	2.1035	5.59	0.13
PENGLAI@11		2.0107	4.88	0.11	17SA-14-2@02	3233.4	2.0896	5.32	0.15
PENGLAI@2		2.0408	5.40	0.14	17SA-14-1@16	3233.5	2.1015	5.53	0.14
PENGLAI@3		2.0350	5.37	0.22	17SA-14-1@15	3233.5	2.0949	5.50	0.16
QINGHU@01		2.0300	5.61	0.17	17SA-14-1@14	3233.5	2.0939	5.61	0.12
QINGHU@02		2.0271	5.60	0.15	17SA-14-1@13	3233.5	2.1174	5.62	0.14
QINGHU@03		2.0282	5.84	0.14	17SA-14-1@12	3233.5	2.1285	5.59	0.13
QINGHU@04		2.0212	5.65	0.25	17SA-14-1@11	3233.5	2.1253	5.62	0.16
QINGHU@05		2.0194	5.81	0.22	17SA-14-1@05	3233.5	2.1209	5.64	0.12
17SA-8@06	3220	2.0265	7.37	0.18	17SA-14-1@04	3233.5	2.1166	5.82	0.22
17SA-8@05	3220	2.0257	6.54	0.15	17SA-14-1@01	3233.5	2.1212	5.42	0.12
17SA-8@02	3220	2.0199	6.48	0.23	17SA-12@18	*3235*	2.0374	4.84	0.20
T1037					17SA-12@17	*3235*	2.0405	5.02	0.21
PENGLAI@01		2.2690	5.72	0.22	17SA-12@16	*3235*	2.0591	3.94	0.18
PENGLAI@2		2.2789	5.29	0.12	17SA-12@15	*3235*	2.0507	5.23	0.11
PENGLAI@3		2.2814	5.52	0.21	17SA-12@14	*3235*	2.0395	4.51	0.20
PENGLAI@04		2.2351	5.23	0.13	17SA-12@13	*3235*	2.0440	5.44	0.11
PENGLAI@05		2.2292	5.29	0.12	17SA-12@12	*3235*	2.0506	5.00	0.18
PENGLAI@06		2.2093	5.21	0.12	17SA-12@11	*3235*	2.0462	5.43	0.15
PENGLAI@07		2.2114	5.08	0.15	17SA-12@09	*3235*	2.0234	5.05	0.17
PENGLAI@08		2.1612	5.03	0.17	17SA-12@05	*3235*	2.0547	4.80	0.19
PENGLAI@10		2.1146	5.01	0.16	17SA-12@03	*3235*	2.0652	4.91	0.19
PENGLAI@11		2.1140	4.85	0.16	17SA-12@02	*3235*	2.0626	5.11	0.19
PENGLAI@12		2.0953	5.09	0.17	*17SA-10-1*				
PENGLAI@13		2.1175	5.41	0.12	17SA-10@15	3453	2.1464	5.76	0.12
PENGLAI@14		2.1094	5.65	0.17	17SA-10@14	3453	2.1587	5.61	0.14
PENGLAI@15		2.1114	5.30	0.12	17SA-10@13	3453	2.1595	5.73	0.19
PENGLAI@16		2.0991	5.48	0.22	17SA-10@12	3453	2.1551	5.86	0.17
PENGLAI@17		2.0986	5.33	0.16	17SA-10@11	3453	2.1677	5.79	0.17
PENGLAI@18		2.0965	5.41	0.16	17SA-10@10	3453	2.1736	5.83	0.16
PENGLAI@19		2.0774	5.03	0.18	17SA-10@09	3453	2.1840	5.66	0.17
PENGLAI@20		2.0549	5.06	0.16	17SA-10@08	3453	2.1949	5.71	0.15
QINGHU@01		2.2772	5.60	0.21	17SA-10@07	3453	2.1952	5.33	0.09
QINGHU@02		2.2340	5.54	0.15	17SA-10@06	3453	2.2086	5.27	0.16
QINGHU@03		2.2040	5.60	0.17	17SA-10@05	3453	2.2151	5.83	0.13
QINGHU@04		2.1802	5.39	0.14	17SA-10@03	3453	2.2095	5.88	0.22
QINGHU@05		2.1111	5.30	0.16	17SA-10@02	3453	2.2040	5.92	0.13
QINGHU@06		2.1275	5.43	0.18	17SA-10@01	3453	2.2007	6.06	0.15

Supplementary Table S5 Compiled Re model ages (Ga) for mantle sulfides, alloy grains and peridotites

Sample No.	***T_{RD} (Ga)***	***Reference***	***Sample No.***	***T_{RD} (Ga)***	***Reference***
**Perdotite xenoliths from the Kaapvaal Craton**		Pearson et al., 1995a	**Peridoites from Siberian Craton**		Pearson et al., 1995b
*F865	2.8		UV49l76	3.2	
PHN1569	2.8		UV49/76	2.9	
PHN2600	3		UV251/86	3	
PHN2302	3.1		UV271/86	2.8	
PHN5239	3.5		**Eclogites from Siberian Craton**		Pearson et al., 1995c
**Peridotite xenoliths and kimberlita samples from southern African**		Walker et al., 1989	U5/91	3.4	
N. Lesotho			U281/84	3.2	
1596 WR	2.89		UV464	3.1	
**Peridotite xenoliths in the Monastery kimberlite from Eastern Kaapvaal mantle**		Carlson and Moore, 2004	U236/79	3.1	
Monastery	2.8		**Olivine-macrocryst from Siberian Craton**		Pearson et al., 2002
Newlands 7	2.81		Ol-61 (M)	2.98	
Newlands	2.83		Ol-63 (M)	3.52	
**Eclogitic sulfide inclusions in diamonds from Kimberley, South Africa**		Richardson et al., 2001	**Sulfide Inclusions in kimberlitic olivine from Siberian Craton**		Griffin et al., 2002
DP5	2.82		Ud-Ol-64-2	2.82	
DP6	2.9		Ud-Ol-67-2B	2.82	
DP9	2.99		Ud-Ol-67-6	2.82	
DP3	3.02		Ud-Ol-6	2.83	
DP4	3.02		Ud-Ol-67-3	2.84	
DP16	3.15		Ud-Ol-27	2.85	
DP9B	3.38		Ud-Ol-64-3	2.85	
DP1	4.08		Ud-Ol-36-2A	2.87	
DP18	4.15		Ud-Ol-48	2.88	
DP2	4.42		AOUd-Ol-61	2.89	
**Peridotite xenoliths in South Africa**		Menzies et al., 1999	Ud-Ol-50	2.92	
D10	2.97		Ud-Ol-2	2.94	
D11	3.18		Ud-Ol-36-2B	2.96	
C9	2.91		Ud-Ol-67-2D	2.96	
**Peridotite xenoliths in Kaapvaal and Zimbabwean Craton**		Carlson et al., 1999	Ud-Ol-19	3	
1419	2.8		Ud-Ol-9-2	3.04	
5252	2.81		Ud-Ol-1	3.1	
86	2.92		Ud-Ol-4	3.12	
73	2.98		Ud-Ol-14	3.12	
63	3.68		Ud-Ol-20	3.13	
**Kimberley in Kaapvaal Craton**		Simon et al., 2007	Ud-Ol-52	3.16	
K8	2.9		Ud-Ol-40	3.17	
K9	2.8		Ud-Ol-33	3.19	
K13	3		Ud-Ol-3	3.22	

Sample No.	*T_{RD} (Ga)*	*Reference*	*Sample No.*	*T_{RD} (Ga)*	*Reference*
K14	2.8		Ud-Ol-36	3.33	
K24	2.8		Ud-Ol-63	3.35	
K27	2.9		AOUd-Ol-60	3.44	
K12	2.8		**Canadian Craton**		Irvine et al., 2003
K18	3		K13B4	2.84	
**Peridotites from the Slave Craton**		Pearson et al., 2002	**Mantle xenoliths from East Greenland**		Hanghøj et al., 2001
8月7日	3		Nep 3	2.927	
10-12A	3		4–40	2.963	
**Olivine-macrocryst (M) and diamond (D) in Slave Craton**		Pearson et al., 2002	Nep 5	3.133	
VR481 (M)	2.86		Nep 1	3.764	
VR16367 (D)	3.03		**Peridotite xenoliths in W Greenland**		Bernstein et al., 2006
**Sulfide inclusions in mantle xenocrysts in Slave Craton**		Aulbach et al., 2004	452008	3.049	
C78	2.81		452002	3.275	
C185	2.82		**Peridotite xenoliths from S.W. Greenland**		Wittig et al., 2010
VR16367	2.97		474546	2.8	
C3	3		474574	2.802	
C25	3.01		488892	2.806	
C1	3.3		488850	2.813	
C41	3.9		474545	2.836	
**Sulfide inclusions and harzburgites in Slave Craton**		Westerlund et al., 2006	G-06-14A	2.841	
PD6a	2.8		474551	2.876	
PD7b	2.8		474576	2.876	
PD9c	2.8		474536	2.886	
PH15	2.8		474576	2.968	
PH16	2.8		488850	3.027	
PH17	2.8		**Mantle xenoliths in Tanzanian Craton**		Chesley et al., 1999
PH2	2.9		LB-11	3.4	
PH19	2.9		LB-1	4	
PH1	3.1		LB-14	3.25	
**Peridotite xenoliths from the North China Craton**		Gao et al., 2002	**Xenoliths from the NE Wyoming Craton**		Carlson and Irving, 1994
F50-9271	2.83		4	2.9	
**Sulfide phases in mantle xenoliths in South China Craton**		Wang et al., 2003	3	2.88	
KPH9816/3-1-s4	3.05				
